# Supplementary material for: Chromatin remodeling is required for sRNA‐guided DNA elimination in Paramecium
Source: EMBO J. 2022 Oct 11;41(22):e111839. doi: 10.15252/embj.2022111839 (PMC9670198; doi:10.15252/embj.2022111839)
Supplement: Supplementary file 1 — Appendix [file EMBJ-41-e111839-s010.pdf]

Appendix

Table of Contents **Page number**

Table of Contents	Page number
Appendix Table S1	2
Appendix Table S2	3
Appendix Table S3	4

Appendix Table S1

IES	Primer sequence (5' to 3' orientation)
51G--11F	ATCATAAGATTGATATCTTCTCCCTTCTCC
51G--11R	ACTTGCTACTAAAGCAAGAAACATTGAGAG
51G1413F	GAAGCTGCTTGTGTTAAGAATTCTACTGG
51G1413R	GCATCCAGCACTAGTTGAATTTACTGTAC
51G1832F	CTATAACTCTTGAAGCTGCTTGTAATATG
51G1832R	TTGTCAATGAGCCATTAACAGTTGCTGGAT
51G2832F	GAGCAGGATGTACAAATACTGGTGG
51G2832R	AGCTGATTAGATAACAATACAACCAGTACC
51G4404F	CTGTTGCTACACATTGTGCATATGTTACT
51G4404R	GCTGTAAGATTAACATTGAGCATGATCAAG
51G6447F	AATGCATCAAATGTAGTAACTACTCCTGCT
51G6447R	AATTTGTAAAGTATCCAGCGCAGGCAG
MT Locus F	GGTGTTTATATCTTAATTGTTGACCCTCAC
MT Locus R	CCATCTATACTCCATTCTTTATCTTAATTCAT
51A--712F	TTTGTCAAAAAGACATGTATCAAAATGCAG
51A--712R	TAGAATACTAAGAGATTCAATACAACAAAC
51A1835F	TAATGTATTGATAAGGCTTGCTCTACAGCC
51A1835R	ATCCTAACATCCTTGAATAGTTACTGATCC
51A2591F	ATGTGTTTGGACTGGATTGGCATGTAGAAG
51A2591R	GATGTAGCATAACATTTATCAACAATCCAT
51A4404F	TGGAATAGTGCTGCATCACCAGCTGCTTGC
51A4404R	CCAGTTATTGAACTGCAACTTACTGCAGTG
51A4578F	CACTGCAGTAAGTTGCAGTTCAATAACTGG
51A4578R	TGTAGTCTTAAAATCTTAGCATGTTGTACC
51A6435F	CAAATTGTGTCACTAGAGGTACATGTTTCC
51A6435R	GCGACATCAATAGTAACAGCTGAGCATGAG
51A6649F	ACTGCACCTCTAACTTTAACAAGCGAAGCA
51A6649R	CAGCAGTACATCCAGCTCTCTAAGTTTAGC
51-429F	GTT GGA TAT GCA TCC ACA TC
51-429R	CTG CTT CGA TAT GCA TAA GAA AG

Appendix Table S1: Primers used in IES retention PCRs. F: forward primer; R: reverse primer.

Appendix Table S2

Majority protein IDs	MS/MS count ISWI1	MS/MS count ISWI2	MS/MS count WT1	MS/MS count WT2	Entrez_Protein	Putative Description
PTET.51.1.P0140243	243	261	0	0	XP_001431605	ISWI1 (this manuscript)
PTET.51.1.P0440186	90	104	0	0	XP_001447805.1	Uncharacterized
PTET.51.1.P0140243	58	68	0	0	XP_001431606	ISWI1 (C-terminal)
PTET.51.1.P1370127	62	61	0	0	XP_001431060	DDRP
PTETP2700007001	61	50	0	0	XP_001441411	NAD () ADP-ribosyltransferase-3
PTETP300037001	49	45	0	0	XP_001442677	SPT6
PTET.51.1.P0420126	42	49	0	0	XP_001447124	ISWI2 (this manuscript
PTETP7100004001	26	43	2	3	XP_001456124	PTIWI01
PTET.51.1.P0180124	33	36	0	0	XP_001437349	Uncharacterized
PTET.51.1.P0070284	39	28	0	0	XP_001455634	Poly (ADP-ribose) Polymerase
PTET.51.1.P0480005	34	29	2	0	XP_001448777	DDRP2
PTETP10500011001	31	33	0	0	XP_001424861	SPT16
PTET.51.1.P0110326	19	26	3	8	XP_001425954	S-adenosyl methionine synthetase
PTET.51.1.P0330075	26	20	0	0	XP_001443869	SMC2
PTET.51.1.P0560063	17	22	2	2	XP_001451492	RuvB-like 2
PTET.51.1.P0720184	19	20	0	1	XP_001456414	Exportin
PTET.51.1.P0990120	17	22	0	0	XP_001462544	MCM6
PTETP13700005001	19	17	0	0	XP_001431029	PARP
PTET.51.1.P0040036	18	17	0	0	XP_001446004	sequence specific DNA binidng
PTET.51.1.P1720081	19	15	0	0	XP_001436697	Myb-like protein
PTET.51.1.P0610231	17	16	0	0	XP_001453395	structural constituent of ribososme
PTET.51.1.P0380048	17	15	0	0	XP_001445418	SPT5v
PTET.51.1.P0200322	14	16	0	0	XP_001439097	PHD-type
PTET.51.1.P1060046	11	16	0	0	XP_001425020	WD40-repeat like
PTET.51.1.P0030037	9	15	2	1	XP_001442334	RuvB-like 1
PTET.51.1.P1600114	9	16	0	0	XP_001435111	Erythrocyte membrane protein 3
PTET.51.1.P0470202	10	15	0	0	XP_001448725	Helicase W08D27
PTET.51.1.P0410183	12	13	0	0	XP_001446881	Uncharacterized protein
PTET.51.1.P1180147	7	16	0	0	XP_001427491	MCM2
PTET.51.1.P006001	9	10	3	0	XP_001452484	Uncharacterized protein
PTET.51.1.P0620215	10	11	0	0	XP_001453643	PTIWI11
PTET.51.1.P0170077	11	9	0	0	XP_001449252	NOWA2
PTET.51.1.P0400053	12	8	0	0	XP_001436180	DNA directed DNA polymerase
PTET.51.1.P0450314	6	12	0	0	XP_001448195	Uncharacterized protein
PTET.51.1.P0080258	8	10	0	0	XP_001458158	Uncharacterized protein

Appendix Table S2: Mass spectrometry analysis of ISWI1-3XFLAGHA co-immunoprecipitation (IP). Majority protein IDs correspond to the *Paramecium* Database (Arnaiz and Sperling, 2011) accession numbers of the proteins identified by MS. MS/MS count ISWI1 & MS/MS count ISWI1 represents total peptide count in ISWI1-3XFLAGHA IP replicates. MS/MS count WT1 & MS/MS WT2 represents total peptide count in negative control to IP. MS/MS count represents combined peptide counts in the replicates. Putative description is retrieved from Paramecium Database (https://paramecium.i2bc.paris-saclay.fr/)

Appendix Table S3

IES ID	IES Name	*PGM* -KD	*DCL2/3* -KD	*DCL5*KD	*DCL2/3/5* -KD	*NOWA1/2* -KD	*ISWI1* -KD
IESPGM.PTET51.1.51.451201	51G-11	0.79	0.05	0.19	0.56	0.53	0.48
IESPGM.PTET51.1.51.452624	51G1413	0.78	0.01	0.01	0.04	0.15	0.07
IESPGM.PTET51.1.51.453043	51G1832	0.73	0.00	0.00	0.02	0.02	0.04
IESPGM.PTET51.1.51.454043	51G2832	0.77	0.22	0.00	0.71	0.59	0.20
IESPGM.PTET51.1.51.455615	51G4404	0.84	0.60	0.00	0.75	0.80	0.58
IESPGM.PTET51.1.51.457658	51G6447	0.77	0.04	0.00	0.00	0.05	0.08
IESPGM.PTET51.1.106.281631	51A-712	0.78	0.07	0.05	0.71	0.75	0.62
IESPGM.PTET51.1.106.284157	51A1835	0.80	0.00	0.00	0.04	0.03	0.03
IESPGM.PTET51.1.106.284913	51A2591	0.91	0.54	0.00	0.80	0.81	0.68
IESPGM.PTET51.1.106.286750	51A4404	0.82	0.00	0.00	0.00	0.00	0.23
IESPGM.PTET51.1.106.286924	51A4578	0.77	0.04	0.00	0.07	0.20	0.10
IESPGM.PTET51.1.106.288781	51A6435	0.77	0.00	0.00	0.00	0.01	0.04
IESPGM.PTET51.1.106.288995	51A6649	0.81	0.55	0.01	0.73	0.73	0.61

Appendix Table S3: Comparison of IES retention scores. IESs are those that were used to test retention using standard primers (Table T1) against IESs described in (Duharcourt et al. 1998).
