# Supplementary material for: Chromatin remodeling is required for sRNA‐guided DNA elimination in Paramecium
Source: EMBO J. 2022 Oct 11;41(22):e111839. doi: 10.15252/embj.2022111839 (PMC9670198; doi:10.15252/embj.2022111839)
Supplement: Supplementary file 2 — Expanded View Figures PDF [file EMBJ-41-e111839-s007.pdf]

Expanded View Figures

Figure EV1. Knockdown effects of ISWI paralogs in *Paramecium tetraurelia*.

A, B Northern blot analysis using ISWI1-specific and ISWI5-specific probes, respectively. rRNA probe was used as a loading control against ribosomal RNA. Early: ~50% of cells with fragmented parental macronucleus; Late: the majority of cells with a visible anlagen. *ND7*-KD is used as a control to confirm mRNA expression.

C–F (C and E) Survival test graph. Dead cells are represented in red, sick in orange and cells diving at a normal rate in green. *PGM*–KD is used as a positive control, and *ND7*–KD as a negative control. (D and F) IES retention PCR (cropped inverted images). Five maternally controlled IES and five non-maternally controlled IESs are shown. The IES+ band represents retained IES; the IES-band represents excised IES; additional bands are likely PCR artifacts or primer dimers.

Source data are available online for this figure.

© 2022 The Authors

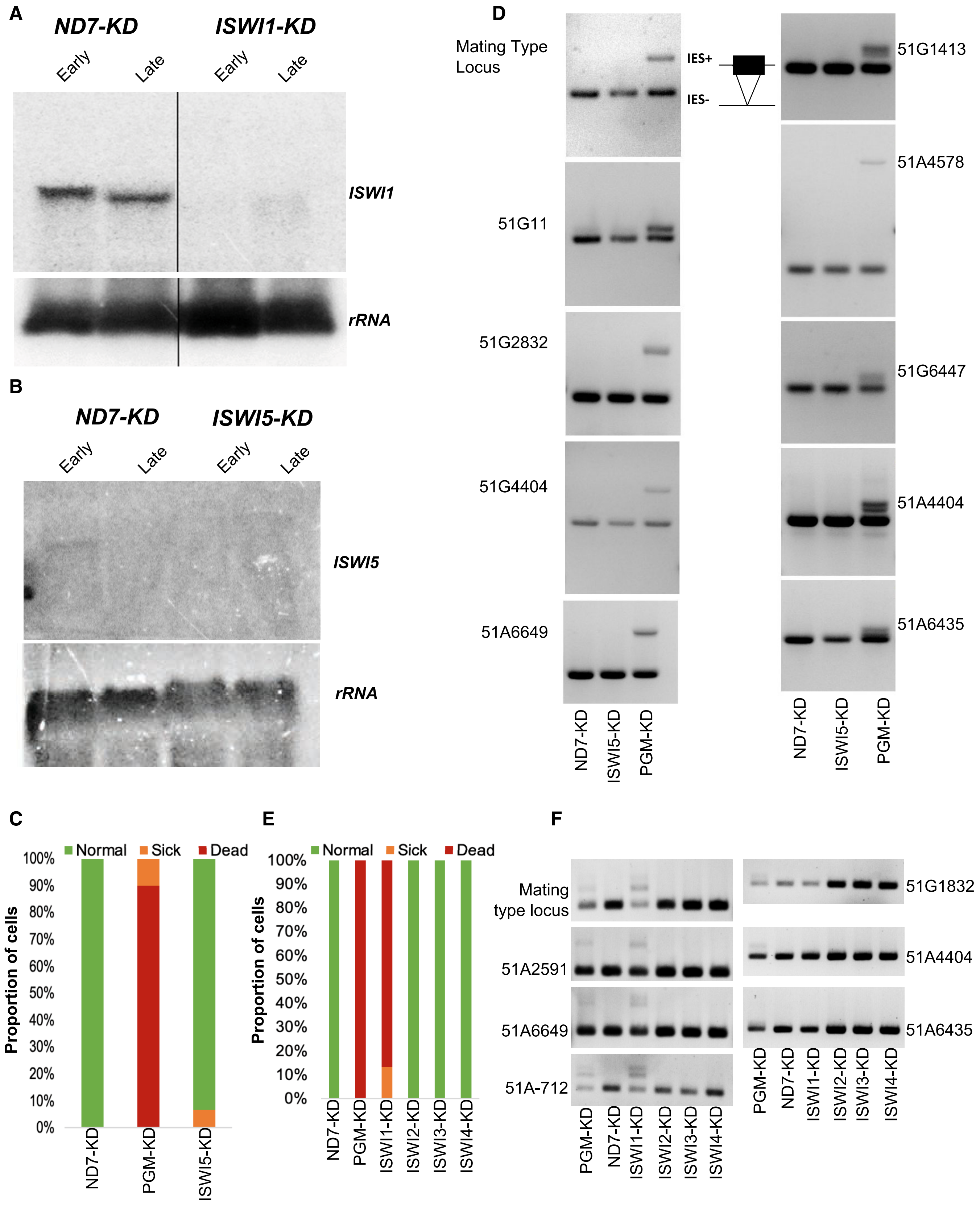

Figure EV1.

 © 2022 The Authors

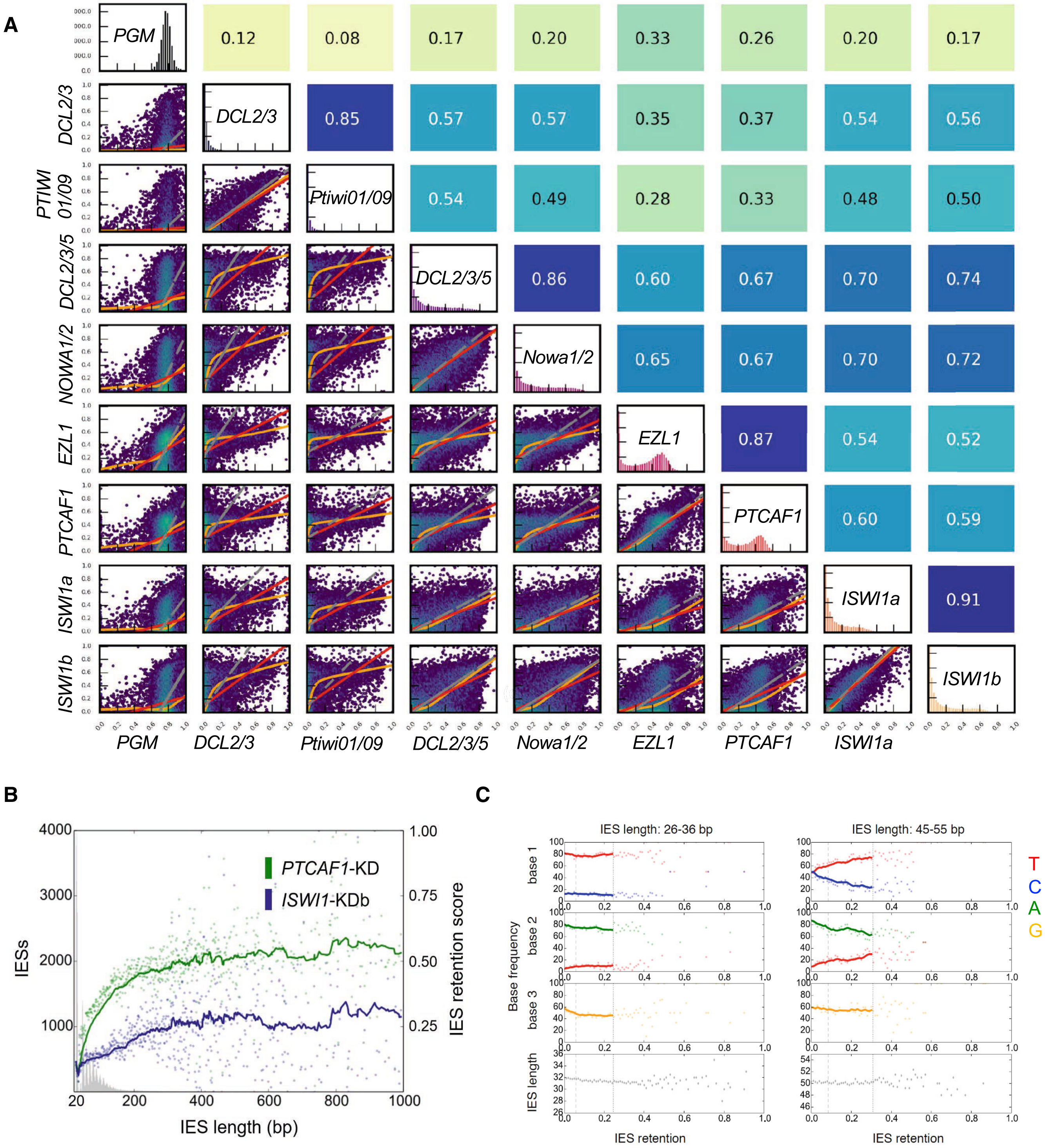

Figure EV2. Relationship between IES retention scores, IES length, and base sequences.

A Relationships in IES retention among knockdown pairs. Hexagonal binning of IES retention scores was used to generate the plots. Pearson's correlation coefficients are given above each subgraph. Red lines are for ordinary least-squares (OLS) regression, orange lines are for LOWESS, and gray lines are for orthogonal distance regression (ODR).

B IRSs versus IES length as described previously.

C Base frequencies of the first three bases after the TA repeat relative to the IRS of *ISWI1*-KDb from the first and third *Paramecium tetraurelia* IES length peak.

© 2022 The Authors

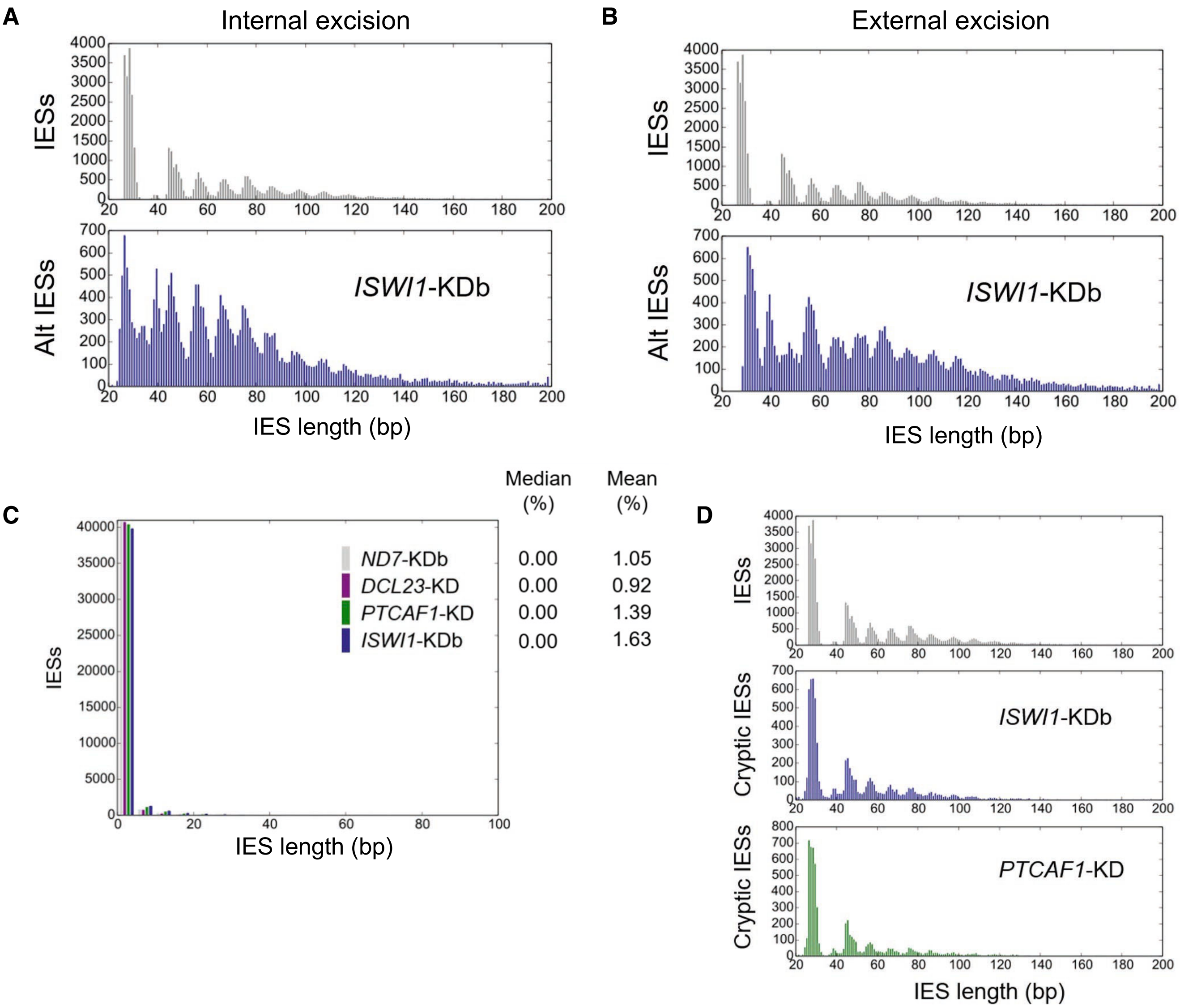

Figure EV3. Size distribution of Alternatively excised IESs and Cryptic IESs.

A Length distribution of internally excised alternative (Alt) IES boundaries.

B Length distribution of externally alternative (Alt) excised IES boundaries, respectively.

C Genome-wide analysis of cryptic IES excision. Cryptic excision (%) = 100*(cryptically excised reads)/(all reads).

D Length distribution of cryptically excised IES.

 © 2022 The Authors

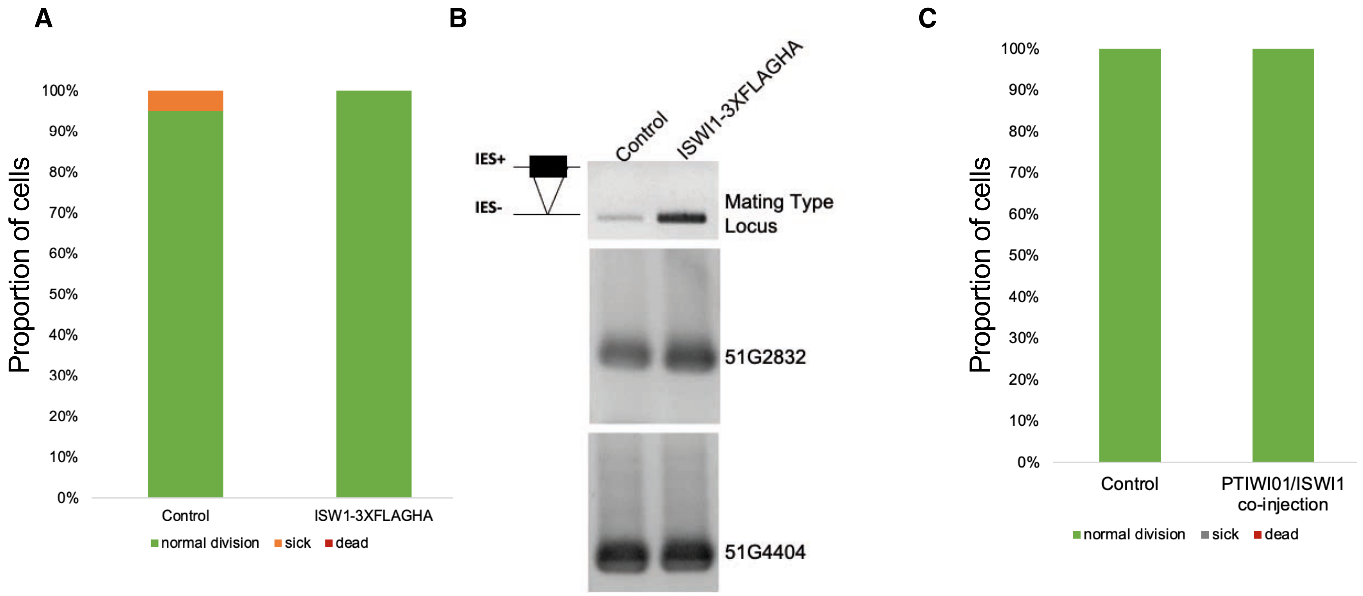

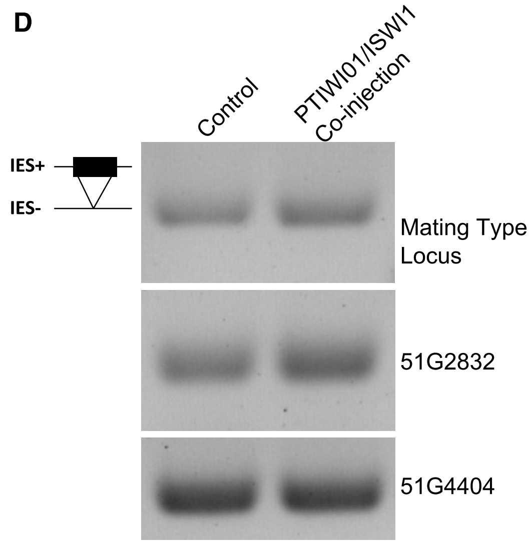

E

Majority protein IDs	Peptide Count ISWI1	Peptide count ISWI2	Peptide count WT1	Peptide count WT2	*p* BH adjusted
ISWI1	301	329	0	0	n.d.
Uncharacterized protein	90	104	0	0	n.d.
DDRP	62	61	0	0	n.d.
SPT6	49	45	0	0	n.d.
PTIWI01	26	43	2	3	0,004
NOWA2	11	9	0	0	n.d.

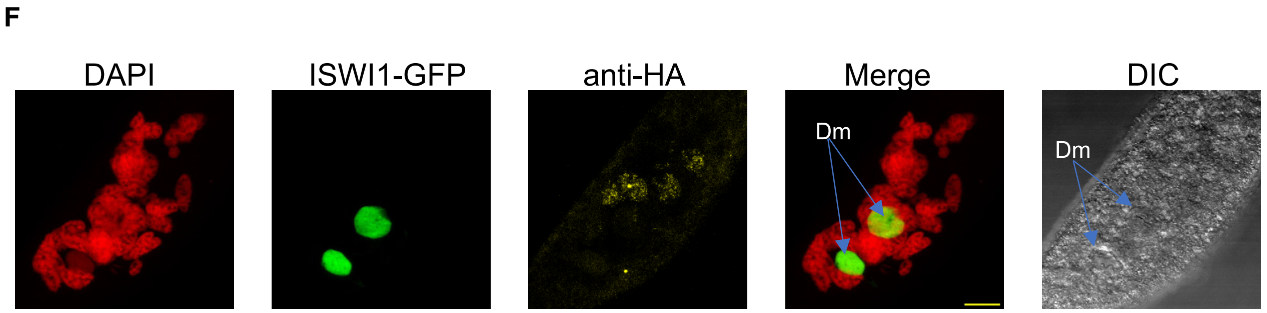

Figure EV4.

Figure EV4. Overexpression of fusion proteins do not show any adverse effects.

A–D (A and C) Survival test graph. Dead cells are represented in red, sick in orange and cells diving at a normal rate in green. (B and D) IES retention PCR (cropped inverted images). Mating type, 51G2832, and 51G4404 IESs are shown. The IES+ band represents retained IES; the IES-band represents excised IES; additional bands are likely PCR artifacts or primer dimers.

E Most abundant proteins in the ISWI1-3XFLAGHA MS analysis ISWI1 & ISWI2 are two biological replicates for ISWI1-3XFLAGHA, while WT1 and WT2 are biological replicates for control in MS analysis. Peptide count refers to the number of peptides detected in MS. Adjusted *P*-values were calculated following the Benjamini and Hochberg correction for multiple testing.

F ISWI1-GFP localization to developing macronucleus seen in green during development; merge is an overlay of DAPI (red) staining parental and developing macronucleus, ISWI1-GFP (green), and anti-HA (yellow); scale bar = 10 μm.

Source data are available online for this figure.

Figure EV5. Nucleosome density measurements after DNase-seq.

A IES Retention Score (IRS) distributions for *PGM/ND7*-KD and *PGM/ISWI1*-KD. (B, C) Histograms of outer paired-end distances of mapped DNase-seq reads.

D–I Normalized nucleosome density histograms for IESs weakly (IRS < 0.2) or more strongly retained in *ISWI1*-Kdb (IRS ≥ 0.2), either for *ND7/PGM*-KD or *NOWA1/PGM*-KD. Kolmogorov–Smirnov statistics and their *P*-values are provided. Titles for graphs give criteria for IES selection.

Source data are available online for this figure.

 © 2022 The Authors

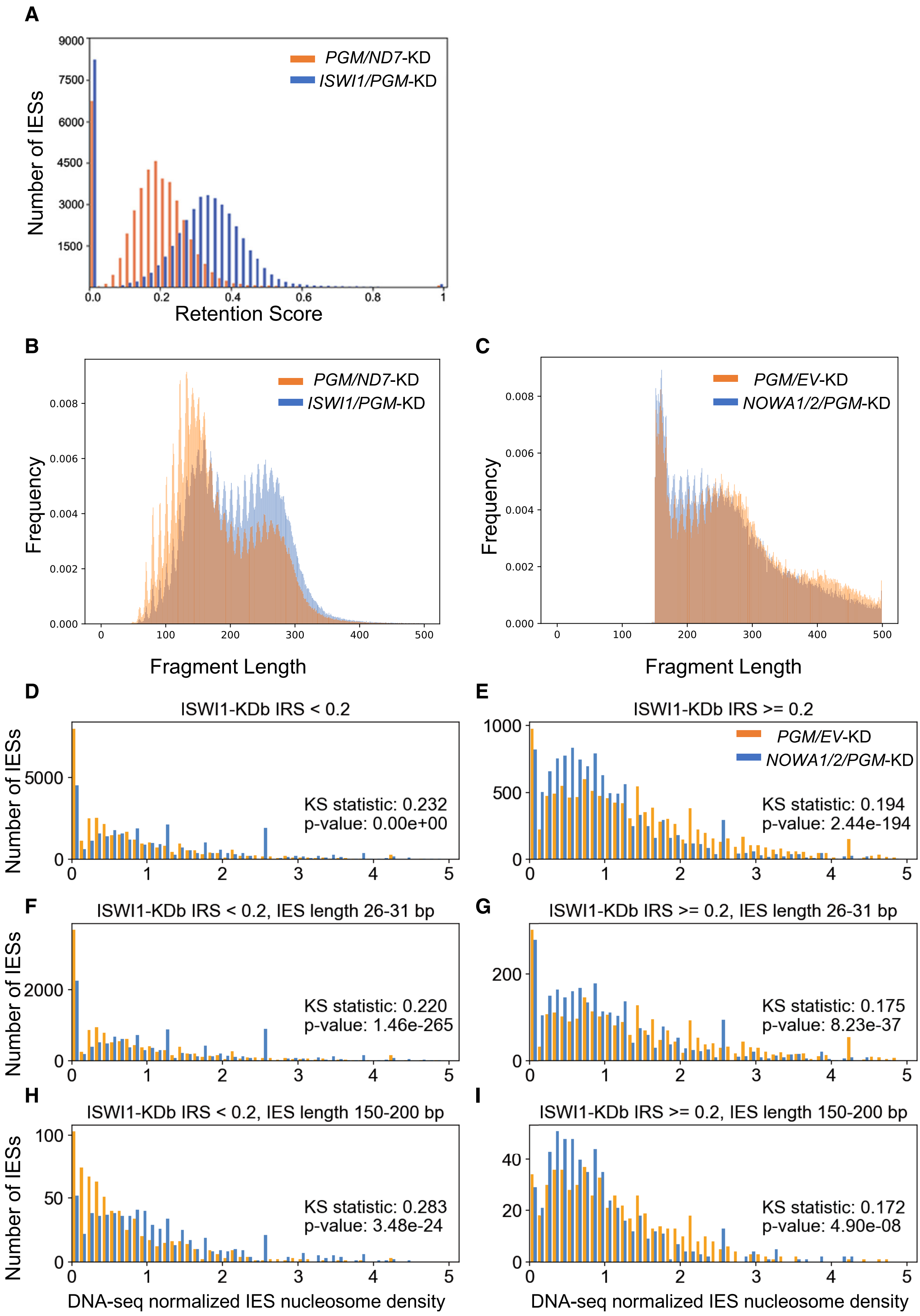
A
PGM/ND7-KD
ISWI1/PGM-KD
Number of IESs
Retention Score
B
Frequency
Fragment Length
C
PGM/EV-KD
NOWA1/2/PGM-KD
Frequency
Fragment Length
D
ISWI1-KDb IRS < 0.2
KS statistic: 0.232
p-value: 0.00e+00
E
ISWI1-KDb IRS >= 0.2
KS statistic: 0.194
p-value: 2.44e-194
F
ISWI1-KDb IRS < 0.2, IES length 26-31 bp
KS statistic: 0.220
p-value: 1.46e-265
G
ISWI1-KDb IRS >= 0.2, IES length 26-31 bp
KS statistic: 0.175
p-value: 8.23e-37
H
ISWI1-KDb IRS < 0.2, IES length 150-200 bp
KS statistic: 0.283
p-value: 3.48e-24
I
ISWI1-KDb IRS >= 0.2, IES length 150-200 bp
KS statistic: 0.172
p-value: 4.90e-08
Number of IESs
DNA-seq normalized IES nucleosome density

Figure EV5.
