# Supplementary figures and images for: Chromatin remodeling is required for sRNA‐guided DNA elimination in Paramecium
Source: EMBO J. 2022 Oct 11;41(22):e111839. doi: 10.15252/embj.2022111839 (PMC9670198; doi:10.15252/embj.2022111839)

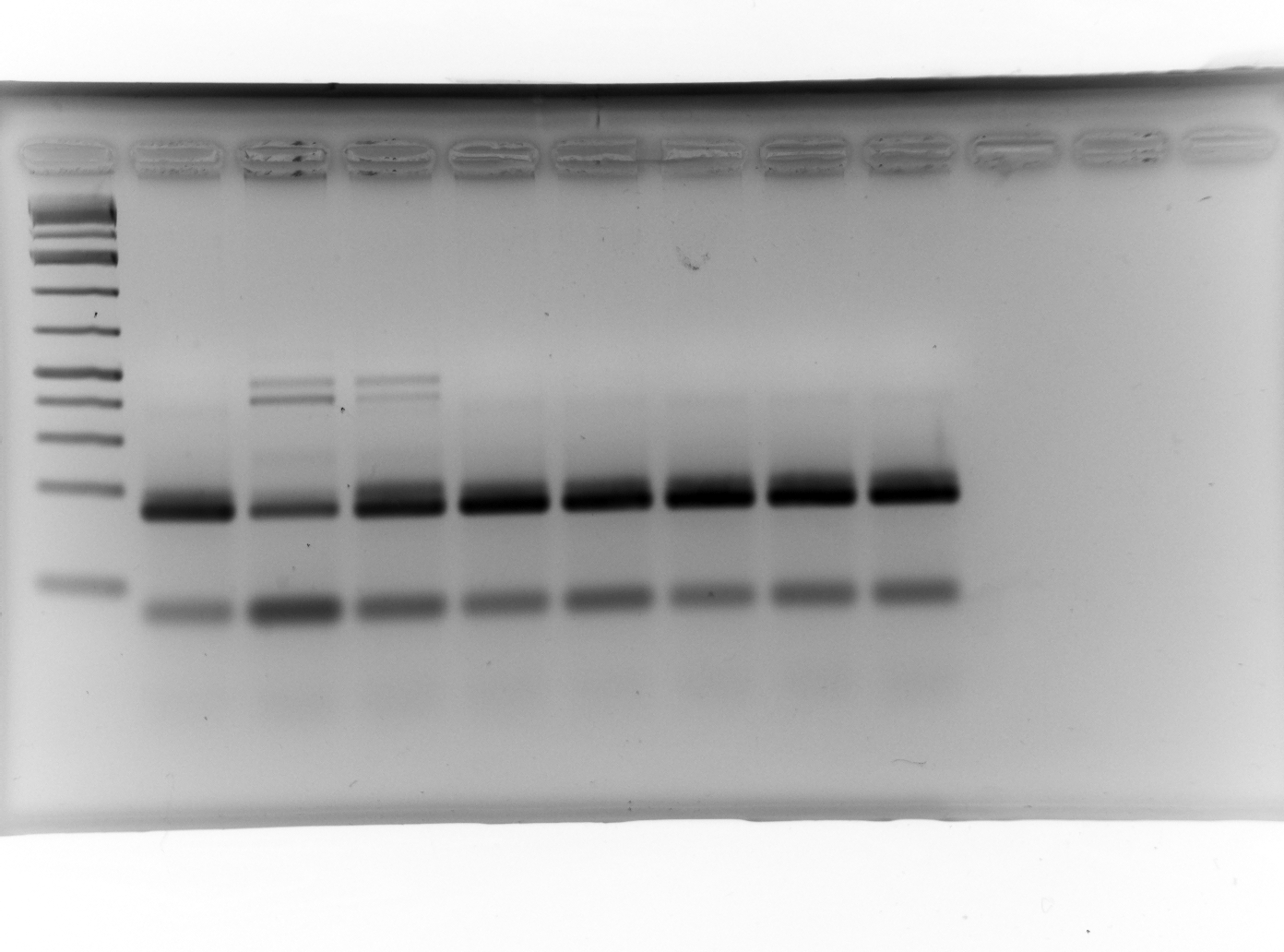

Supplement: Supplementary file 10 — Source Data for Figure 1 [file EMBJ-41-e111839-s012.zip › SourceData_Figure1/Figure1FSourceData2.tif]

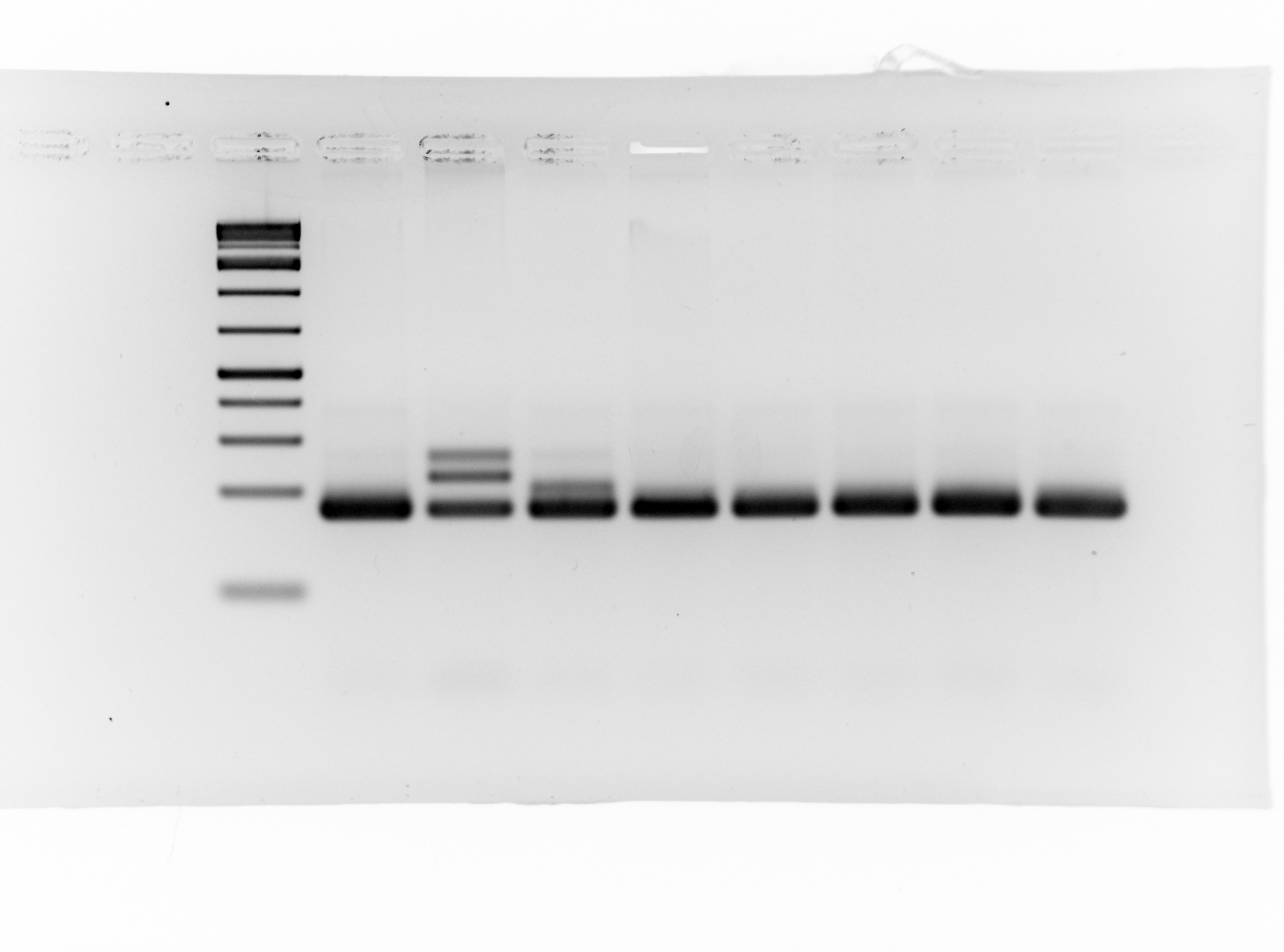

Supplement: Supplementary file 10 — Source Data for Figure 1 [file EMBJ-41-e111839-s012.zip › SourceData_Figure1/Figure1FSourceData3.tif]

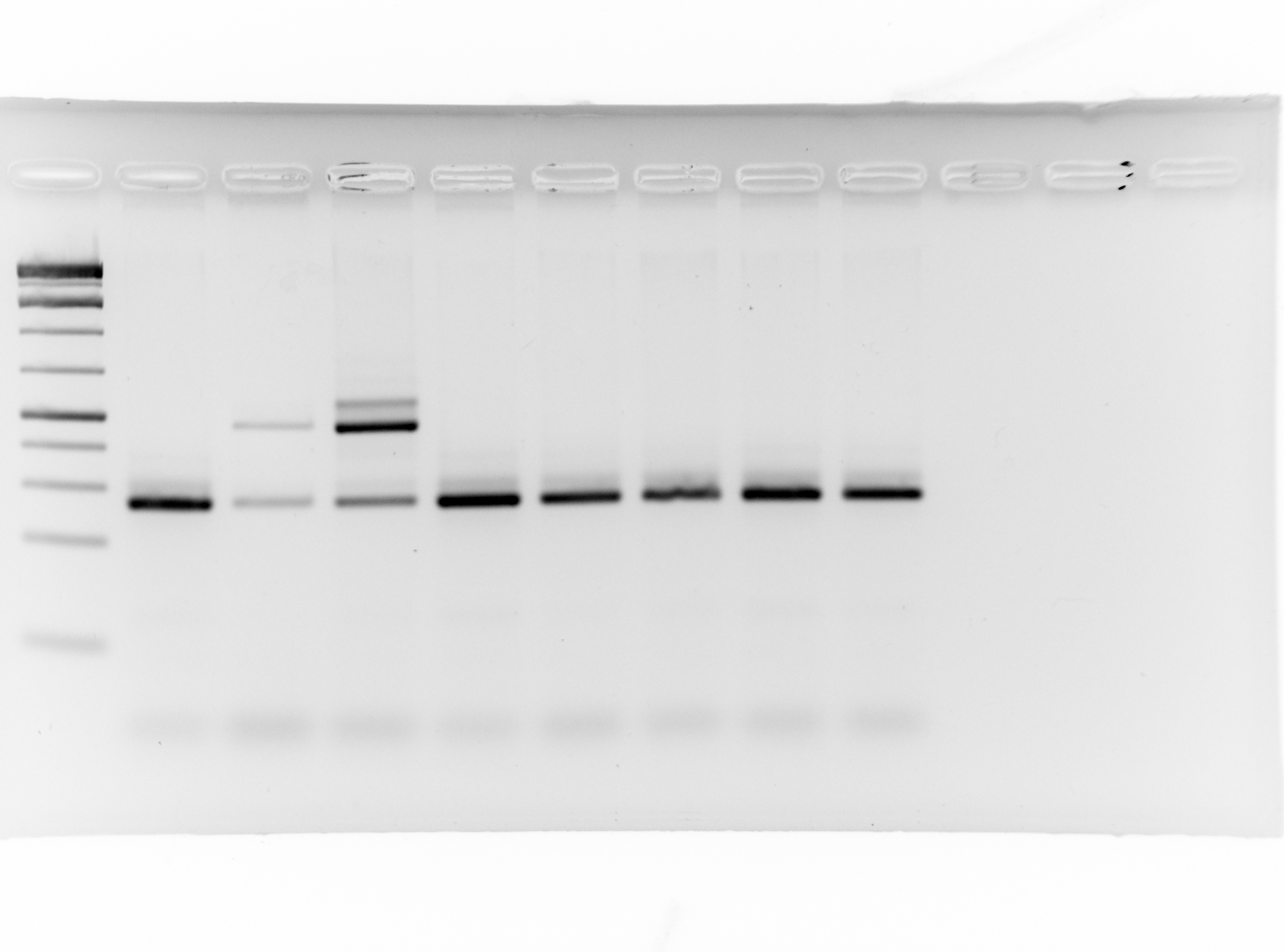

Supplement: Supplementary file 10 — Source Data for Figure 1 [file EMBJ-41-e111839-s012.zip › SourceData_Figure1/Figure1FSourceData1.tif]

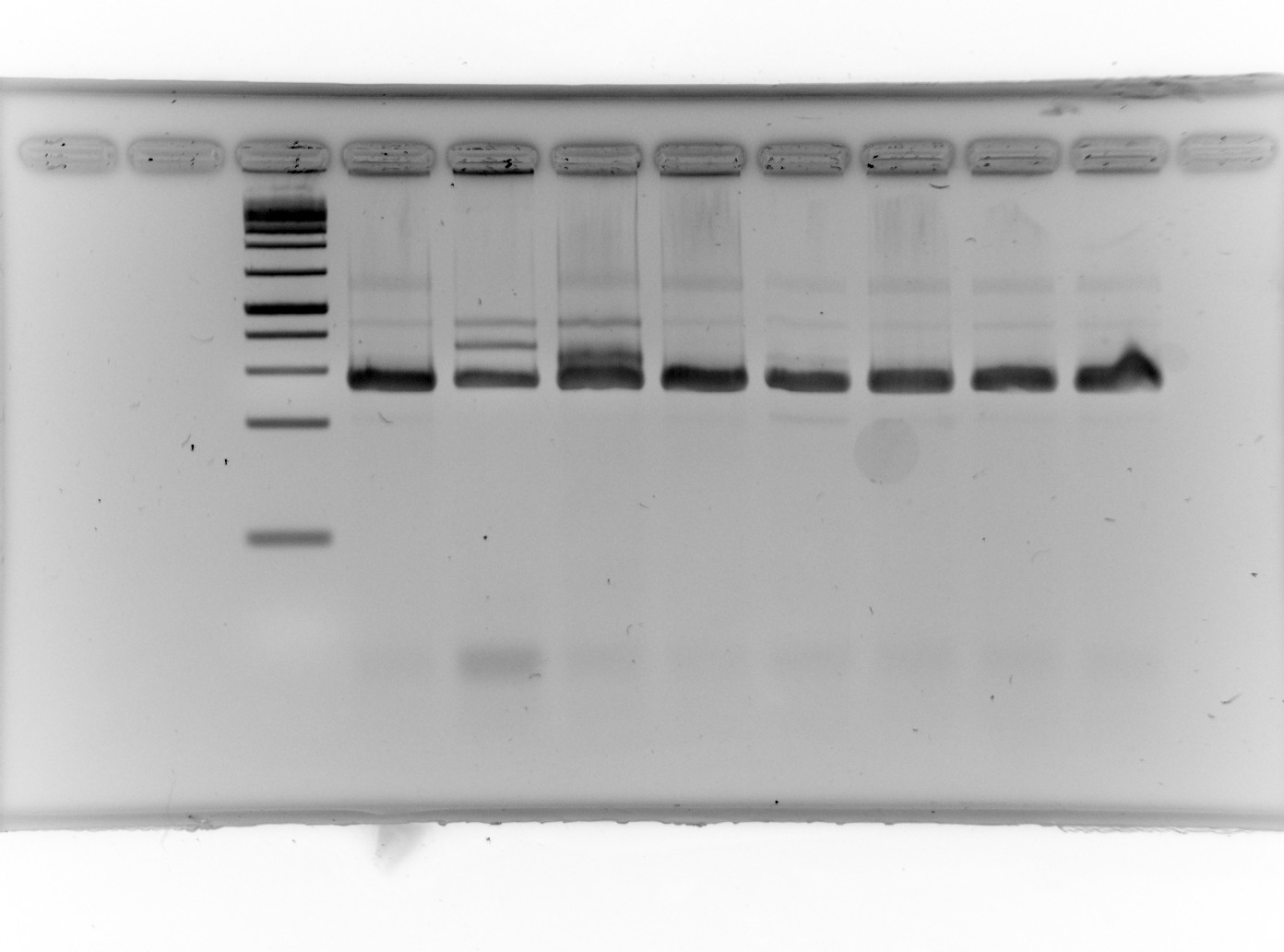

Supplement: Supplementary file 10 — Source Data for Figure 1 [file EMBJ-41-e111839-s012.zip › SourceData_Figure1/Figure1FSourceData5.tif]

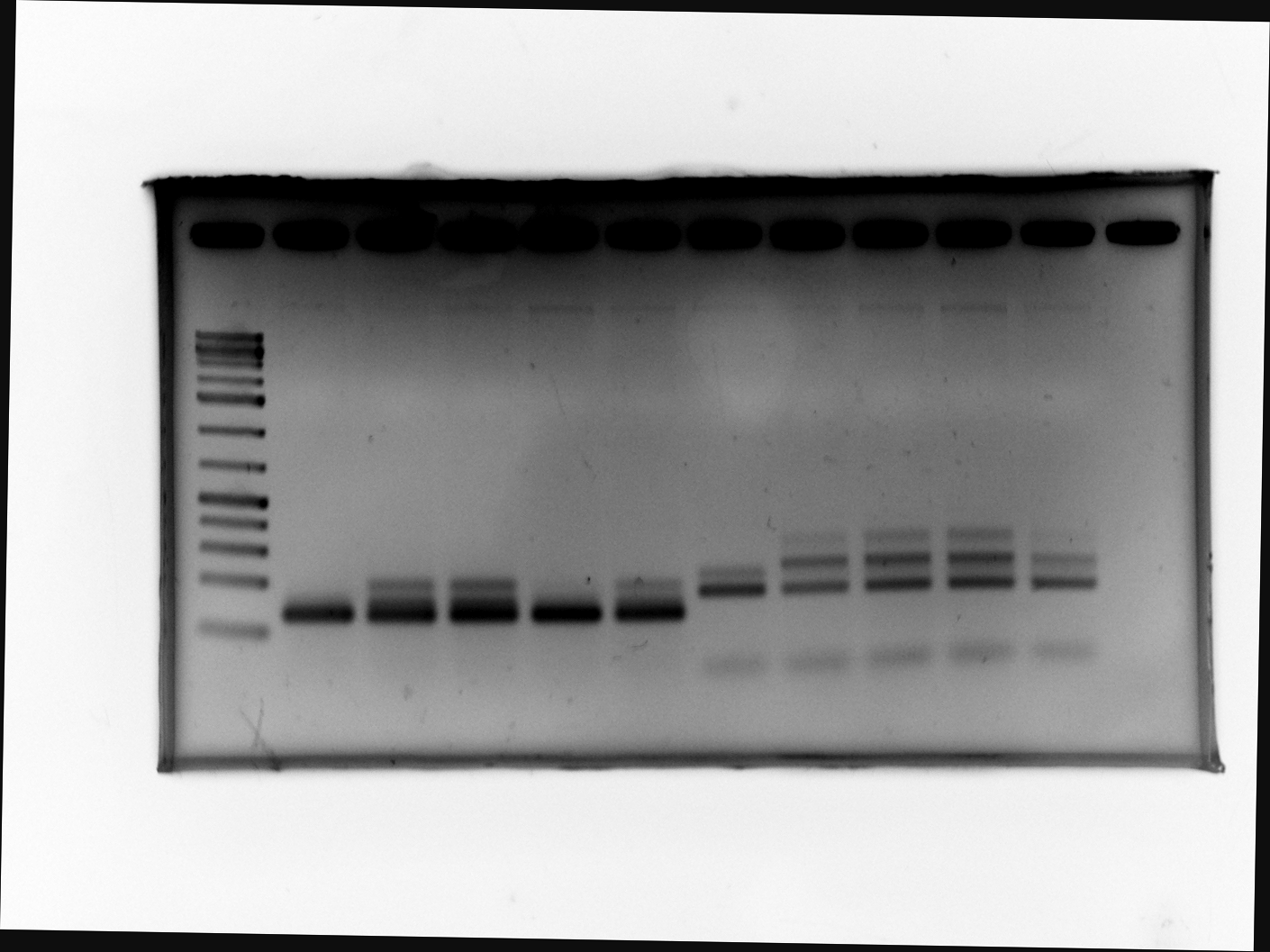

Supplement: Supplementary file 10 — Source Data for Figure 1 [file EMBJ-41-e111839-s012.zip › SourceData_Figure1/Figure1FSourceData7.tif]

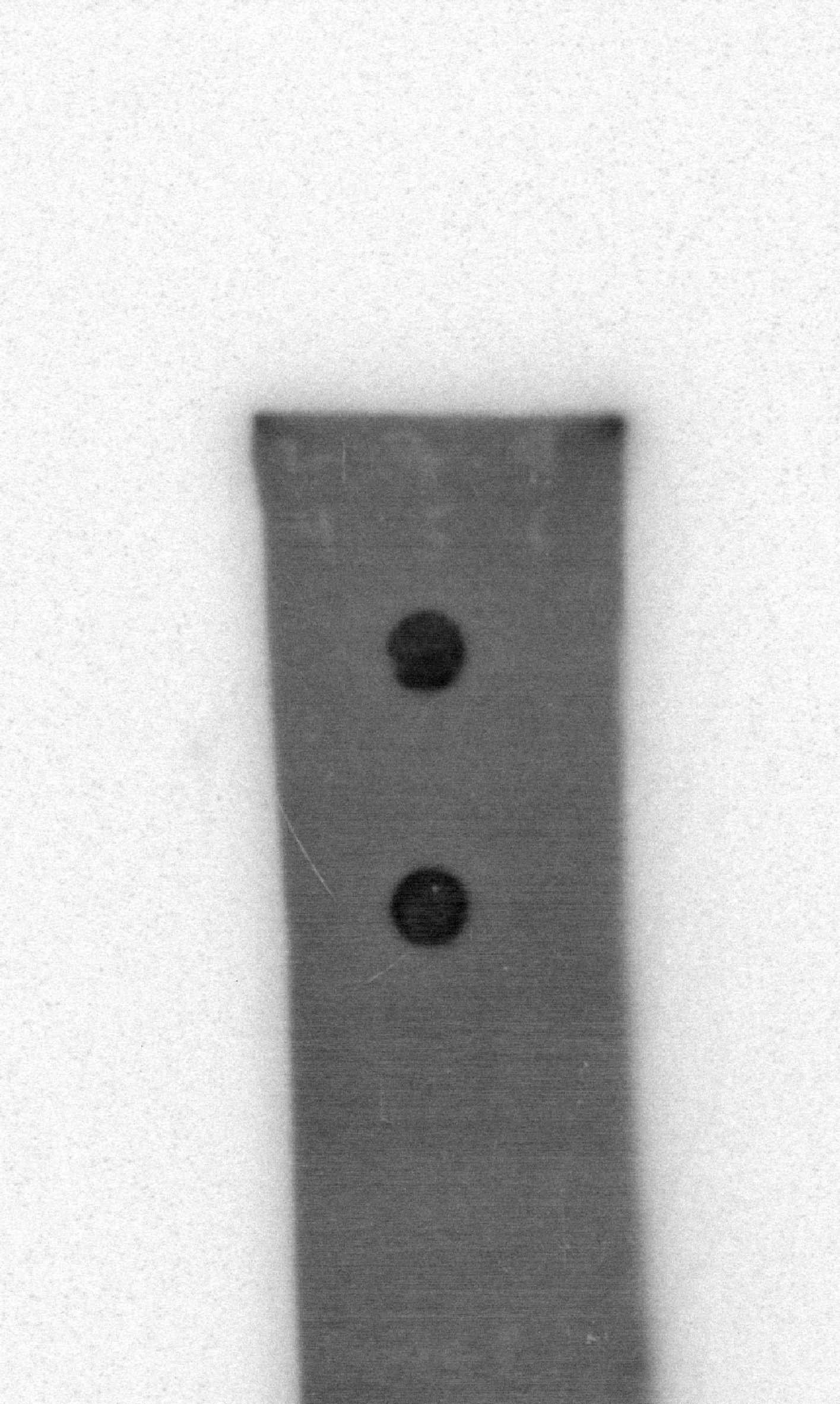

Supplement: Supplementary file 10 — Source Data for Figure 1 [file EMBJ-41-e111839-s012.zip › SourceData_Figure1/Figure1ESourceData10.bmp]

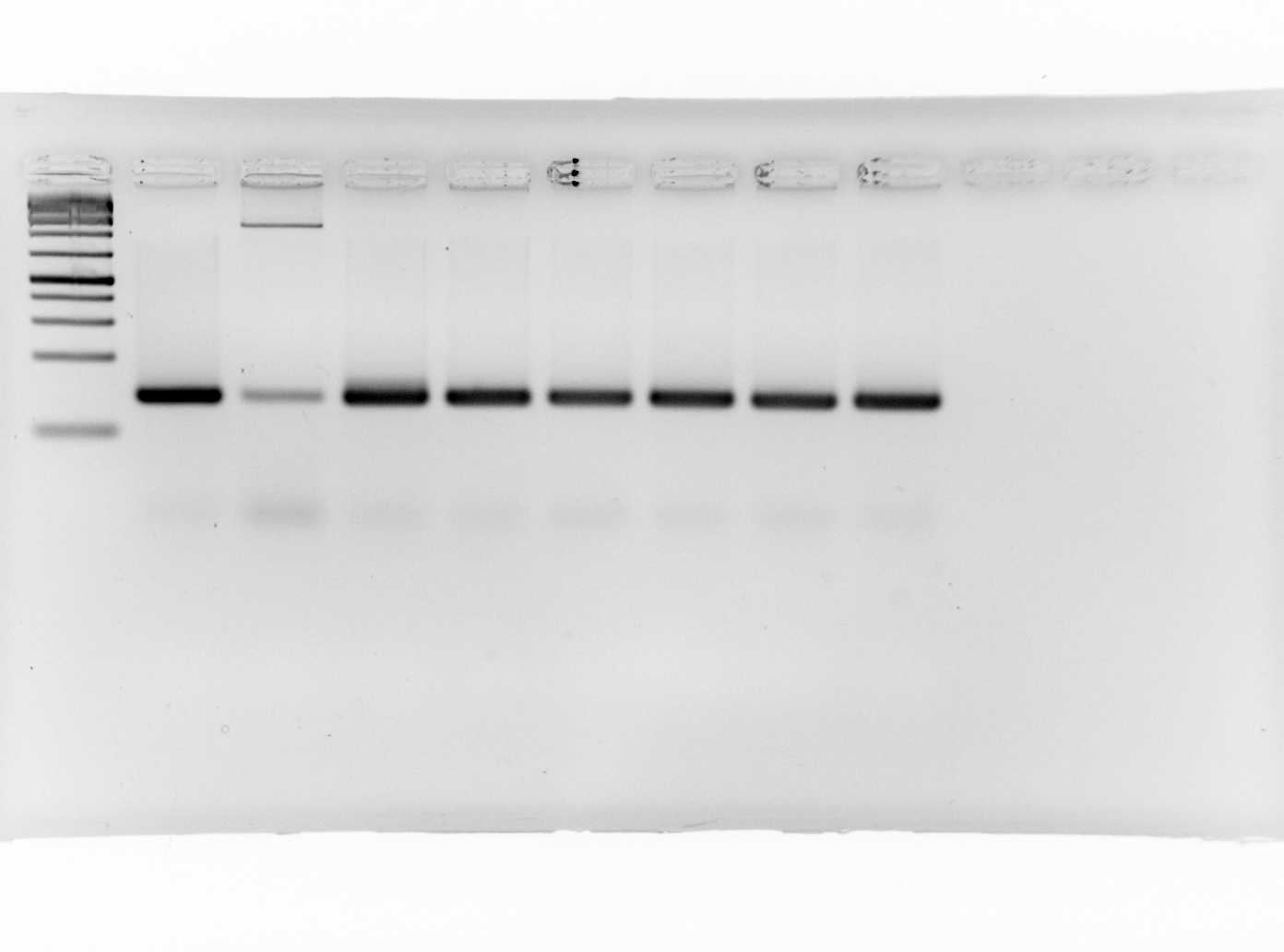

Supplement: Supplementary file 10 — Source Data for Figure 1 [file EMBJ-41-e111839-s012.zip › SourceData_Figure1/Figure1FSourceData6.tif]

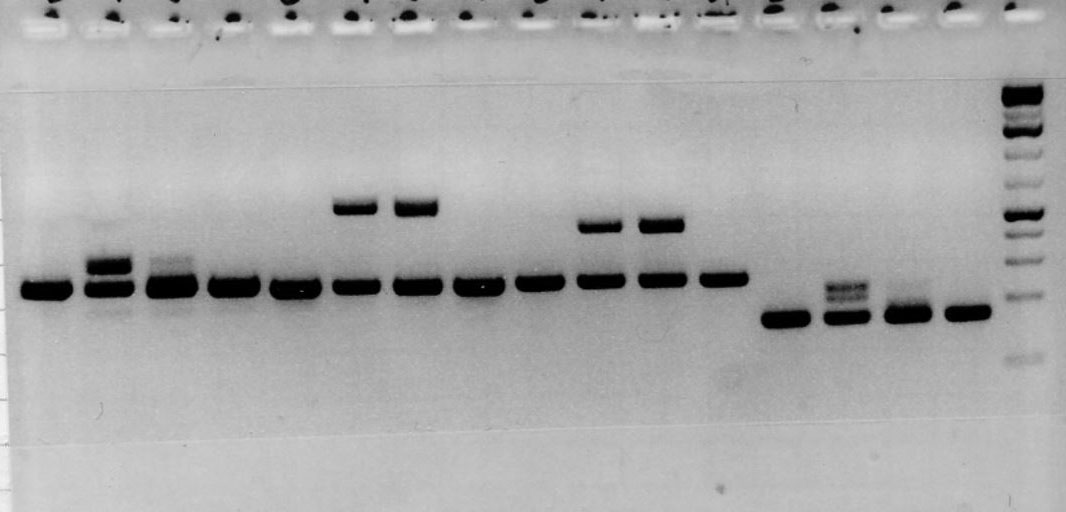

Supplement: Supplementary file 10 — Source Data for Figure 1 [file EMBJ-41-e111839-s012.zip › SourceData_Figure1/Figure1FSourceData4.JPG]

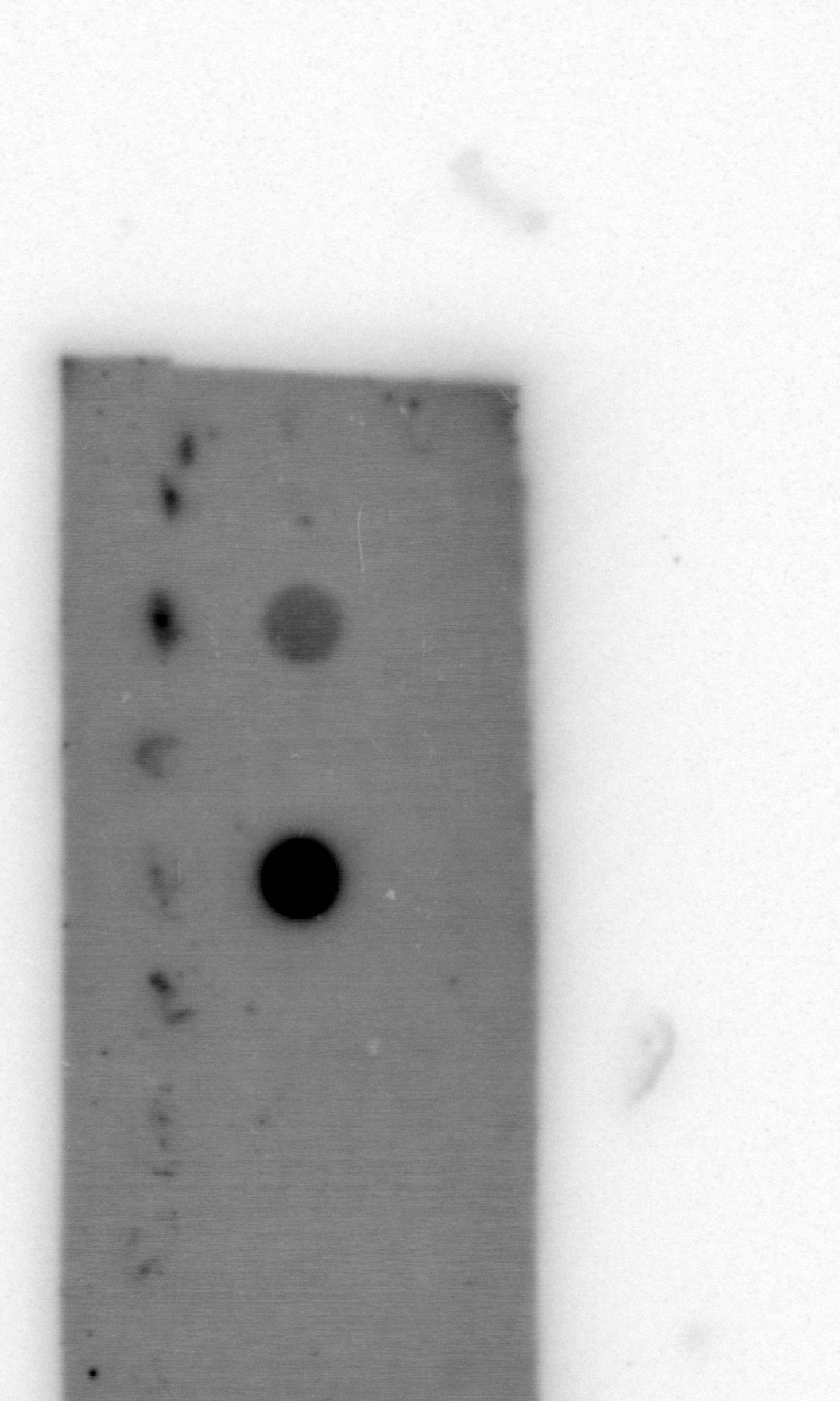

Supplement: Supplementary file 10 — Source Data for Figure 1 [file EMBJ-41-e111839-s012.zip › SourceData_Figure1/Figure1ESourceData9.bmp]

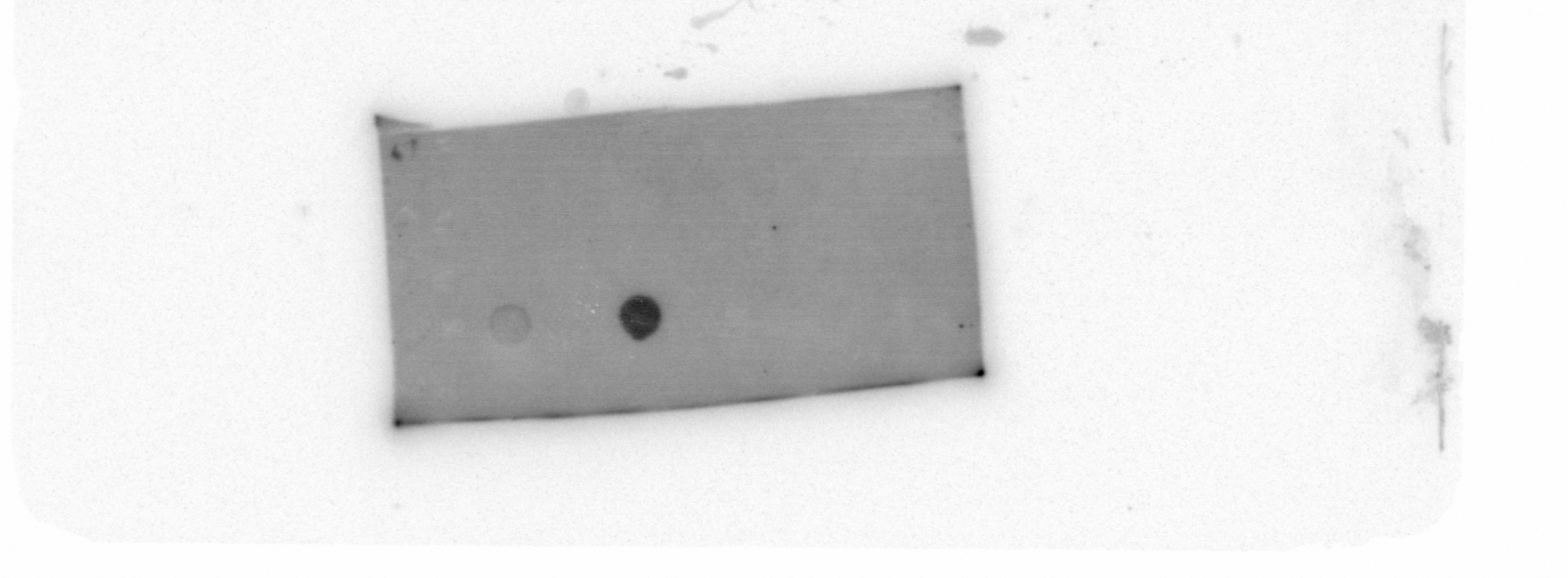

Supplement: Supplementary file 10 — Source Data for Figure 1 [file EMBJ-41-e111839-s012.zip › SourceData_Figure1/Figure1ESourceData8.bmp]

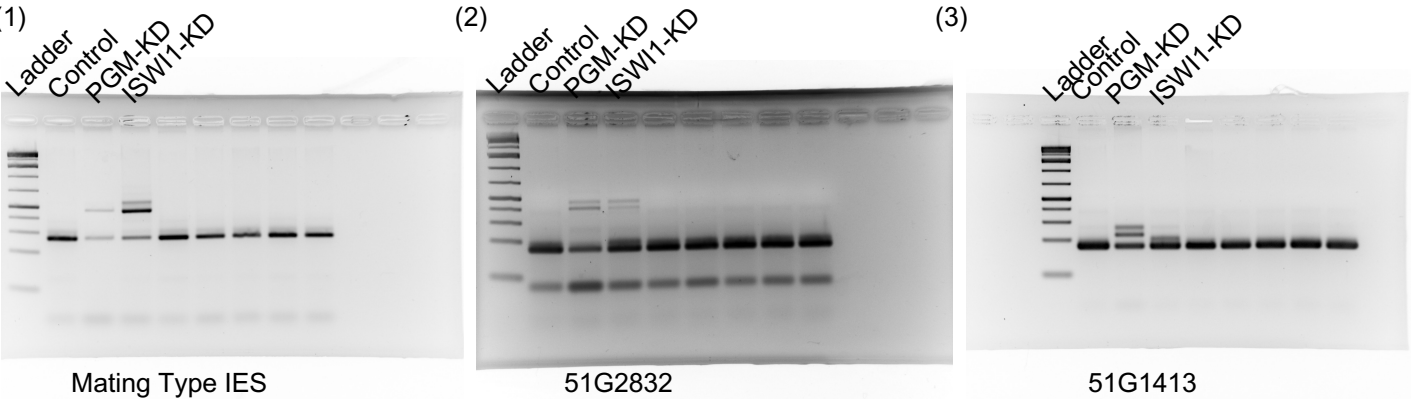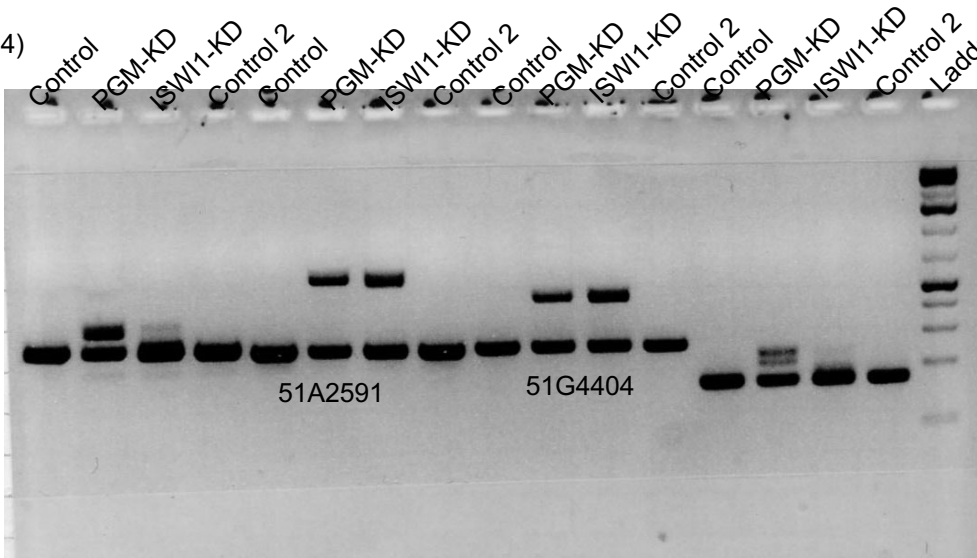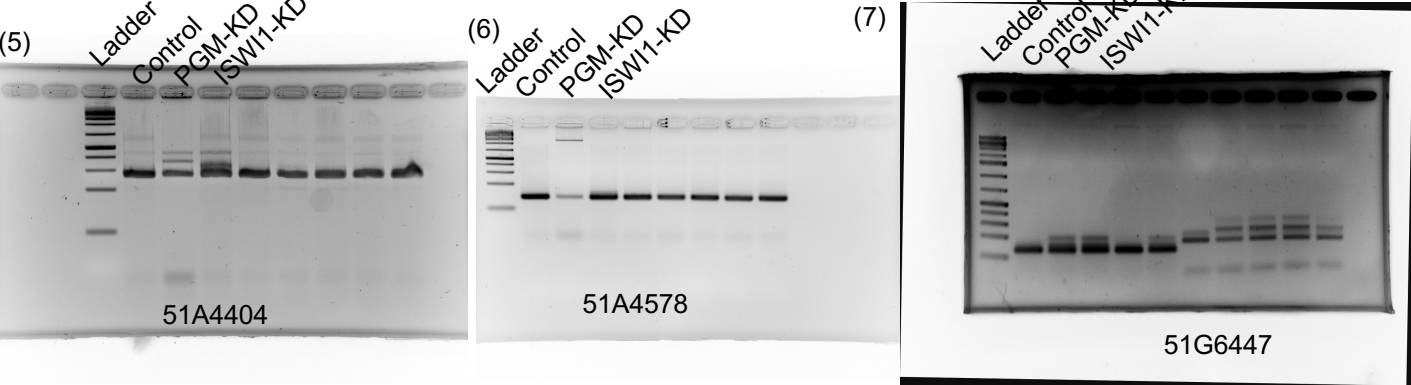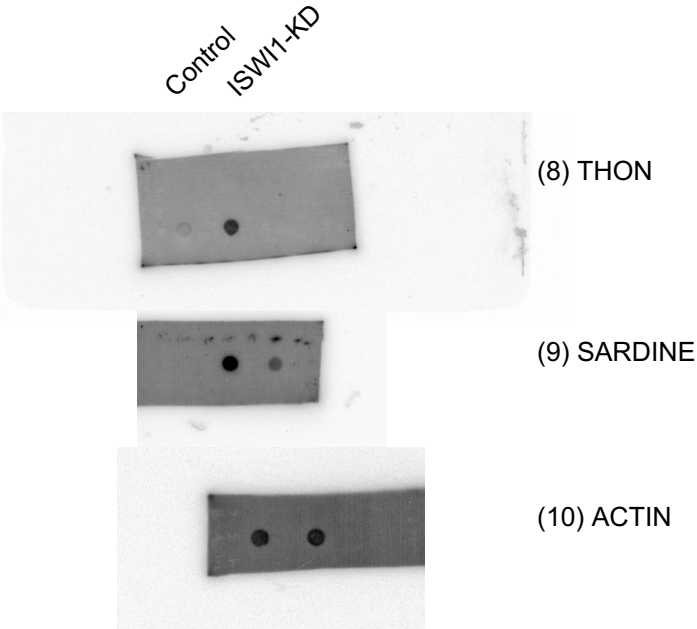

Supplement: Supplementary file 10 — Source Data for Figure 1 [file EMBJ-41-e111839-s012.zip › SourceData_Figure1/Source_Data_Figure1.pdf]

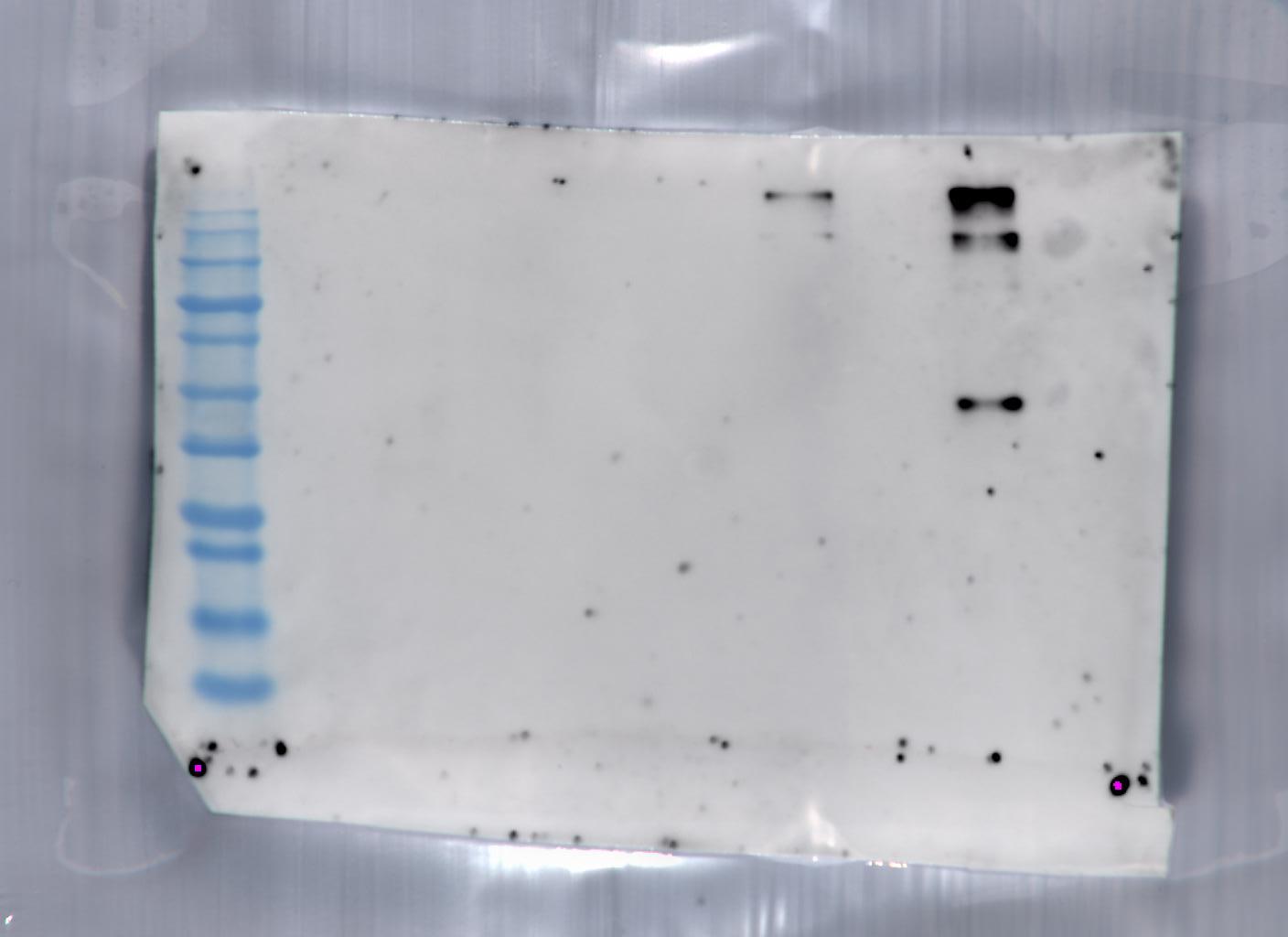

Supplement: Supplementary file 12 — Source Data for Figure 3 [file EMBJ-41-e111839-s003.zip › SourceData_Figure3/Figure3C_SourceData1.jpg]

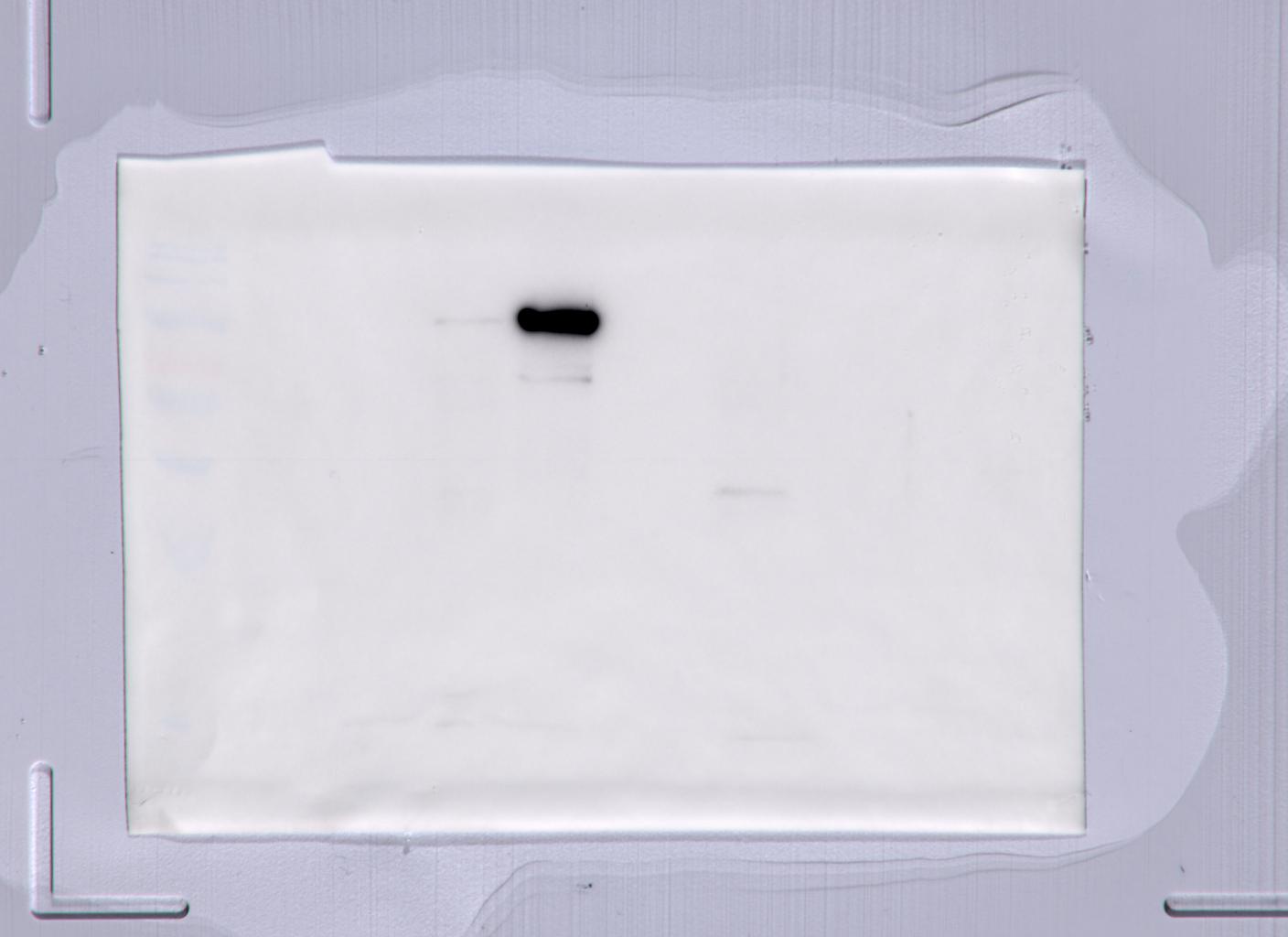

Supplement: Supplementary file 12 — Source Data for Figure 3 [file EMBJ-41-e111839-s003.zip › SourceData_Figure3/Figure3E_SourceData2.jpg]

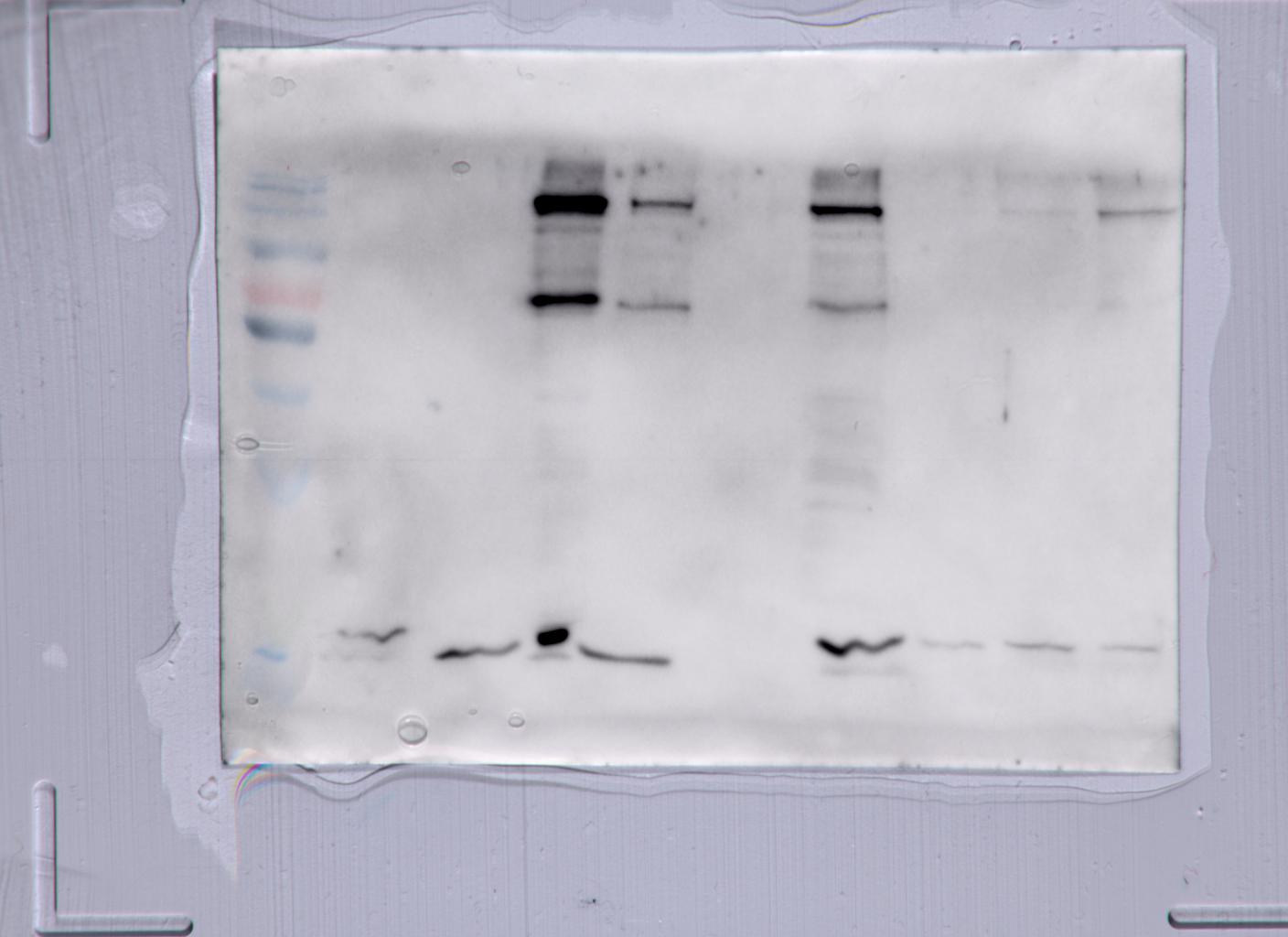

Supplement: Supplementary file 12 — Source Data for Figure 3 [file EMBJ-41-e111839-s003.zip › SourceData_Figure3/Figure3E_SourceData3.jpg]

Figure 3 Support Data1-3

(1)

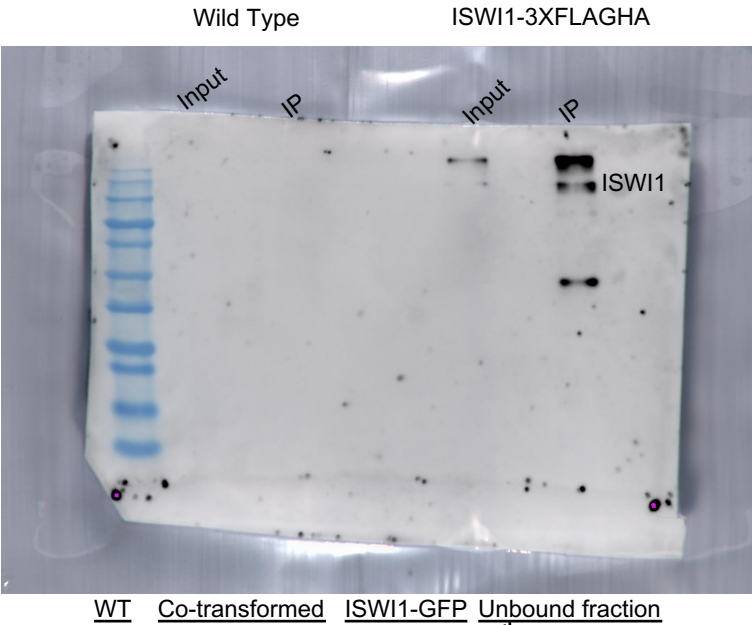

(2)

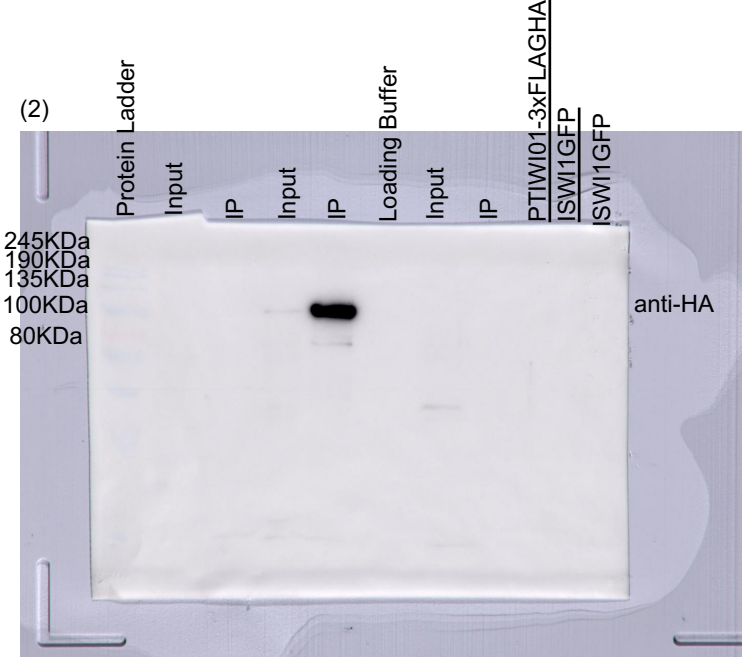

(3)

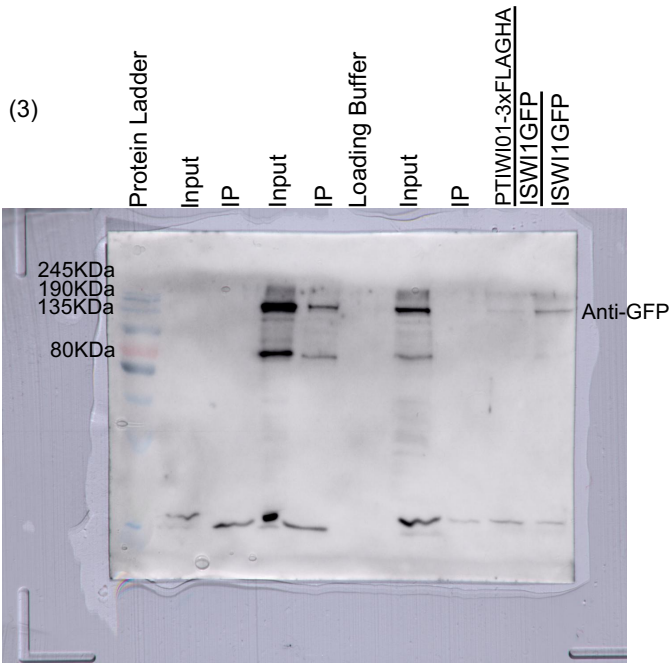

Supplement: Supplementary file 12 — Source Data for Figure 3 [file EMBJ-41-e111839-s003.zip › SourceData_Figure3/SourceData_Figure3.pdf]
