# Supplementary material for: Chromatin remodeling is required for sRNA‐guided DNA elimination in Paramecium
Source: EMBO J. 2022 Oct 11;41(22):e111839. doi: 10.15252/embj.2022111839 (PMC9670198; doi:10.15252/embj.2022111839)
Supplement: Supplementary file 13 — PDF+ [file EMBJ-41-e111839-s008.pdf]

Article

SOURCE DATA
TRANSPARENT PROCESS
OPEN ACCESS

THE EMBO JOURNAL

Chromatin remodeling is required for sRNA-guided DNA elimination in *Paramecium*

Aditi Singh[1,2,3], Xyrus X Maurer-Alcalá[1], Therese Solberg[1,2], Lilia Häußermann[3], Silvan Gisler[1], Michael Ignarski[1], Estienne C Swart[1,3,†] & Mariusz Nowacki[1,*]

Abstract

Small RNAs mediate the silencing of transposable elements and other genomic loci, increasing nucleosome density and preventing undesirable gene expression. The unicellular ciliate *Paramecium* is a model to study dynamic genome organization in eukaryotic cells, given its unique feature of nuclear dimorphism. Here, the formation of the somatic macronucleus during sexual reproduction requires eliminating thousands of transposon remnants (IESs) and transposable elements scattered throughout the germline micronuclear genome. The elimination process is guided by Piwi-associated small RNAs and leads to precise cleavage at IES boundaries. Here we show that IES recognition and precise excision are facilitated by recruiting ISWI1, a *Paramecium* homolog of the chromatin remodeler ISWI. ISWI1 knockdown substantially inhibits DNA elimination, quantitatively similar to development-specific sRNA gene knockdowns but with much greater aberrant IES excision at alternative boundaries. We also identify key development-specific sRNA biogenesis and transport proteins, Ptiwi01 and Ptiwi09, as ISWI1 cofactors in our co-immunoprecipitation studies. Nucleosome profiling indicates that increased nucleosome density correlates with the requirement for ISWI1 and other proteins necessary for IES excision. We propose that chromatin remodeling together with small RNAs is essential for efficient and precise DNA elimination in *Paramecium*.

Keywords chromatin remodeler; genome editing; nucleosomes; small RNAs; transposable elements
Subject Categories Chromatin, Transcription & Genomics; Microbiology, Virology & Host Pathogen Interaction
DOI 10.15252/embj.2022111839 | Received 8 June 2022 | Revised 14 September 2022 | Accepted 19 September 2022 | Published online 11 October 2022
The EMBO Journal (2022) 41: e111839

Introduction

Ciliates, such as *Paramecium tetraurelia* (class Oligohymenophora), provide excellent model systems to understand the dynamic genome organization in eukaryotic cells due to their unique feature of nuclear dimorphism. The formation of *Paramecium*'s somatic nucleus during sexual reproduction involves DNA endoreplication, DNA elimination, DNA repair, and transcription of genes that are specifically expressed when these processes occur (Chalker & Yao, 2011). Hence, the chromatin needs to be in a tightly controlled dynamic state. The germline micronuclear (MIC) genome contains regions that are removed during the development of the somatic macronuclear (MAC) genome (Beisson *et al*, 2010a) in a sophisticated process of genome reorganization, a natural form of genome editing. During this event, about 45,000 unique, noncoding Internal Eliminated Sequences (IES) are typically precisely excised (Arnaiz *et al*, 2012).

IES elimination is carried out by a catalytically active domesticated transposase PiggyMac (*PGM*; Baudry *et al*, 2009) in concert with catalytically inactive PGM homologs (Bischerour *et al*, 2018). Precise elimination of IESs is crucial for forming a functional somatic genome since these sequences would otherwise frequently interrupt exonic coding sequences. IESs have a distinctive, periodic size distribution and a weak end consensus sequence that probably reflects the preferences of the excision machinery (Baudry *et al*, 2009; Swart *et al*, 2014). However, the presence of consensus sequences is not enough for precise IES excision (Duret *et al*, 2008).

Currently, the proposed model for *Paramecium*'s IES excision involves two classes of small RNAs (scnRNAs and iesRNAs; Lepère *et al*, 2009; Sandoval *et al*, 2014b) that guide the process via indirectly comparing the maternal genome to the developing genome. These sRNAs are produced by Dicer-like proteins (Dcl2/3 and Dcl5, respectively; Lepère *et al*, 2009; Sandoval *et al*, 2014b) and Piwi proteins (Ptiwi01/09 and Ptiwi10/11, respectively; Bouhouche *et al*, 2011; Furrer *et al*, 2017b). However, as judged by the effects of gene knockdowns, most IESs in *P. tetraurelia* are efficiently excised independently of scnRNAs and iesRNAs (Sandoval *et al*, 2014b; Swart *et al*, 2017a). Other proteins also cooperate in IES excision, with substantial differences in the effects of knockdowns of their genes, suggesting it is far more complicated than can be explained by a single linear pathway (Nowacki *et al*, 2005; Kapusta *et al*, 2011; Dubois *et al*, 2012; Sandoval *et al*, 2014b; Maliszewska-Olejniczak *et al*, 2015; Swart *et al*, 2017a; Abello *et al*, 2020).

1 Institute of Cell Biology, University of Bern, Bern, Switzerland
2 Graduate School for Cellular and Biomedical Sciences, University of Bern, Bern, Switzerland
3 Max Planck Institute for Biology, Tubingen, Germany
*Corresponding author. Tel: +41 31 684 46 54; Fax: +41 31 684 44 31; E-mail: mariusz.nowacki@unibe.ch
†This author contributed equally to this work as senior author

© 2022 The Authors. Published under the terms of the CC BY 4.0 license.

Based on research in the ciliate *Tetrahymena* (class Oligohymenophora), one proposal for *PGM* recruitment and IES elimination suggests histone modifications mark IES boundaries, recruiting PGM for IES excision (Liu *et al*, 2007). Indeed, alteration of histone modifications, specifically H3K27me3 and H3K9me3, is associated with knockdowns of EZL1 and PTCAF1, affecting the excision of most IESs in *Paramecium* (Ignarski *et al*, 2014; Lhuillier-Akakpo *et al*, 2014b). In addition, both these modifications are scnRNA-dependent in *Paramecium* (Ignarski *et al*, 2014).

Nevertheless, fundamental differences exist between *Tetrahymena* and *Paramecium* IESs. Firstly, IES excision is predominantly precise in *Paramecium* and imprecise in *Tetrahymena* (Arnaiz *et al*, 2012; Coyne *et al*, 2012; Hamilton *et al*, 2016). Secondly, in contrast to *Tetrahymena*, the majority of IESs in *Paramecium* are scattered throughout the coding regions. Thirdly, the majority of *Paramecium* IESs are much shorter (median ~ 50 bp) than the size of a nucleosome (~146 bp; Arnaiz *et al*, 2012) or linker regions between MAC nucleosomes (several base pairs; Gnan *et al*, 2022). *Tetrahymena* IESs are much longer (hundreds of bp to kbp; Hamilton *et al*, 2016). Thus *Paramecium* DNA would often be expected to be wrapped around nucleosomes, making it difficult to access IESs for excision. Therefore, there is no particular expectation that the model for *Tetrahymena*, proposing the formation of heterochromatic DNA is necessary for IES excision, is applicable to the excision of most *Paramecium* IESs.

DNA elimination, carried out by *Paramecium*'s PGM, requires IES boundary accessibility. One way to do so would be through the action of ATP-dependent remodelers, such as SNF2-related proteins, that can restructure the chromatin providing access to DNA (Sadeh & Allis, 2011; Rando & Winston, 2012). Suggesting such activity, in *Tetrahymena*, an SNF2/brahma-related gene, TtBRG1 is known to be essential for nuclear development during conjugation (Fillingham *et al*, 2006). Numerous homologs of SNF2-related genes are conserved in *Paramecium tetraurelia* as well. Among these are homologs of ISWI, an SNF2-related, ATP-dependent chromatin remodeler (Pazin & Kadonaga, 1997). ISWI proteins form different complexes interacting with several conserved domains, with each complex modulating a discrete function (Dirscherl & Krebs, 2004). Although ISWI complexes have distinct functions, the general mechanism underlying their various roles is based on altering nucleosome spacing. By moving around nucleosomes, ISWI proteins help DNA-binding proteins access previously unavailable sites (Clapier & Cairns, 2009). To the best of our knowledge, there is currently no information regarding how nucleosomal positioning influences ciliate DNA excision. To this end, we studied the putative role of *Paramecium* ISWI, an SNF2-related protein (Pazin & Kadonaga, 1997), and its influence on both nucleosomes and DNA excision.

Results

We identified ISWI1 in a preliminary RNAi screening of genes with differential upregulation of expression during an autogamy (self-fertilization) time course (Arnaiz *et al*, 2010; Arnaiz & Sperling, 2011). *Paramecium tetraurelia* has five putative ISWI homologs with the characteristic SWI/SNF family ATPase core domain as well as SANT and SLIDE domains towards their C-termini (Fig 1A). Out of these, two pairs of paralogs arose from the well-characterized whole genome duplication (WGD, Fig 1B) events in *Paramecium* (Aury *et al*, 2006). Among these, the gene of the homolog characterized here, *ISWI1*, shows substantial differential upregulation during the macronuclear development, peaking during fragmentation and Dev1 stages of the autogamy time course (Fig 1C). In contrast, the other three paralogs; *ISWI2*, *ISWI3*, and *ISWI4* tend to be repressed during autogamy. The remaining *ISWI* homolog, *ISWI5*, also shows substantial differential expression, peaking during meiosis and fragmentation of the parental MAC before decreasing in abundance for the remainder of development (Fig 1C).

Knockdown of *ISWI1* affects cell survival and DNA elimination

We induced knockdown (KD) of *ISWI1* by feeding *Paramecium* with an *ISWI1*-specific sequence, triggering the cell's internal RNAi machinery (Fig EV1A). In a survival test of the post-autogamous progeny after *ISWI1*-KD over 3 days, 86% of the cells did not survive beyond the first day after cells were re-fed and allowed to resume vegetative division (Fig 1D). The remaining 14% of cells did not go through the usual rate of four vegetative divisions per day. In the control culture of *ND7*–KD (a gene required for exocytotic membrane fusion trichocyst discharge; Skouri & Cohen, 1997), the division rate of all the progeny remained unchanged. In the positive control of *PGM–KD*, 90% of the cells did not survive as expected. In contrast to *ISWI1–KD*, for *ISWI5*–KD, 90% of the cells showed no substantial difference in division rate or mortality compared to the control cells (Fig EV1B and C).

To test if the knockdown of *ISWI1* and *ISWI5* affect DNA elimination, we determined the retention status of germline-specific DNA elements in the newly developed MAC genome. We tested for IES retention from a well-characterized locus using PCR with IES-flanking primers (Appendix Table S1). For *ISWI1*–KD, most of the IESs we analyzed were retained (Fig 1F and Appendix Table S3). For *ISWI5*–KD, no retention of any of the IESs was observed (Fig EV1D). In *ISWI1–KD*, there was greater Sardine and Thon transposons retention, respectively, compared to the control *ND7*–KD (Fig 1E).

We also investigated the knockdown of other ISWI paralogs (*ISWI2*, *ISWI3*, and *ISWI4*; not upregulated during autogamy). In knockdown experiments for each of these paralogs, we did not observe growth defects or IES retention (Fig EV1E and F). To focus our investigations on genome reorganization, all further experiments were, therefore, carried out for *ISWI1* only.

ISWI1 is required for the complete excision of most IESs

To gain a genome-wide perspective on IES retention, we analyzed high-throughput sequencing of genomic DNA isolated from the developing macronucleus (anlagen) from *ISWI1*–KD cell cultures (two biological replicates). As a control, we used genomic DNA from the developing macronucleus after *ND7*–KD (also a pair of biological replicates). IES retention scores (IRSs) vary from 0.0 (complete IES excision) to 1.0 (complete failure of IES excision) upon knockdown. Approximately 35,000 (78%) IESs are sensitive to *ISWI1*–KD with a right-skewed retention score distribution (Fig 2A). IES retention scores of the biological replicates correlated well with each other (Pearson correlation coefficient: $r = 0.91$). Generally,

 © 2022 The Authors

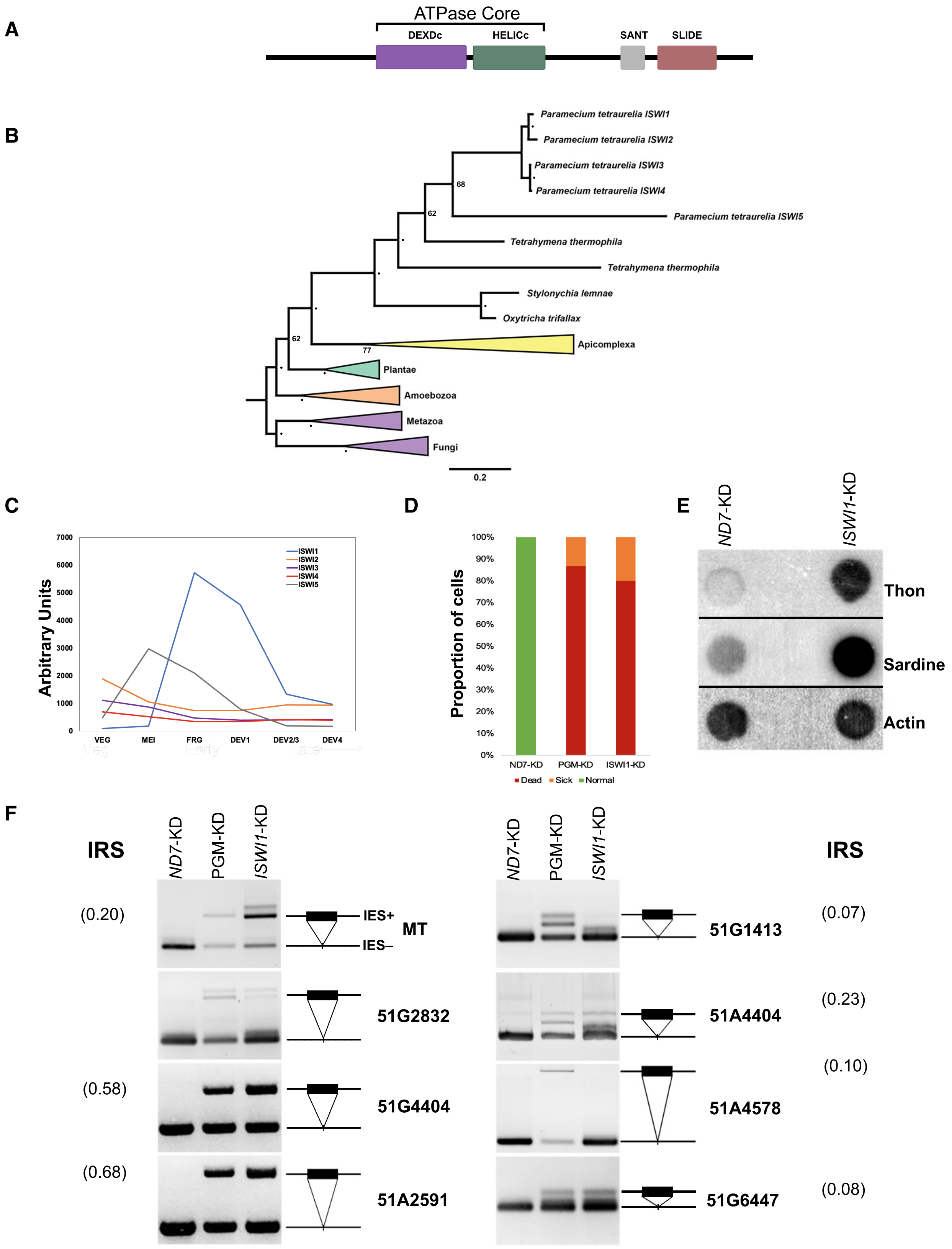
A
ATPase Core
DEXDc
HELICc
SANT
SLIDE
B
Paramecium tetraurelia ISWI1
Paramecium tetraurelia ISWI2
Paramecium tetraurelia ISWI3
Paramecium tetraurelia ISWI4
Paramecium tetraurelia ISWI5
Tetrahymena thermophila
Tetrahymena thermophila
Stylonychia lemnae
Oxytricha trifallax
Apicomplexa
Plantae
Amoebozoa
Metazoa
Fungi
68
62
62
77
0.2
C
Arbitrary Units
ISWI1
ISWI2
ISWI3
ISWI4
ISWI5
VEG
MEI
FRG
DEV1
DEV2/3
DEV4
D
Proportion of cells
ND7-KD
PGM-KD
ISWI1-KD
Dead
Sick
Normal
E
ND7-KD
ISWI1-KD
Thon
Sardine
Actin
F
IRS
ND7-KD
PGM-KD
ISWI1-KD
(0.20)
IES+
IES–
MT
51G2832
(0.58)
51G4404
(0.68)
51A2591
51G1413
(0.07)
51A4404
(0.23)
(0.10)
51A4578
51G6447
(0.08)

Figure 1.

Figure 1. Properties of ISWI1 and ISWI1-KD effects on DNA elimination.

A Predicted protein domains in ISWI1.
B Phylogenetic analysis of ISWI proteins in selected organisms. Node bootstrap values below ≥ 80 are indicated by '•' or are otherwise labeled.
C Gene expression profile (in arbitrary units) of ISWI genes based on published RNA-seq data (Arnaiz *et al*, 2010). Veg: cells undergoing vegetative division; Early: ~50% of cells with fragmented parental macronucleus (our early time point); Late: the majority of cells with a visible anlagen (our late timepoint).
D Survival test graph. Dead cells are represented in red, sick in orange, and normally dividing cells in green. *PGM*–KD is a positive control, and *ND7*–KD is a negative control.
E Dot blot analysis to check the effect of *ISWI1*-KD on transposon elimination. Probes against transposons Sardine and Thon were used, while a probe against Actin was used as a loading control.
F IES retention PCR (cropped inverted images). Four maternally-controlled IES and four non-maternally controlled IESs are shown. The IES+ band represents retained IES; the IES− band represents an excised IES; additional bands are likely PCR artifacts. IRS is IES retention Score for the IESs calculated after whole genome sequencing.

Source data are available online for this figure.

ISWI1–KD IES retention scores are modestly correlated with other known factors of excision machinery, correlating best with *DCL2/3/5*–KD ($r = 0.74$) and *NOWA1/2*–KD ($r = 0.72$; Fig EV2A). *ISWI1*–KD retention scores do not correlate as well with chromatin-related factors, *PTCAF1* ($r = 0.59$) and *EZL1* ($r = 0.52$).

As for most genes that influence IES excision, *ISWI1*–KD IES retention is length dependent (Fig EV2B). No periodicity of IES retention scores with respect to IES length is present. Similar to other gene knockdowns, IES sub-terminal base frequency changes relative to IES retention scores for *ISWI1*–KD, i.e., base frequencies are relatively constant for the shortest and most common IESs but differ considerably in relation to IES retention scores for longer IESs (Fig EV2C; Swart *et al*, 2014).

ISWI1-KD enhances excision of IESs at alternative boundaries

Excised IESs in *Paramecium* have a highly distinctive periodic length distribution (Fig 2D), proposed to reflect the periodicity of DNA and cooperation of transposase subunits during excision (Arnaiz *et al*, 2012). As can be seen in Fig 2D, the so-called "forbidden" second IES length peak (at ~40 bp; Arnaiz *et al*, 2012) is barely noticeable compared to the flanking IES length peaks. This was hypothesized as not being permitted by the biophysical constraints of DNA of this length, which prevents the two components of a conventional, domesticated PiggyBac transposase dimer from coming into the correct orientation needed for coordinated cleavage at both boundaries (Arnaiz *et al*, 2012). Since ISWI homologs are involved in nucleosome positioning in other organisms, we sought to determine if and how *ISWI1*–KD might impact IES excision precision.

First, we examined cryptic IESs, i.e., off-target IES-like sequences that are randomly excised at low levels throughout DNA, typically destined to become macronuclear during development (Duret *et al*, 2008; Swart *et al*, 2014). Such erroneous excision in *ISWI1*–KD was comparable to other knockdowns (Fig EV3C and D).

Next, we examined the excision of IESs at alternative boundaries. Natural excision of IESs using alternative boundaries occurs at low frequency, impacting ~16% of IESs in our negative control, *ND7*–KD (Fig 2B). In contrast, in *ISWI1*–KD, alternative boundary excision occurs at ~65% of IESs (supported by one or more mapped reads; Fig 2C). This is also substantially greater than for knockdowns of other genes necessary for IES excision, where the use of alternative IES boundaries is essentially the same as the control (Fig 2B). In general, though the amount of alternative IES excision for any given IES in *ISWI1*–KD is low (median 4.6%, mean 9.2%), it is substantially higher than that of other knockdowns (median 0%; mean 1.5–2.4%; Fig 2B).

The length distribution of alternatively excised IESs, irrespective of the knockdown, follows a similar periodic pattern to normal IESs, with smaller IESs more likely to result than larger ones (Fig 2D). Compared to normal IES excision, there is not as strong a preference for excision of the shortest IESs in alternative excision after *ISWI1*–KD.

Interestingly, there are substantially more alternatively excised IESs in *ISWI1*–KD in the second, "forbidden" length peak around 35 bp than conventional IESs (Fig 2D). We see a peak at this length of alternative excision events, regardless of whether they occurred internally versus externally (Fig EV3A and B). As for conventional IES excision, in other knockdowns, alternative IES excision in the forbidden length range was low (Fig 2D). Thus, enhanced alternative IES excision is a distinctive feature of *ISWI1*–KD. We also observe that most alternative excision events are close to the canonical IES boundaries, i.e., within 20 bp, or one or two turns of dsDNA (Fig 2E). In other words, *ISWI1*–KD leads to erroneous DNA excision at the next closest available sites.

ISWI1 protein localizes exclusively to the developing MAC

A C-terminal GFP fusion construct was made with *ISWI1* under the control of the putative *ISWI1* regulatory region and injected into *Paramecium* vegetative macronucleus. The transformed cell line was then cultured, and cells at different developmental stages (Fig 3A) were collected for confocal microscopy. When the early developing MACs (anlagen) were seen using DAPI staining, the GFP signal of the fusion protein also accumulated in the developing MAC and remained there throughout the late developmental stages (Fig 3B). The GFP signal was lost from the developing MAC after the developmental stages before karyogamy. Our observations suggest that the ISWI1 is expressed exclusively in the developing MAC at the time when genome reorganization takes place in *Paramecium*.

PTIWI01 and ISWI1 proteins interact *in vivo*

We sought to determine interacting partners of *Paramecium* ISWI1. First, we transformed *P. tetraurelia* cells with ISWI1 under its endogenous promoter and tagged it with a 3XFlagHA at its C-terminal. We then co-immunoprecipitated (IP) ISWI1 to analyze the associated proteins by label-free mass spectrometry. As a control, we performed the same experiment on wild-type cells where we did not expect to see any pulldown of proteins with the HA affinity

 © 2022 The Authors

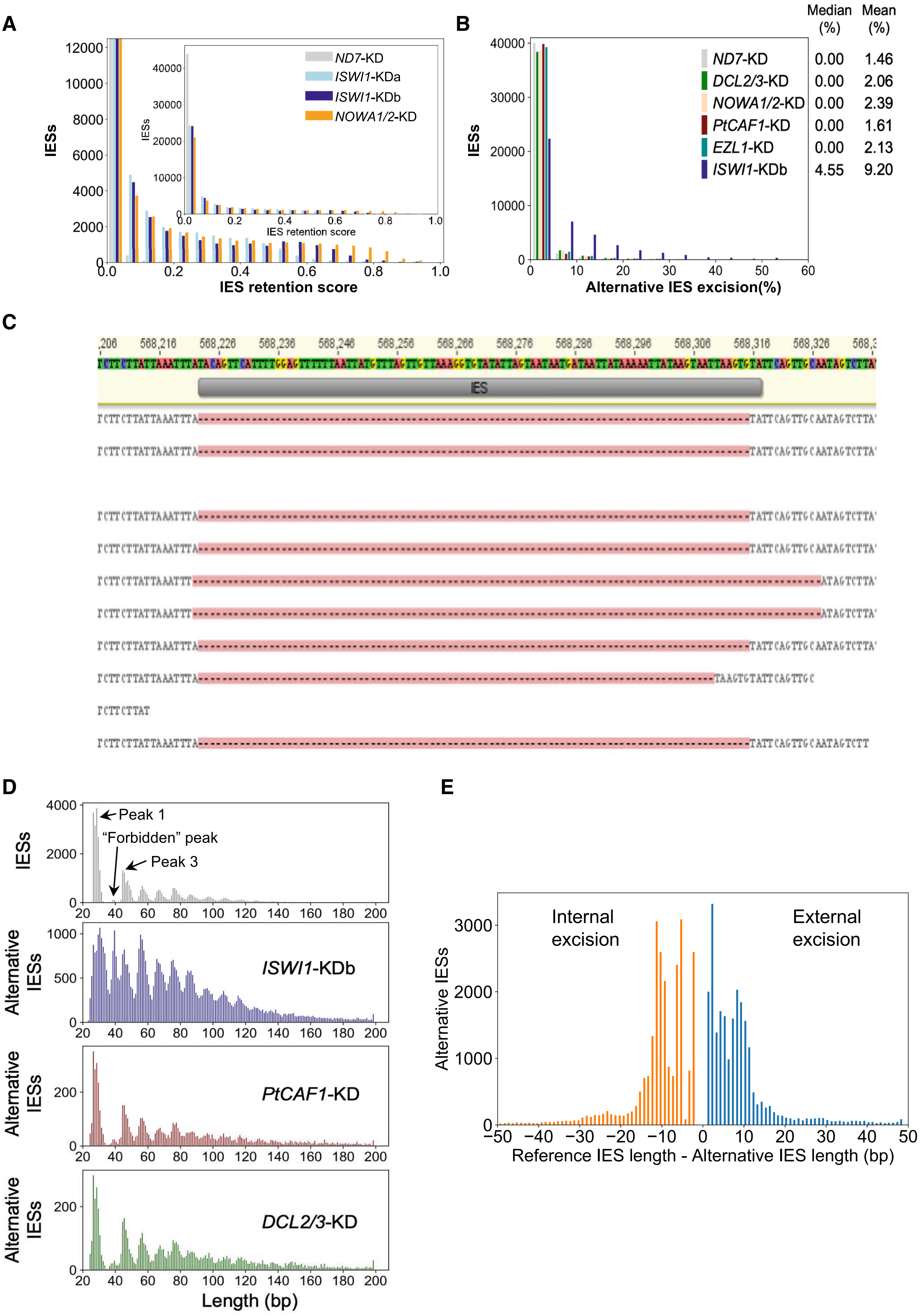
A
ND7-KD
ISWI1-KDa
ISWI1-KDb
NOWA1/2-KD
IESs
IES retention score
B
Median (%)
Mean (%)
ND7-KD 0.00 1.46
DCL2/3-KD 0.00 2.06
NOWA1/2-KD 0.00 2.39
PtCAF1-KD 0.00 1.61
EZL1-KD 0.00 2.13
ISWI1-KDb 4.55 9.20
IESs
Alternative IES excision(%)
C
IES
D
Peak 1
"Forbidden" peak
Peak 3
IESs
Alternative IESs
ISWI1-KDb
PtCAF1-KD
DCL2/3-KD
Length (bp)
E
Internal excision
External excision
Alternative IESs
Reference IES length - Alternative IES length (bp)

Figure 2.

Figure 2. Genome-wide analysis of IES excision upon ISWI1-KD.

A IES Retention Score (IRS) distributions for *ISWI1*–KD replicates and NOWA1/2-KD. ND7-KD was used as a negative control.

B Genome-wide analysis of alternative boundary excision in *ND7*-KD, *DCL2/3*-KD, *NOWA1/2*-KD, *EZL1*-KD, *PTCAF1*-KD, and *ISWI1*-KDb. Alternative excision (%) = 100*(alternative excised reads)/(alternatively + correctly excised reads).

C Reads mapped to an IES (IESPGM.PTET51.1.7.550914) showing both external (2 reads) and internal (1 read) alternatively excision; gaps opened in reads with excised IESs are indicated by dashes on a pink background.

D Length distribution of conventional IESs compared to alternatively excised IESs in knockdowns of ISWI1, PtCAF1, and DCL2/3.

E Difference in alternative IES lengths from the reference IES length.

Source data are available online for this figure.

matrix. The control cell samples and the cell samples transformed with ISWI1–3XFLAGHA were collected in two biological replicates during the developmental stage when ISWI1 localizes in the developing new MAC, as observed in Fig 3B. Before IP experiments, the samples were crosslinked with 1% PFA (see Materials and Methods).

We analyzed our IP samples by loading 1% of the total input and 20% of the IP fraction on an SDS gel. We detected a signal on a Western blot using an anti-HA antibody at the expected size of ~124 KDa (Fig 3C). The total IP samples were further analyzed using mass spectrometry (MS), where about 1,500 proteins were detected (Dataset EV1). Aside from proteins with peptides exclusively identified from cells expressing tagged ISWI1 protein, our analysis identified Ptiwi01 (or Ptiwi09, since most peptides from the mass-spectrometry analysis are shared between these almost identical proteins) as one of the proteins with the greatest fold enrichment in the ISWI1 IP (*P*-value: 0.0049; Figs 3D and EV4E, and Appendix Table S2).

We transformed *Paramecium* cells with 3XFLAGHA-tagged Ptiwi01 and GFP-tagged ISWI1 to test whether these proteins interact *in vivo*. IP samples were collected when ISWI1 localizes in the developing MAC. Cells were transformed with ISWI1–GFP as a negative control to check whether the GFP tag and HA affinity matrix could interact non-specifically. We performed the IP of the GFP-fused protein using the HA-affinity beads. GFP-fused ISWI1 (~150 KDa) was observed in the input but not in the IP (Fig 3E, lower panel). In addition, immunostaining was used to confirm the absence of HA signal in cells transformed with only ISWI1-GFP (Fig EV4F). Therefore, no cross-reactivity between the GFP and HA tags on their own was expected.

We observed no growth defects or IES retention in the transformed cells, either in single or in co-transformed cells (Fig EV4A–D). We succeeded in co-immunoprecipitating Ptiwi01 fused with 3XFLAGHA (expected size ~90 KDa) at the developmental stage when ISWI1 is expressed (Fig 3E, upper panel). IP samples were probed with an antibody against GFP, and a signal for ISWI1–GFP was detected at the expected size (~150 KDa, Fig 3E, lower panel). Our data suggest an interaction between ISWI1 and Ptiwi01, and most likely with Ptiwi09 (since they are 99% identical), in *Paramecium*. Since all our samples were crosslinked before the IP assays, we cannot exclude the possibility that this interaction might have been indirect via chromatin.

Nucleosomal densities increase with IES dependence on ISWI1 and other genes involved in *Paramecium* IES excision

We sought to determine whether nucleosome density changes occur around an IES during DNA elimination and whether this is ISWI1 dependent. For this, we isolated developing macronuclear DNA from *ND7/PGM*–KD and *ISWI1/PGM*-KD cultures either with or without Atlantis dsDNase treatment. Atlantis dsDNase cleaves phosphodiester bonds in double-stranded DNA and yields homogeneous populations of core nucleosomes. As *PGM* is a key component of the core endonuclease that cleaves IESs (Baudry *et al*, 2009; Arnaiz *et al*, 2012; Bischerour *et al*, 2018), we used *ND7/PGM*-KD as the control for our experiment, mapping the nucleosome density around IESs. A double knockdown of *ISWI1* with *PGM* is necessary to retain the majority of IESs to map the nucleosome density across them.

Given the constraint that a minimum of 9 bp of a read needs to match to an IES, and that some reads mapping to the flanking MDS regions may be derived from the parental MAC, it does not currently seem prudent to obtain accurate nucleosomal positioning for short IESs. We, therefore, examined a simpler measure of nucleosome densities for IESs: mapped nucleosome profiling (DNase-seq) reads, normalized by DNA-seq isolated from new MACs (Fig 4A–D).

In general, we observe that IESs, which are more strongly retained in any knockdown (e.g., *ISWI1*-KD IRS > 0.2), have higher nucleosome densities (Fig 4A and B). To rule out that this effect was not merely a consequence of more strongly retained IESs tending to be longer (e.g., Fig EV2B; Swart *et al*, 2014), we examined nucleosome density distributions of IESs of the same length, corresponding to the first IES length peak (26–31 bp). For these IESs, too, nucleosome densities are substantially higher for more strongly retained IESs (Fig 4C and D). Longer IESs (150–200 bp) show similar trends (Fig 4E and F), with higher nucleosome densities for more strongly retained IESs. Kolmogorov–Smirnov (KS) statistics between the distributions of IESs with IRS < 0.2 and IRS ≥ 0.2 vary between 0.33 and 0.38, with *P*-values < 1e-30 (Fig 4A–F).

ISWI1/PGM–KD alters the distribution of nucleosome densities compared to *ND7/PGM*–KD, for IESs in general and 26–31 bp IESs (Fig 4A–D; KS statistics between 0.048 and 0.089, with *P*-values < 1e-9). We also examined similar distributions for *NOWA1/2/PGM*–KD vs. *ND7/PGM*–KD (Fig EV5D–I), as both genes are required for the sRNA-mediated genome scanning (Nowacki *et al*, 2005), and their IES retention scores correlate more strongly with *ISWI1*–KD and *DCL2/3/5*–KD than *PTWI01/09*–KD. Though the coverage of DNase-seq was lower, as the number of nucleosomal reads mapping from the libraries was smaller, for a separate experiment with *ND7/PGM*–KD and *NOWA1/2/PGM*–KD, we also observed differences in distributions of nucleosomes for IESs, particularly those with *ISWI1*–KDb IRS ≥ 0.2 (KS statistics 0.17–0.19; *P*-values < 1e-7; Fig EV5D–I).

In summary, there appear to be differences in nucleosome density distributions between both *ISWI1/PGM*–KD and *ND7/PGM*–KD, and *NOWA1/2/PGM*–KD and *EV/PGM*–KD. However, these are much less pronounced than the difference in nucleosome density

 © 2022 The Authors

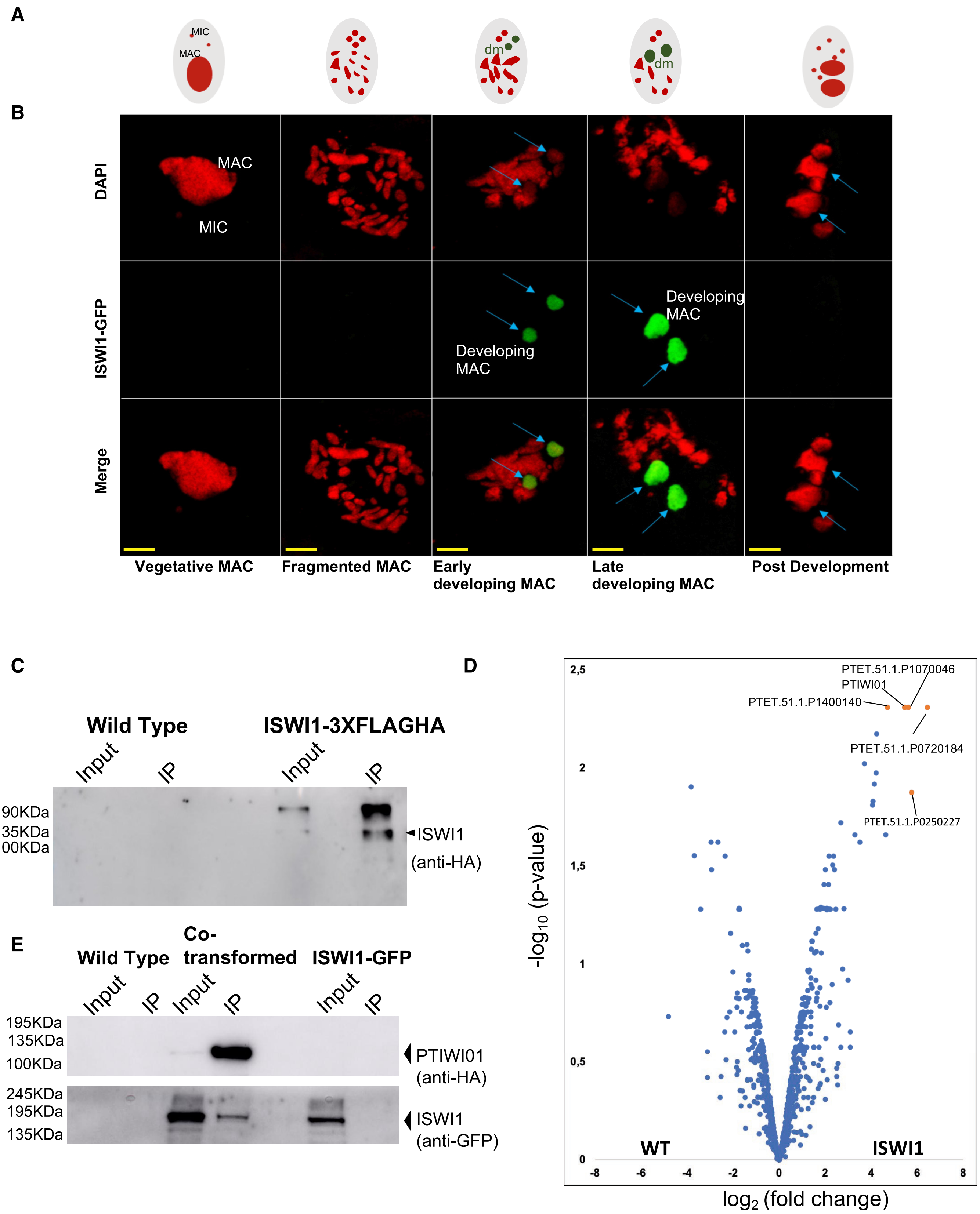
A
MIC
MAC
dm
dm
B
DAPI
ISWI1-GFP
Merge
MAC
MIC
Developing MAC
Developing MAC
Vegetative MAC
Fragmented MAC
Early developing MAC
Late developing MAC
Post Development
C
Wild Type
ISWI1-3XFLAGHA
Input
IP
Input
IP
190KDa
135KDa
100KDa
ISWI1 (anti-HA)
D
PTET.51.1.P1070046
PTIWI01
PTET.51.1.P1400140
PTET.51.1.P0720184
PTET.51.1.P0250227
-log10 (p-value)
log2 (fold change)
WT
ISWI1
E
Wild Type
Co-transformed
ISWI1-GFP
Input
IP
Input
IP
Input
IP
195KDa
135KDa
100KDa
245KDa
195KDa
135KDa
PTIWI01 (anti-HA)
ISWI1 (anti-GFP)

Figure 3.

Figure 3. Localization, Co-immunoprecipitation, and mass spectrometry analysis.

A Schematic drawing of the life cycle stages of *Paramecium tetraurelia*. MIC and parental MAC are represented in red, representing the DAPI signal, and developing MAC (dm) is represented in green until fully developed, representing the GFP signal.

B ISWI1-tagged C-terminally with GFP localizes in the developing MAC as soon as developing new MACs (panel Early Development) become visible and remain there throughout late MAC development (panel Late Development). Red: DAPI, Green: ISWI1–GFP. Blue arrows identify developing MAC; scale bar 10 μm.

C Western blot analysis using anti-HA antibody after coimmunoprecipitation of ISWI1-3XFlagHA fusion protein. Non-transformed cells (WT) of the same strain were used as the negative control. 1% of the total lysate was loaded as Input, and 20% of co-immunoprecipitated samples were loaded on 12% SDS gel.

D Volcano plot illustrating the distribution of proteins identified in label-free MS in WT Vs. ISWI1-3XFlagHA. Significantly abundant proteins (fold change ≥ 4) are highlighted in orange.

E Western blot analysis using anti-HA and anti-GFP antibodies after coimmunoprecipitation of Ptiwi01-3XFlagHA fusion protein co-transformed with ISWI1-GFP. Non-transformed cells (WT) of the same strain and ISWI1-GFP fusion protein transformation were used as negative controls. 1% of the total lysate was loaded as Input, and 20% of co-immunoprecipitated samples were loaded on 10% SDS gel.

Source data are available online for this figure.

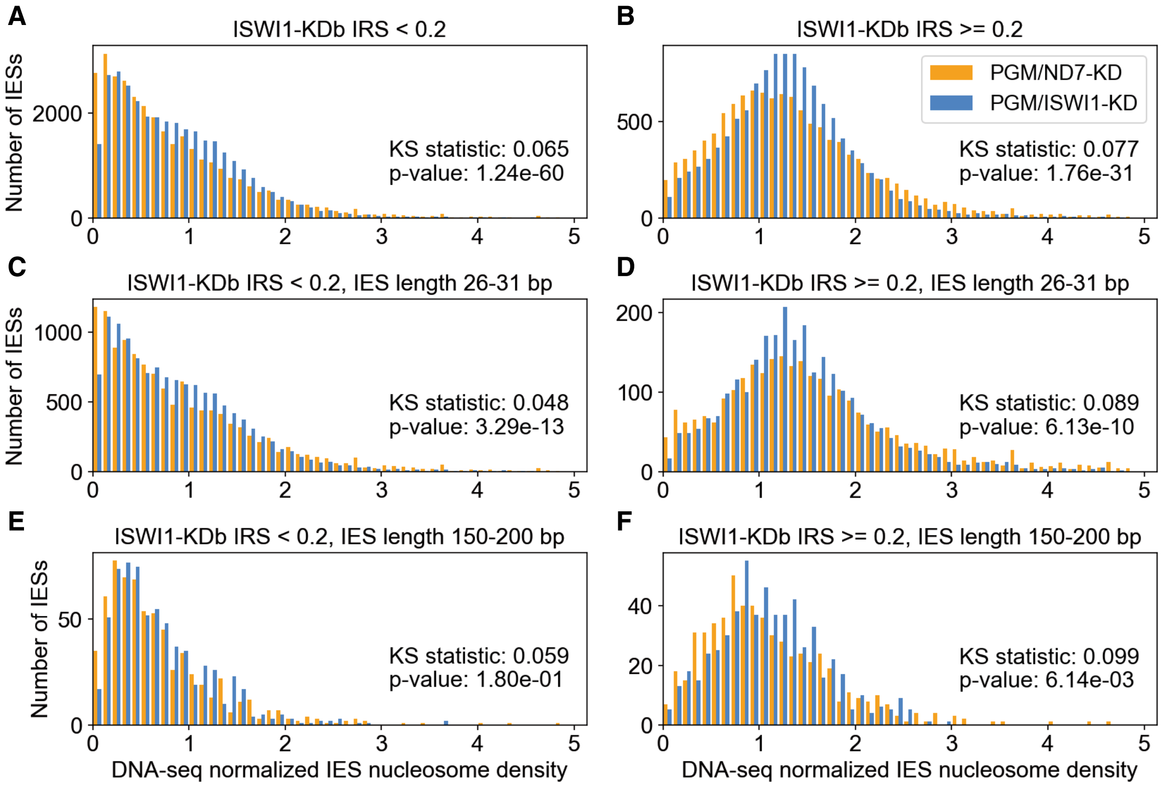

Figure 4. Nucleosome density increases with IES retention in ISWI1-KD.

A, B Nucleosome density histograms for IESs weakly (IRS < 0.2) or more strongly retained in *ISWI1*-Kdb (IRS ≥ 0.2). Kolmogorov–Smirnov statistics and their *P*-values are provided.

C–F Histograms as in (A&B), including additional length constraints, corresponding to the first IES length peak (26–31 bp; C and D) or the first non-periodic length IESs (150–200 bp; E and F).

distributions between IESs that are more weakly and more strongly retained in knockdowns like *ISWI1*–KD.

Discussion

Paramecium depends on efficient and accurate whole genome reorganization to produce a functional somatic nucleus during sexual reproduction. The excision of numerous IESs requires scnRNAs for their excision. Identification of additional proteins required for the excision of IESs (Arambasic *et al*, 2014; Data ref: Lhuillier-Akakpo *et al*, 2014a; Wasmuth & Lima, 2017) suggests additional or alternative mechanisms beyond those envisaged in earlier models of RNA scanning and heterochromatin formation contributing to IES targeting and excision.

In this study, we have identified a homolog of ISWI, an ATP-dependent chromatin remodeler, that is required for the precise elimination of IESs. ISWI proteins are highly conserved

 © 2022 The Authors

ATP-dependent chromatin remodelers (Corona *et al*, 1999) that regulate several biological processes (Yadon & Tsukiyama, 2011), and now, as we have shown, also in genome editing in *Paramecium*. *Paramecium*'s ISWI1 is exclusively present in the developing macronucleus (Fig 3A) when the molecules responsible for genome reorganization cooperate to eliminate DNA. We also show that, in *Paramecium*, ISWI1 can interact with PIWI proteins (Fig 3E) that are known to guide genome reorganization in ciliates in an sRNA-dependent manner (Bouhouche *et al*, 2011; Furrer *et al*, 2017b). Our data, therefore, suggest that the shifting action of ISWI1 occurs in conjunction with an sRNA–Piwicomplex that guides subsequent precise excision.

Histone modification and heterochromatin formation are proposed to be a prerequisite for programmed DNA elimination in ciliates. The most evidence in support of this has been obtained for *Tetrahymena thermophila* (Liu *et al*, 2007; Xu *et al*, 2021). A similar model was proposed for IES excision in *Paramecium* (Coyne *et al*, 2012). It has been shown that histone modifications, in particular H3K27me3 and H3K9me3, are required for targeting the excision of at least a subset of IESs (Ignarski *et al*, 2014; Data ref: Lhuillier-Akakpo *et al*, 2014a). Indeed, the knockdown of EZL1, a histone methyltransferase (Frapporti *et al*, 2019), affects the excision of the majority of IESs. Since heterochromatin regions generally spread across several kilobases in the genomes of other organisms (Margueron & Reinberg, 2011; Huang *et al*, 2012), it was suggested that in *Paramecium*, H3K27me3 and H3K9me3 marks are placed locally (Lhuillier-Akakpo *et al*, 2014b). Although it was recently shown that the transposable elements are enriched with nucleosomes bearing these modifications (Frapporti *et al*, 2019), currently, there is no published information on H3K27me3 or H3K9me3 nucleosome association with IESs. Moreover, these modifications are not limited to the developing macronucleus and are also present in the fragments of the parental macronucleus (Ignarski *et al*, 2014; Lhuillier-Akakpo *et al*, 2014b; Frapporti *et al*, 2019). The inhibition of IES excision and the resultant cell lethality due to *EZL1*–KD and/or *PTCAF1*–KD may arise due to alteration in gene expression and failure to repress transposable elements by the PRC complex that also interacts with Ptiwi01/09 proteins (Miró-Pina *et al*, 2022). Thus, further experiments will be necessary to disentangle possible indirect effects of these knockdowns from direct ones.

ISWI1–KD IES retention correlates better with *DCL2/3/5*–KD than with *DCL2/3*–KD or *DLC5*–KD (Fig EV2A), suggesting ISWI1 is necessary for excision of IESs requiring either scnRNAs or iesRNAs. In addition, we also observed an interaction between Ptiwi01 (or Ptiwi09) and ISWI1 *in vivo* in our co-immunoprecipitation assay, though this may be an indirect action with chromatin intervening (Fig 3E). We also observed Ptiwi11 in our mass spectrometry analysis (Appendix Table S2). Taken together with a stronger correlation between *DCL2/3/5*-KD and *ISWI1*–KD, we suggest ISWI1 also cooperates with iesRNAs in targeting IESs.

IESs most sensitive to *ISWI1*–KD and other knockdowns, like *DCL2/3/5*–KD, are substantially more nucleosome rich (Fig 4C–H). Like *ISWI1/PGM*–KD, *NOWA1/2/PGM*–KD alters the distribution of nucleosome densities across IESs. However, this is certainly more subtle than the large differences in these densities observed between weaker and more strongly retained IESs upon *ISWI1*–KD (compare Figs 4C–H and EV5D–I) and other genes we examined that are involved in genome reorganization. A plausible explanation could be that local nucleosome density changes are required to govern accessibility and possibly activating the endonuclease for DNA elimination. A similar explanation has been proposed for V(D)J recombination, where nucleosome location and occupancy changes were observed to regulate DNA recombination (Pulivarthy *et al*, 2016).

In the future, detailed DNase-seq experiments with variable nuclease digestion conditions and deeper sequencing may be able to obtain greater resolution and examination of dynamics. Furthermore, it will be necessary to conduct additional experiments to resolve the possible contributions of non-nucleosomal proteins to protecting DNA from DNase digestion. Nevertheless, as nucleosomal proteins are the most abundant nuclear DNA-binding proteins, we believe they are the largest contributors to the differences in DNase-seq read distributions we observed, hence why we refer to them as nucleosome density distributions.

Recent research into *Paramecium* MAC chromatin has revealed notable differences from other eukaryotes, including the ciliate *Tetrahymena*, including the absence of linker histones in *Paramecium* (Drews *et al*, 2022; Gnan *et al*, 2022). In particular, *Paramecium* has extremely average short internucleosomal distances (~151 bp). This would correspond to tiny linker sequences of several bases, rather than tens of bases in other eukaryotes, including *Tetrahymena* (Drews *et al*, 2022; Gnan *et al*, 2022). Thus, we expect *Tetrahymena* IES excision constraints to differ fundamentally from *Paramecium's*.

Uniquely among *Paramecium* proteins involved in IES excision investigated thus far, *ISWI1* gene silencing leads to elevated alternative IES excision (Fig 2B), suggesting that the endonuclease complex is not always able to correctly target the boundaries of an IES in the absence of ISWI1. The commonly accepted mechanism underlying ISWI function is that it controls the length of linker DNA and the chromatin architecture by altering nucleosome spacing (Xiao *et al*, 2001; Corona *et al*, 2007; Bartholomew, 2014). Global nucleosome density changes are known to occur across genomes during cell lineage commitment as an additional regulatory mechanism (Erdel *et al*, 2011; Li *et al*, 2012).

We propose that the presence of nucleosomes on, or partially overlapping, an IES may be crucial for its targeting and accessibility to the excision machinery (Fig 5). In contrast to the current "naked" DNA model for IES excision (Fig 5A), we propose a "clothed" DNA model with nucleosomes present. Crucially, in our model, IES boundaries need to be accessible to their excesses. We propose that "forbidden" length DNA is cut when nucleosomes have not been displaced from IES boundaries by ISWI1, as happens with *ISWI1*–KD (Fig 2D). In the absence of the usual required nucleosomal shift, IESs can be excised at alternative TA boundaries, though they are still most frequently cut at the conventional boundaries (Fig 5B and C). In other words, *ISWI1*–KD assists in properly positioning nucleosomes around an IES, preventing alternative excision errors.

In Fig 5, we do not indicate the involvement of any histone modifications in *Paramecium* IES excision. Until more detailed analyses can be performed, showing the exact positioning of specific histone modifications in relation to IESs, we would prefer to avoid speculating about their role. On the other hand, it may also be possible for an alternative mechanism for IES targeting that does not invoke such modifications. Instead, it might also be possible that longer RNA transcripts across IESs promote binding of scnRNAs/iesRNAs,

© 2022 The Authors

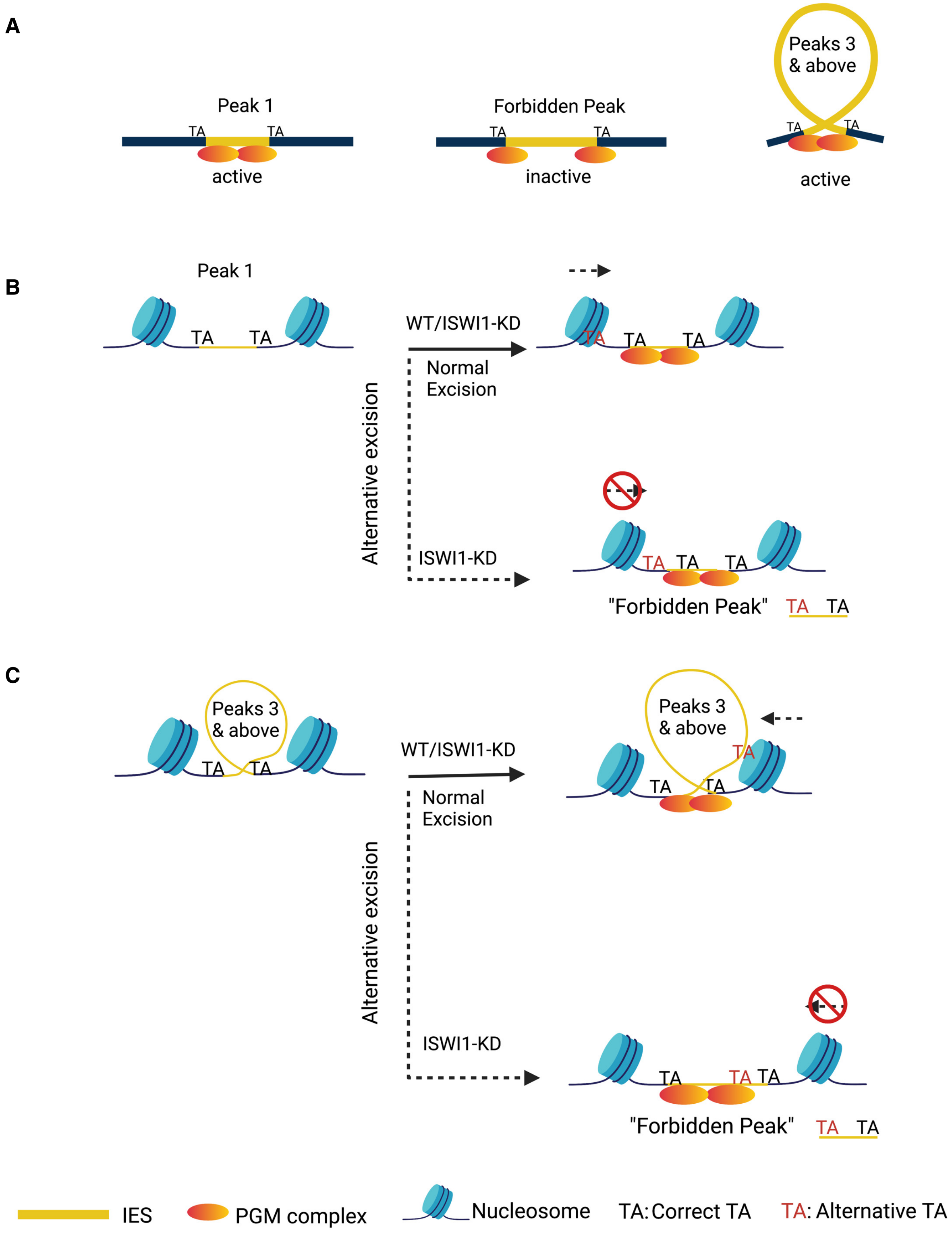

Figure 5. Assembly of active PiggyMac (PGM) excision complex on IESs.

A–C (A) "Naked" model proposed by Arnaiz *et al*, 2012; (B and C) Revised "clothed" model, which accounts for accessibility of IES boundaries in the presence of nucleosomes. If nucleosomes are not properly positioned, IESs can be cut at alternative boundaries, leading to IES accumulation of the "forbidden peak" length. Image created with BioRender.com.

 © 2022 The Authors

and more direct recruitment of the IES excisases. In any event, more detailed experiments will be necessary to examine nucleosomal properties, including positioning and modifications, and how they might influence IES excision.

Taken together, our investigations provide evidence of an interplay between chromatin remodeling and sRNA-complexes during *Paramecium* genome development. Typically chromatin remodelers do not operate in isolation in other organisms but as multi-component complexes, performing a range of sophisticated functions. In the future, it would be necessary to closely examine the mechanistic details of the interplay of ISWI1 with sRNAs and other proteins in *Paramecium* and how they are involved in massive, accurate genome editing.

Materials and Methods

Paramecium cultivation

Mating type 7 of *Paramecium* strain 51 was used in different experiments. Cells were cultured in Wheat Grass Powder (WGP; Pines International, Lawrence, KS) medium bacterized either with non-virulent *Klebsiella pneumoniae* or with *Escherichia coli*, strain HT115 and supplemented with 0.8 mg/l of β-sitosterol (567152, Calbiochem). Cells were either cultured at 27°C or at 18°C as per requirement. Clonal cell lines of *Paramecium* transformed with recombinant genes were maintained at 18°C as previously described (Beisson *et al*, 2010b).

Knockdown experiments, survival test, and IES retention PCR

The silencing (gene knockdown) construct of *ISWI1* (Genbank accession: XM_001431568, XM_001431569) was made by cloning a 704 bp construct from its C-terminal and cloned into an L4440 plasmid (using GGGTCTCACCTAAGATGAACG and TCACTTTCTTAACAGACTCAGATCC). *ISWI2* (Genbank accession: XM_001447087.1) a 584 bp long region (using GGAGGAGCGTTAAGAACAA, CACAAGAGATCTTCCCATAG) was used for generating the silencing construct. For *ISWI3* (Genbank accession: XM_001442140), using CTT AGCTAGTCATCTCTTT and CTTTTCATAAGCATCCTTG oligonucleotides, a 500 bp long region was cloned, and for *ISWI4* (Genbank accession: XM_001446844.1) a 394 bp long region was cloned (using CAATTGCTAATCATCATTTC, GAGAGTTTTGGATTTAACG) for the knockdown experiments. For *ISWI5* (Genbank accession: XM_001432642), the silencing construct was made by cloning an 1,106 bp long fragment into an L4440 plasmid (using ATGAGTGAAAGTGAAGATGAG and AGATTTCGTCCTTCTTAACAT). The plasmids were then transformed into HT1115 (DE3) *E. coli* strain.

Cells were seeded into the silencing medium at a density of 100 cells/ml, and silencing was carried out according to a previously described protocol (Beisson *et al*, 2010c). After the cells finished autogamy, 30 post-autogamous cells were transferred individually to threewell glass slides containing the medium bacterized with avirulent *K. pneumoniae* for the survival test. Cells were monitored for 3 days (approximately 12 divisions) and categorized into three groups according to their observed phenotype. In parallel, a 100 ml culture was harvested for DNA extraction using GeneElute–Mammalian Genomic DNA Miniprep Kit (Sigma-Aldrich). PCRs were done on different genomic regions flanking an IES (Appendix Table S1).

In regards to co-silencing performed to analyze nucleosomal densities, the distribution of retention scores in *PGM/ND7*–KD is shifted and skewed to the left (lower IES retention) compared to the reference *PGM*–KD data sets (Arnaiz *et al*, 2012; Swart *et al*, 2014), whereas, the IRS of *ISWI1/PGM*-KD is more similar to the knockdown expected for *PGM*–KD (Fig EV5A). Previous experiments have shown that weakened IES retention due to dilution of gene knockdown can occur in *Paramecium* due to gene co-silencing (Bischerour *et al*, 2018). The weaker silencing effect can be explained by the dilution of the *PGM* silencing medium with the *ND7* silencing medium. This was done to ensure that the RNAi effects from the *PGM/ND7* and the *ISWI1/PGM* knockdowns would be directly comparable.

Related to this, for *NOWA1/2/PGM* silencing, *NOWA1/2*-KD also minimizes potential dilution effects since one silencing construct silences both genes (Nowacki *et al*, 2005), whereas *PTIWI01/09*–KD requires two silencing constructs in addition to the necessary *PGM* silencing construct.

Dot blot

Dot blot assays were conducted following standard protocols (Brown, 2001). Briefly, 3 μg of DNA from post-autogamous cultures were blotted onto a nylon membrane (Hybond N+XL membrane, Amersham). Probes specific to Sardine and Thon transposons and actin (first 240 bp of the gene) labeled with α-32P dATP (3,000 Ci/mmol) using RadPrime DNA Labeling System (Invitrogen) were used. The signal was quantified with ImageJ 1.48e.

Northern blot

Ten microgram of RNA were run in a 1.2% agarose denaturing gel and transferred to a nylon membrane (Hybond N+XL membrane, Amersham) by capillary blotting. After transfer, the membrane was crosslinked twice with UV (120,000 μJ/cm^2). Specific probes labeled with α-32P dATP (3,000 Ci/mmol) using RadPrime DNA Labeling System (Invitrogen) for ISWI1, ISWI5, and rRNA were used for hybridization. Membranes were screened using the Typhoon Imaging system (GE Healthcare).

GFP tagging, microinjection, and GFP localization experiment

A set of specific *ISWI1* specific primers (5′-GTA GAA TCC TAT TGA TAG GAG GAG-3′ and 5′-TGG CTC TAA GAA ATT CAT TTA T-3′) were used for the amplification of full gene including 227 bp upstream and 62 bp downstream of the coding region. *ISWI1* was tagged with GFP on its C-terminus. The construct was linearized using the NaeI restriction enzyme (R0190S, New England Biolabs) and injected into the macronucleus of the vegetative cells as previously described (Beisson *et al*, 2010d). Cells positive for GFP expression were collected during different stages of autogamy and either stored with 70% ethanol at −20°C or directly fixed with 2% PFA in PHEM and then washed in 5% BSA with 0.1% Triton X-100. Cells were then counterstained with DAPI (4,6-diamidino-2-2phenylindole) in 5% BSA with 0.1% Triton X-100 and mounted with Prolong Gold Antifade mounting medium (Invitrogen). Images

© 2022 The Authors

were then acquired with Olympus Fluoview FV1000 confocal microscope system with PLAPON 60× O SC NA 1.40. Images were analyzed and given pseudo-color on Imaris software.

Immunofluorescence analysis

Immunostaining of cells transformed with ISWI1-GFP was done after the cells were fixed in 70% ethanol at −20°C. Cells were first washed in 1× PBS pH7.4 twice for 5 min to remove any traces of ethanol. Cells were then permeabilized with 1% Triton X-100 in Pipes–Hepes–EGTA–MgCl2 (PHEM) buffer for 20 min at room temperature. Afterward, cells were fixed in 2% paraformaldehyde for 10 min and washed with 1× PBS for 5 min. Cells were then blocked in 3% BSA in TBSTEM buffer for 1 h at room temperature. Primary antibody incubation was done at 4°C overnight using mouse anti-HA (sc-7,392, Santa Cruz) with a 1:50 dilution factor. After washing the cells three times for 10 min in 3% BSA in TBSTEM, cells were incubated in goat anti-mouse Alexa-594 (dilution 1:200, BLD–405326, Biozol) for 1 h in dark conditions. The cells were further washed three times for 10 min in 3% BSA in TBSTEM. In the last wash, DAPI was added to the BSA, and cells were incubated for 5 min. The cells were then mounted with Prolong® Gold Antifade mounting medium (Life Technologies), and sealed with a coverslip. Images were acquired on TCS SP8 with a 63×/1.40 oil objective, zoom factor 3 and step size of 1.0. Images were analyzed using Fiji with maximum intensity projection.

Co-immunoprecipitation assay

ISWI1 specific primers (5′-GTA GAA TCC TAT TGA TAG GAG GAG-3′ and 5′-TGG CTC TAA GAA ATT CAT TTA T-3′) were used for the amplification of the full gene with regulatory regions. The gene was tagged with 3XFLAGHA at its C-terminal. 4.5×10^5 cells were harvested and crosslinked with 1% Paraformaldehyde by incubating for 10 min (min) at room temperature. Cells were then quenched using 100 µl of 1.25 M Glycine and incubated at room temperature for 5 min. Cells were washed once with PBS for 2 min at 500 *g*. Further steps were carried out on ice or at 4°C. Two milliliter of lysis buffer (50 mM Tris pH 8.0, 150 mM NaCl, 5 mM $MgCl_2$, 1%Triton X100, 1× Protease inhibitor (Roche,11836170001), 10% glycerol) was added and the cells were sonicated (Branson Digital Sonifier) with 55% amplitude for 15 s. The lysate was then centrifuged for 30 min at 13,000 *g* or until the lysate was clear. Fifty microliter of bead slurry (HA High-Affinity Matrix,11815016001, clone 3F10, Roche) was used per IP sample and was washed thrice by centrifuging for 2 min at 500 *g*. After washing the beads, 1 ml of the lysate was mixed with the beads and incubated overnight with agitation at 4°C. After the incubation, the beads were washed five times with the IP buffer (10 mM Tris pH8.0, 150 mM NaCl, 0.01% NP-40, 1 mM $MgCl_2$, 1× Protease inhibitor (Roche,11836170001), 5% Glycerol) for 2 min at 500 *g*. NP-40 was added freshly to the buffer. Proteins were then eluted by adding 50 µl of the 2× loading buffer (10% SDS, 0.25 M Tris pH 6.8, 50% Glycerol, 0.2 M DTT, 0.25% Bromophenol blue).

For co-transformation with ISWI1-GFP, PTIWI01 with primers in its regulatory regions was (CATTTTTAAGAGATTTCAATAAAA-CAATTATCC and GTGCTTTGAAAATCAATGAAAATCA) amplified, and 3XFLAGHA was fused at its N-terminal. After linearisation with NaeI, both constructs were mixed in equal proportions for microinjection. Co-immunoprecipitation assay was performed as explained above with a slight modification. Sonication was done with 52% amplitude for 20 s using MS72 tip on Bandelin Sonopulse.

Mass spectrometry analyses

Mass spectrometry data processing and statistics were provided by the Proteomics & Mass Spectrometry Core Facility (PMSCF), University of Bern. The mass spectrometry proteomics data have been deposited to the ProteomeXchange Consortium (Deutsch *et al*, 2020) via the PRIDE (Perez-Riverol *et al*, 2019) partner repository with the dataset identifier PXD027206.

Differential expression tests were performed for proteins detected in the control and ISWI1 pulldown groups by applying the empirical Bayes moderated *t*-test (Kammers *et al*, 2015) as implemented in the R limma package. Bayes statistics were only applied where there were two valid LFQ (label-free quantitative intensity) values. The adjusted *P*-values were calculated following the Benjamini & Hochberg (1995) method to correct for multiple testing.

Western blot

Western blotting on IP samples was done by running a 10% SDS–PAGE gel, and the proteins were transferred on a 0.45 µm nitrocellulose membrane (10600002 Amersham, GE Healthcare). 1% of Input and 20% of IP fraction were used for the samples to be run on the gel. The membrane was blocked with 5% BSA in PBS for 1 h at room temperature. The membrane was then incubated overnight at 4°C with anti-HA (sc805, Santa Cruz, RRID: AB_631618) at a dilution of 1:500. A goat anti-rabbit HRP conjugate (sc2004, Santa Cruz, RRID: AB_631746) in a dilution of 1:5,000 was used after washing the membrane with PBS/0.1% Tween-20 for 10 min (three times). For PTIWI01-3XFLAGHA IP, the membrane was incubated with either anti-HA (sc-7,392 HRP, Santa Cruz, RRID: AB_627809) in a dilution of 1:500 or with anti-GFP (ab290, Abcam, RRID: AB_303395) in a dilution of 1:1,000. The secondary antibody incubation was done for 1 h at room temperature, and the membrane was washed thrice with PBS/0.1% Tween-20 for 10 min. The membrane was then washed once for 5 min with 1× PBS before imaging. The membrane was scanned using chemiluminescence settings on an Amersham Imager 600 (GE Healthcare).

Phylogenetic analyses

ISWI proteins were identified (OG5_127117) and retrieved using PhyloToL (Cerón-Romero *et al*, 2019). Briefly, multi-sequence alignments were constructed using MAFFT (Katoh & Standley, 2013) and then iteratively refined with GUIDANCE2 (Sela *et al*, 2015), which identifies and removes spurious sequences and columns, preserving phylogenetically informative regions in the alignment. This refined alignment was then passed to RAxML (Stamatakis, 2014) and used to generate 200 bootstrap replicates.

Macronuclear isolation and Illumina DNA-sequencing

The samples for MAC isolation were collected from *ND7–KD*, *ISWI1–KD*, and *PTCAF1–KD* cultures from the cultures 3 days post

 © 2022 The Authors

autogamy, as described previously (Arnaiz *et al*, 2012). Paired-end libraries (Illumina TruSeq DNA, PCR-free) were made according to the standard Illumina protocol. Library preparation and sequencing were done at the NGS platform, University of Bern.

Reference genomes

The following reference genomes were used for analyzing DNA-seq data.

MAC: https://paramecium.i2bc.paris-saclay.fr/files/Paramecium/tetraurelia/51/sequences/ptetraurelia_mac_51.fa

MAC + IES: https://paramecium.i2bc.paris-saclay.fr/files/Paramecium/tetraurelia/51/sequences/ptetraurelia_mac_51_with_ies.fa

IES retention and alternative boundary analysis

IES retention scores were calculated with the MIRET component of ParTIES (Denby Wilkes *et al*, 2016). IES retention scores are provided as Source Data for Figure 2 (ISWI1_MIRET.tab).

The MILORD component (default parameters) of a pre-release version (13 August 2015) of ParTIES was used to annotate alternative and cryptic IES excision. For each IES with alternative or cryptic excision, the identifiers for the supporting reads are recorded. Output for this is provided as Dataset EV2 (CAF1_MILORD.gff3.gz, DCL23_MILORD.gff3.gz, ISWI1-b_MILORD.gff3.gz, ND7-b_MILORD.gff3.gz, and NOWA1_MILORD.gff3.gz). IRS correlations, the relationship of IRS with length, and sub-terminal frequencies were calculated as described previously (Swart *et al*, 2014). IES retention scores for *PGM/ISWI1*-KD and *PGM/ND7*–KD are provided in Source Data for Expanded View (PGM_ND7_ISWI_MIRET.tsv). The DNA-seq data for IRS correlations and alternative excision analysis apart from *ISWI1*-KDs and their corresponding controls were obtained from previous studies (ENA PRJEB12406 (Data ref: Swart *et al*, 2017b); ENA ERA309409 (Data ref: Lhuillier-Akakpo *et al*, 2014a); ENA ERS1656548 (Data ref: Furrer *et al*, 2017a) SRA SRX215498 (Data ref: Sandoval *et al*, 2014a)).

Nucleosomal DNA isolation and Illumina DNA-sequencing

Cultures for nucleosomal DNA isolation were harvested approximately 16 h after the developing macronucleus were seen. Macronuclear DNA isolation protocol was followed up to the stage of ultracentrifugation. After ultracentrifugation, the pellet containing macronucleus was washed twice with chilled 1× PBS pH 7.4, and the excess PBS was removed by centrifuging at 200 *g* for 2 min at 4°C. Half of the nuclear pellet was then recovered in 100 µl of resuspension buffer (10 mM Tris–HCl, pH 7.4, 10 mM $MgCl_2$) for MAC DNA isolation and sequencing (DNA-seq). The other half of the pellet was used for nucleosomal DNA isolation (DNase-seq). All the steps from here were optimized from the standard protocol provided with the EZ Nucleosomal DNA Prep Kit (D5220, Zymo Research). Briefly, 1 ml of chilled Nuclei Prep Buffer was used to resuspend the cell pellet before incubating on ice for 5 min. The nuclear pellet was then centrifuged at 200 *g* for 2 min at 4°C. After washing twice with Atlantis Digestion buffer, the pellet was resuspended in 1 ml of Atlantis Digestion Buffer. Five hundred microliter of the reaction was then used for DNA isolation without digestion as a control. The remaining 500 µl of the reaction was used for nucleosomal DNA isolation and 35 µl of the Atlantis dsDNAse. The reaction was incubated at 42°C for 20 min. After 20 min, the reaction was stopped by adding MN Stop Buffer, and the nucleosomal DNA isolation was carried out according to the kit protocol (D5220, Zymo Research). Note that we used Atlantis DNase for nucleosomal DNA isolation (provided in the kit D5220, Zymo Research). Atlantis dsDNase is an endonuclease specific to double-stranded DNA that cleaves phosphodiester bonds yielding oligonucleotides with 5′-phosphate and 3′-hydroxyl termini.

For *ISWI1/PGM*–KD and its control *ND7&/PGM*–KD, Illumina TruSeq PCR-free DNA library was prepared without bead-based size selection, followed by a preparative size selection on the PippinHT to remove non-ligated adaptors and library molecules with inserts > 500 bp (refer to Fig EV5B). The samples were sequenced at the NGS platform, University of Bern. For *NOWA1/2/PGM*–KD and its control *EV/PGM*–KD, the Illumina DNA Nano library preparation protocol without size selection was used. Adapter ligation was followed by bead purification to remove the non-ligated adapters. The libraries were then amplified with a library size of > 200 bp (insert + adapters; refer to Fig EV5C). The sequencing was done at Fasteris, Genesupport SA (Switzerland).

Histograms of outer distances of PE reads (Fig EV5B and C) were generated for a single representative scaffold ("scaffold51_9_with_IES") from the reference *P. tetraurelia* strain 51 MAC + IES genome, bamPEFragmentSize (with switches: "--maxFragmentLength 500 -n 1000") from the deepTools2 (Ramírez *et al*, 2016) software package was used. To obtain bins of 1 bp, in deepTools2 bamPEFragmentSize.py, for the function "getDensity", the line to generate the histogram was changed from "n, bins, patches = plt.hist(lengths, bins=100, range=(minVal, maxVal), density=True)" to "n, bins, patches = plt.hist(lengths, bins=range(-maxVal), range=(minVal, maxVal), density=True)".

DNase-seq analyses

For general nucleosome density distribution analyses, we use HISAT2 (Kim *et al*, 2019) for read mapping of nucleosomal and new MAC DNA preparations with parameters "--min-intronlen 24" and "--max-intronlen 20,000" to the reference *P. tetraurelia* strain 51 "MAC + IES" genome (Arnaiz *et al*, 2012). For nucleosome profiling, "properly paired" (as defined by the samtools (Li *et al*, 2009) flag "2") paired-end reads with an outer distance between 100 and 175 bp, in the range expected for mononucleosomes were selected for further analysis. Bedtools (Quinlan & Hall, 2010) was used to extract reads with at least 9 bp of sequences matching IESs with the parameters "-f 0.06 -split". htseq-count from the HTSeq package (Anders *et al*, 2015) was used to count IES-matching reads.

DNA-seq normalized IES nucleosome densities (dimensionless quantities, since IES length normalizations for DNA-seq and nucleosome profiling, cancel each other out), r_c and r_e (subscript c = control; subscript e = experiment), for each IES, IES_i (i = 1 to 44,925), were calculated according to the following:

$$r_c = (n_c/N_c) \div (d_c/D_c).$$

$$r_e = (n_e/N_e) \div (d_e/D_e).$$

Control = *ND7/PGM*-KD (for *ISWI1*) or *ND7/PGM*-KD (for *NOWA1/2*).

Experiment = *ISWI1/PGM*-KD or *NOWA1/2/PGM*-KD.

d_c = number of DNA reads mapped to a particular IES from control.

n_c = number of nucleosomal reads mapped to a particular IES from control.

d_e = number of DNA reads mapped to a particular IES from experiment.

n_e = number of nucleosomal reads mapped to a particular IES from experiment.

D_c = number of mapped DNA reads from control.

N_c = number of mapped nucleosomal reads from control.

D_e = downsampled number of mapped DNA reads from the experiment.

N_e = downsampled number of mapped nucleosomal reads from the experiment.

Since the number of reads between the libraries differed, we downsampled the larger ones to equivalent total numbers to the smaller ones, using the samtools (Li *et al*, 2009) version 1.7 command "samtools view -s" with the suitable fraction for the "-s" switch. The *ND7/PGM*–KD MAC DNA library (for ISWI1) was 0.7777 times the size of *ISWI1/PGM*–KD, and the *ISWI1/PGM*–KD nucleosomal library was 0.5875 times the size of *ND7/PGM*–KD. The total mapped IES read counts after downsampling were D_c = 939,549, D_e = 1,522,345; N_c = 1,017,091, N_e = 2,484,586. For *ND7/PGM*-KD (for NOWA1/2) and *NOWA1/2/PGM*-KD: D_c = 594,577, D_e = 860,348; N_c = 203,593, N_e = 231,555.

Note that the amount of IES DNA from the parental MAC is negligible compared to that from the knockdowns (compare Figs 2A vs. EV5A), both of which use the same nuclear isolation procedure. Thus, no explicit normalizations were applied to account for parental MAC DNA.

Calculations and the graphs generated are available as a Jupyter notebook (Dataset EV3; "DNase-seq_analysis.ipynb"), together with the necessary read count data (Dataset EV4, "ISWI1.IES_read_counts.txt" and, Dataset EV5, "NOWA1.IES_read_counts.txt") and IES retention score table (Dataset EV6, "ies_retention_plus_ISWI1.tab").

Data availability

The genomic datasets are available in the following databases:

- DNA-seq data: All raw sequencing data are available at the European Nucleotide Archive under the accession number PRJEB21344 (http://www.ebi.ac.uk/ena/data/view/PRJEB21344). Accession numbers for individual experiments are as follows:
  - DNA-seq for *ISWI1*-KD(a): ERR2010817
  - DNA-seq for *ISWI1*-KD(b): ERR2010816
  - DNA-seq for *PTCAF1*-KD: ERR2010818
  - DNA-seq for *ND7*-KD: ERR2010819
  - DNA-seq for *PGM/ND7*-KD: ERR2798685 DNA
  - DNA-seq for for *PGM/ISWI1*-KD: ERR2798686
  - DNase-seq for *PGM/ISWI1*-KD: ERR2798687
  - DNase-seq for *PGM/ND7*-KD: ERR2798688
  - DNA-seq for for *ND7/PGM*-KD (control for *NOWA1/2/PGM*-KD): ERS12021512
  - DNA-seq for *NOWA1/2/PGM*-KD: ERS12021513
  - DNase-seq for *NOWA1/2/PGM*-KD: ERS12021514
  - DNase-seq for *ND7/PGM*-KD (control for *NOWA1/2/PGM*-KD): ERS12021515

Expanded View for this article is available online.

Acknowledgements
We are thankful to Dr. Nasikhat Stahlberger for her technical support and to all the members of the Nowacki lab for their suggestions and fruitful discussions. We thank Christiane Emmerich for her technical support. We are also very thankful to Prof. Dr. Manfred Heller and Dr. Anne-Christine Uldry for their help with mass spectrometry and data analysis. This research was supported by grants from the European Research Council (ERC): 260358 "EPIGENOME" and 681178 "G-EDIT," Swiss National Science Foundation: 31003A_146257 and 31003A_166407 and from the National Center of Competence in Research (NCCR) RNA and Disease to MN, and the Max Planck Society (ECS).

Author contributions
Aditi Singh: Conceptualization; data curation; formal analysis; validation; investigation; visualization; methodology; writing – original draft; writing – review and editing. **Xyrus X Maurer-Alcalá:** Formal analysis; validation; investigation; writing – original draft. **Therese Solberg:** Validation; investigation; writing – review and editing. **Lilia Häußermann:** Validation; investigation; writing – review and editing. **Silvan Gisler:** Validation; investigation; writing – review and editing. **Michael Ignarski:** Validation; investigation; writing – review and editing. **Estienne C Swart:** Resources; data curation; formal analysis; supervision; funding acquisition; validation; investigation; visualization; methodology; writing – review and editing. **Mariusz Nowacki:** Conceptualization; resources; supervision; funding acquisition; methodology; project administration; writing – review and editing.

Disclosure and competing interests statement
The authors declare that they have no conflict of interest.

References

Abello A, Régnier V, Arnaiz O, Le Bars R, Bétermier M, Bischerour J (2020) Functional diversification of paramecium Ku80 paralogs safeguards genome integrity during precise programmed DNA elimination. *PLoS Genet* 16: e1008723

Anders S, Pyl PT, Huber W (2015) HTSeq — a python framework to work with high-throughput sequencing data. *Bioinformatics* 31: 166–169

Arambasic M, Sandoval PY, Hoehener C, Singh A, Swart EC, Nowacki M (2014) Pdsg1 and Pdsg2, novel proteins involved in developmental genome remodelling in paramecium. *PLoS ONE* 9: e112899

Arnaiz O, Gout J-F, Bétermier M, Bouhouche K, Cohen J, Duret L, Kapusta A, Meyer E, Sperling L (2010) Gene expression in a paleopolyploid: a transcriptome resource for the ciliate *Paramecium tetraurelia*. *BMC Genomics* 11: 547

Arnaiz O, Mathy N, Baudry C, Malinsky S, Aury J-M, Denby Wilkes C, Garnier O, Labadie K, Lauderdale BE, Le Mouël A *et al* (2012) The *Paramecium* germline genome provides a niche for intragenic parasitic DNA:

 © 2022 The Authors

evolutionary dynamics of internal eliminated sequences. *PLoS Genet* 8: e1002984

Arnaiz O, Sperling L (2011) *Paramecium*DB in 2011: new tools and new data for functional and comparative genomics of the model ciliate *Paramecium tetraurelia*. *Nucleic Acids Res* 39: D632–D636

Aury J-M, Jaillon O, Duret L, Noel B, Jubin C, Porcel BM, Ségurens B, Daubin V, Anthouard V, Aiach N *et al* (2006) Global trends of whole-genome duplications revealed by the ciliate *Paramecium tetraurelia*. *Nature* 444: 171–178

Bartholomew B (2014) Regulating the chromatin landscape: structural and mechanistic perspectives. *Annu Rev Biochem* 83: 671–696

Baudry C, Malinsky S, Restituito M, Kapusta A, Rosa S, Meyer E, Bétermier M (2009) PiggyMac, a domesticated piggyBac transposase involved in programmed genome rearrangements in the ciliate *Paramecium tetraurelia*. *Genes Dev* 23: 2478–2483

Beisson J, Bétermier M, Bré M-H, Cohen J, Duharcourt S, Duret L, Kung C, Malinsky S, Meyer E, Preer JR *et al* (2010a) *Paramecium tetraurelia*: the renaissance of an early unicellular model. *Cold Spring Harb Protoc* 2010: pdb.emo140

Beisson J, Bétermier M, Bré M-H, Cohen J, Duharcourt S, Duret L, Kung C, Malinsky S, Meyer E, Preer JR *et al* (2010b) Maintaining clonal *Paramecium tetraurelia* cell lines of controlled age through daily reisolation. *Cold Spring Harb Protoc* 2010: pdb.prot5361

Beisson J, Bétermier M, Bré M-H, Cohen J, Duharcourt S, Duret L, Kung C, Malinsky S, Meyer E, Preer JR *et al* (2010c) Silencing specific *Paramecium tetraurelia* genes by feeding double-stranded RNA. *Cold Spring Harb Protoc* 2010: pdb.prot5363

Beisson J, Bétermier M, Bré M-H, Cohen J, Duharcourt S, Duret L, Kung C, Malinsky S, Meyer E, Preer JR *et al* (2010d) DNA microinjection into the macronucleus of *Paramecium*. *Cold Spring Harb Protoc* 2010: pdb.prot5364

Benjamini Y, Hochberg Y (1995) Controlling the false discovery rate: a practical and powerful approach to multiple testing. *J R Stat Soc B Methodol* 57: 289–300

Bischerour J, Bhullar S, Denby Wilkes C, Régnier V, Mathy N, Dubois E, Singh A, Swart E, Arnaiz O, Sperling L *et al* (2018) Six domesticated PiggyBac transposases together carry out programmed DNA elimination in *Paramecium*. *eLife* 7: e37927

Bouhouche K, Gout J-F, Kapusta A, Bétermier M, Meyer E (2011) Functional specialization of Piwi proteins in *Paramecium tetraurelia* from post-transcriptional gene silencing to genome remodelling. *Nucleic Acids Res* 39: 4249–4264

Brown T (2001) Dot and slot blotting of DNA. *Curr Protoc Mol Biol* Chapter 2: Unit 2.9B

Cerón-Romero MA, Maurer-Alcalá XX, Grattepanche J-D, Yan Y, Fonseca MM, Katz LA (2019) PhyloToL: A taxon/gene-rich phylogenomic pipeline to explore genome evolution of diverse eukaryotes. *Mol Biol Evol* 36: 1831–1842

Chalker DL, Yao M-C (2011) DNA elimination in ciliates: transposon domestication and genome surveillance. *Annu Rev Genet* 45: 227–246

Clapier CR, Cairns BR (2009) The biology of chromatin remodeling complexes. *Annu Rev Biochem* 78: 273–304

Corona DF, Längst G, Clapier CR, Bonte EJ, Ferrari S, Tamkun JW, Becker PB (1999) ISWI is an ATP-dependent nucleosome remodeling factor. *Mol Cell* 3: 239–245

Corona DFV, Siriaco G, Armstrong JA, Snarskaya N, McClymont SA, Scott MP, Tamkun JW (2007) ISWI regulates higher-order chromatin structure and histone H1 assembly in vivo. *PLoS Biol* 5: e232

Coyne RS, Lhuillier-Akakpo M, Duharcourt S (2012) RNA-guided DNA rearrangements in ciliates: Is the best genome defence a good offence? *Biol Cell* 104: 309–325

Denby Wilkes C, Arnaiz O, Sperling L (2016) ParTIES: A toolbox for *Paramecium* interspersed DNA elimination studies. *Bioinformatics* 32: 599–601

Deutsch EW, Bandeira N, Sharma V, Perez-Riverol Y, Carver JJ, Kundu DJ, García-Seisdedos D, Jarnuczak AF, Hewapathirana S, Pullman BS *et al* (2020) The ProteomeXchange consortium in 2020: enabling "big data" approaches in proteomics. *Nucleic Acids Res* 48: D1145–D1152

Dirscherl SS, Krebs JE (2004) Functional diversity of ISWI complexes. *Biochem Cell Biol* 82: 482–489

Drews F, Salhab A, Karunanithi S, Cheaib M, Jung M, Schulz MH, Simon M (2022) Broad domains of histone marks in the highly compact *Paramecium* macronuclear genome. *Genome Res* 32: 710–725

Dubois E, Bischerour J, Marmignon A, Mathy N, Régnier V, Bétermier M (2012) Transposon invasion of the *Paramecium* germline genome countered by a domesticated piggybac transposase and the NHEJ pathway. *Int J Evol Biol* 2012: 436196

Duret L, Cohen J, Jubin C, Dessen P, Gout J-F, Mousset S, Aury J-M, Jaillon O, Noël B, Arnaiz O *et al* (2008) Analysis of sequence variability in the macronuclear DNA of *Paramecium tetraurelia*: a somatic view of the germline. *Genome Res* 18: 585–596

Erdel F, Krug J, Längst G, Rippe K (2011) Targeting chromatin remodelers: signals and search mechanisms. *Biochim Biophys Acta* 1809: 497–508

Fillingham JS, Garg J, Tsao N, Vythilingum N, Nishikawa T, Pearlman RE (2006) Molecular genetic analysis of an SNF2/brahma-related gene in *Tetrahymena thermophila* suggests roles in growth and nuclear development. *Eukaryot Cell* 5: 1347–1359

Frapporti A, Miró Pina C, Arnaiz O, Holoch D, Kawaguchi T, Humbert A, Eleftheriou E, Lombard B, Loew D, Sperling L *et al* (2019) The Polycomb protein Ezl1 mediates H3K9 and H3K27 methylation to repress transposable elements in *Paramecium*. *Nat Commun* 10: 2710

Furrer DI, Swart EC, Kraft MF, Sandoval PY, Nowacki M (2017a) European Nucleotide Archive (https://www.ebi.ac.uk/ena/browser/text-search?query=ERS1656548). [DATASET]

Furrer DI, Swart EC, Kraft MF, Sandoval PY, Nowacki M (2017b) Two sets of Piwi proteins are involved in distinct sRNA pathways leading to elimination of germline-specific DNA. *Cell Rep* 20: 505–520

Gnan S, Matelot M, Weiman M, Arnaiz O, Guérin F, Sperling L, Bétermier M, Thermes C, Chen C-L, Duharcourt S (2022) GC content, but not nucleosome positioning, directly contributes to intron splicing efficiency in paramecium. *Genome Res* 32: 699–709

Hamilton EP, Kapusta A, Huvos PE, Bidwell SL, Zafar N, Tang H, Hadjithomas M, Krishnakumar V, Badger JH, Caler EV *et al* (2016) Structure of the germline genome of *Tetrahymena thermophila* and relationship to the massively rearranged somatic genome. *eLife* 5: e19090

Huang B, Pan X, Li Z, Wang Z, Yu Y, Dou Z (2012) Polycomb group proteins and their roles in regulating stem cell development. *Zhongguo Yi Xue Ke Xue Yuan Xue Bao* 34: 281–285

Ignarski M, Singh A, Swart EC, Arambasic M, Sandoval PY, Nowacki M (2014) *Paramecium tetraurelia* chromatin assembly factor-1-like protein PtCAF-1 is involved in RNA-mediated control of DNA elimination. *Nucleic Acids Res* 42: 11952–11964

Kammers K, Cole RN, Tiengwe C, Ruczinski I (2015) Detecting significant changes in protein abundance. *EuPA Open Proteom* 7: 11–19

Kapusta A, Matsuda A, Marmignon A, Ku M, Silve A, Meyer E, Forney JD, Malinsky S, Bétermier M (2011) Highly precise and developmentally programmed genome assembly in *Paramecium* requires ligase IV-dependent end joining. *PLoS Genet* 7: e1002049

© 2022 The Authors

Katoh K, Standley DM (2013) MAFFT multiple sequence alignment software version 7: Improvements in performance and usability. *Mol Biol Evol* 30: 772–780

Kim D, Paggi JM, Park C, Bennett C, Salzberg SL (2019) Graph-based genome alignment and genotyping with HISAT2 and HISAT-genotype. *Nat Biotechnol* 37: 907–915

Lepère G, Nowacki M, Serrano V, Gout J-F, Guglielmi G, Duharcourt S, Meyer E (2009) Silencing-associated and meiosis-specific small RNA pathways in *Paramecium tetraurelia*. *Nucleic Acids Res* 37: 903–915

Lhuillier-Akakpo M, Frapporti A, Denby Wilkes C, Matelot M, Vervoort M, Sperling L, Duharcourt S (2014a) European Nucleotide Archive (https://www.ebi.ac.uk/ena/browser/view/ERA309409). [DATASET]

Lhuillier-Akakpo M, Frapporti A, Denby Wilkes C, Matelot M, Vervoort M, Sperling L, Duharcourt S (2014b) Local effect of enhancer of zeste-like reveals cooperation of epigenetic and cis-acting determinants for zygotic genome rearrangements. *PLoS Genet* 10: e1004665

Liu Y, Taverna SD, Muratore TL, Shabanowitz J, Hunt DF, Allis CD (2007) RNAi-dependent H3K27 methylation is required for heterochromatin formation and DNA elimination in Tetrahymena. *Genes Dev* 21: 1530–1545

Li H, Handsaker B, Wysoker A, Fennell T, Ruan J, Homer N, Marth G, Abecasis G, Durbin R, 1000 Genome Project Data Processing Subgroup (2009) The sequence alignment/map format and SAMtools. *Bioinformatics* 25: 2078–2079

Li Z, Gadue P, Chen K, Jiao Y, Tuteja G, Schug J, Li W, Kaestner KH (2012) Foxa2 and H2A.Z mediate nucleosome depletion during embryonic stem cell differentiation. *Cell* 151: 1608–1616

Maliszewska-Olejniczak K, Gruchota J, Gromadka R, Denby Wilkes C, Arnaiz O, Mathy N, Duharcourt S, Bétermier M, Nowak JK (2015) TFIIS-dependent non-coding transcription regulates developmental genome rearrangements. *PLoS Genet* 11: e1005383

Margueron R, Reinberg D (2011) The Polycomb complex PRC2 and its mark in life. *Nature* 469: 343–349

Miró-Pina C, Charmant O, Kawaguchi T, Holoch D, Michaud A, Cohen I, Humbert A, Jaszczyszyn Y, Chevreux G, Del Maestro L *et al* (2022) Paramecium Polycomb repressive complex 2 physically interacts with the small RNA-binding PIWI protein to repress transposable elements. *Dev Cell* 57: 1037–1052

Nowacki M, Zagorski-Ostoja W, Meyer E (2005) Nowa1p and Nowa2p: novel putative RNA binding proteins involved in trans-nuclear crosstalk in paramecium tetraurelia. *Curr Biol* 15: 1616–1628

Pazin MJ, Kadonaga JT (1997) SWI2/SNF2 and related proteins: ATP-driven motors that disrupt protein-DNA interactions? *Cell* 88: 737–740

Perez-Riverol Y, Csordas A, Bai J, Bernal-Llinares M, Hewapathirana S, Kundu DJ, Inuganti A, Griss J, Mayer G, Eisenacher M *et al* (2019) The PRIDE database and related tools and resources in 2019: improving support for quantification data. *Nucleic Acids Res* 47: D442–D450

Pulivarthy SR, Lion M, Kuzu G, Matthews AGW, Borowsky ML, Morris J, Kingston RE, Dennis JH, Tolstorukov MY, Oettinger MA (2016) Regulated large-scale nucleosome density patterns, and precise nucleosome positioning correlate with V(D)J recombination. *Proc Natl Acad Sci USA* 113: E6427–E6436

Quinlan AR, Hall IM (2010) BEDTools: a flexible suite of utilities for comparing genomic features. *Bioinformatics* 26: 841–842

Ramírez F, Ryan DP, Grüning B, Bhardwaj V, Kilpert F, Richter AS, Heyne S, Dündar F, Manke T (2016) deepTools2: A next generation web server for deep-sequencing data analysis. *Nucleic Acids Res* 44: W160–W165

Rando OJ, Winston F (2012) Chromatin and transcription in yeast. *Genetics* 190: 351–387

Sadeh R, Allis CD (2011) Genome-wide "re"-modeling of nucleosome positions. *Cell* 147: 263–266

Sandoval PY, Swart EC, Arambasic M, Nowacki M (2014a) European Nucleotide Archive (https://www.ebi.ac.uk/ena/browser/view/SRX309864?show=reads). [DATASET]

Sandoval PY, Swart EC, Arambasic M, Nowacki M (2014b) Functional diversification of dicer-like proteins and small RNAs required for genome sculpting. *Dev Cell* 28: 174–188

Sela I, Ashkenazy H, Katoh K, Pupko T (2015) GUIDANCE2: Accurate detection of unreliable alignment regions accounting for the uncertainty of multiple parameters. *Nucleic Acids Res* 43: W7–W14

Skouri F, Cohen J (1997) Genetic approach to regulated exocytosis using functional complementation in paramecium: Identification of the ND7 gene required for membrane fusion. *Mol Biol Cell* 8: 1063–1071

Stamatakis A (2014) RAxML version 8: A tool for phylogenetic analysis and post-analysis of large phylogenies. *Bioinformatics* 30: 1312–1313

Swart EC, Denby Wilkes C, Sandoval PY, Hoehener C, Singh A, Furrer DI, Arambasic M, Ignarski M, Nowacki M (2017a) Identification and analysis of functional associations among natural eukaryotic genome editing components [version 1; peer review: 1 approved, 1 approved with reservations]. *F1000Res* 6: 1374

Swart EC, Denby Wilkes C, Sandoval PY, Hoehener C, Singh A, Furrer DI, Arambasic M, Ignarski M, Nowacki M (2017b) European Nucleotide Archive (https://www.ebi.ac.uk/ena/browser/view/PRJEB12406?show=reads). [DATASET]

Swart EC, Wilkes CD, Sandoval PY, Arambasic M, Sperling L, Nowacki M (2014) Genome-wide analysis of genetic and epigenetic control of programmed DNA deletion. *Nucleic Acids Res* 42: 8970–8983

Wasmuth EV, Lima CD (2017) The Rrp6 C-terminal domain binds RNA and activates the nuclear RNA exosome. *Nucleic Acids Res* 45: 846–860

Xiao H, Sandaltzopoulos R, Wang HM, Hamiche A, Ranallo R, Lee KM, Fu D, Wu C (2001) Dual functions of largest NURF subunit NURF301 in nucleosome sliding and transcription factor interactions. *Mol Cell* 8: 531–543

Xu J, Zhao X, Mao F, Basrur V, Ueberheide B, Chait BT, Allis CD, Taverna SD, Gao S, Wang W *et al* (2021) A Polycomb repressive complex is required for RNAi-mediated heterochromatin formation and dynamic distribution of nuclear bodies. *Nucleic Acids Res* 49: 5407–5425

Yadon AN, Tsukiyama T (2011) SnapShot: chromatin remodeling: ISWI. *Cell* 144: 453

License: This is an open access article under the terms of the Creative Commons Attribution License, which permits use, distribution and reproduction in any medium, provided the original work is properly cited.

Expanded View Figures

Figure EV1. Knockdown effects of ISWI paralogs in *Paramecium tetraurelia*.

A, B Northern blot analysis using ISWI1-specific and ISWI5-specific probes, respectively. rRNA probe was used as a loading control against ribosomal RNA. Early: ~50% of cells with fragmented parental macronucleus; Late: the majority of cells with a visible anlagen. *ND7*-KD is used as a control to confirm mRNA expression.

C–F (C and E) Survival test graph. Dead cells are represented in red, sick in orange and cells diving at a normal rate in green. *PGM*–KD is used as a positive control, and *ND7*–KD as a negative control. (D and F) IES retention PCR (cropped inverted images). Five maternally controlled IES and five non-maternally controlled IESs are shown. The IES+ band represents retained IES; the IES-band represents excised IES; additional bands are likely PCR artifacts or primer dimers.

Source data are available online for this figure.

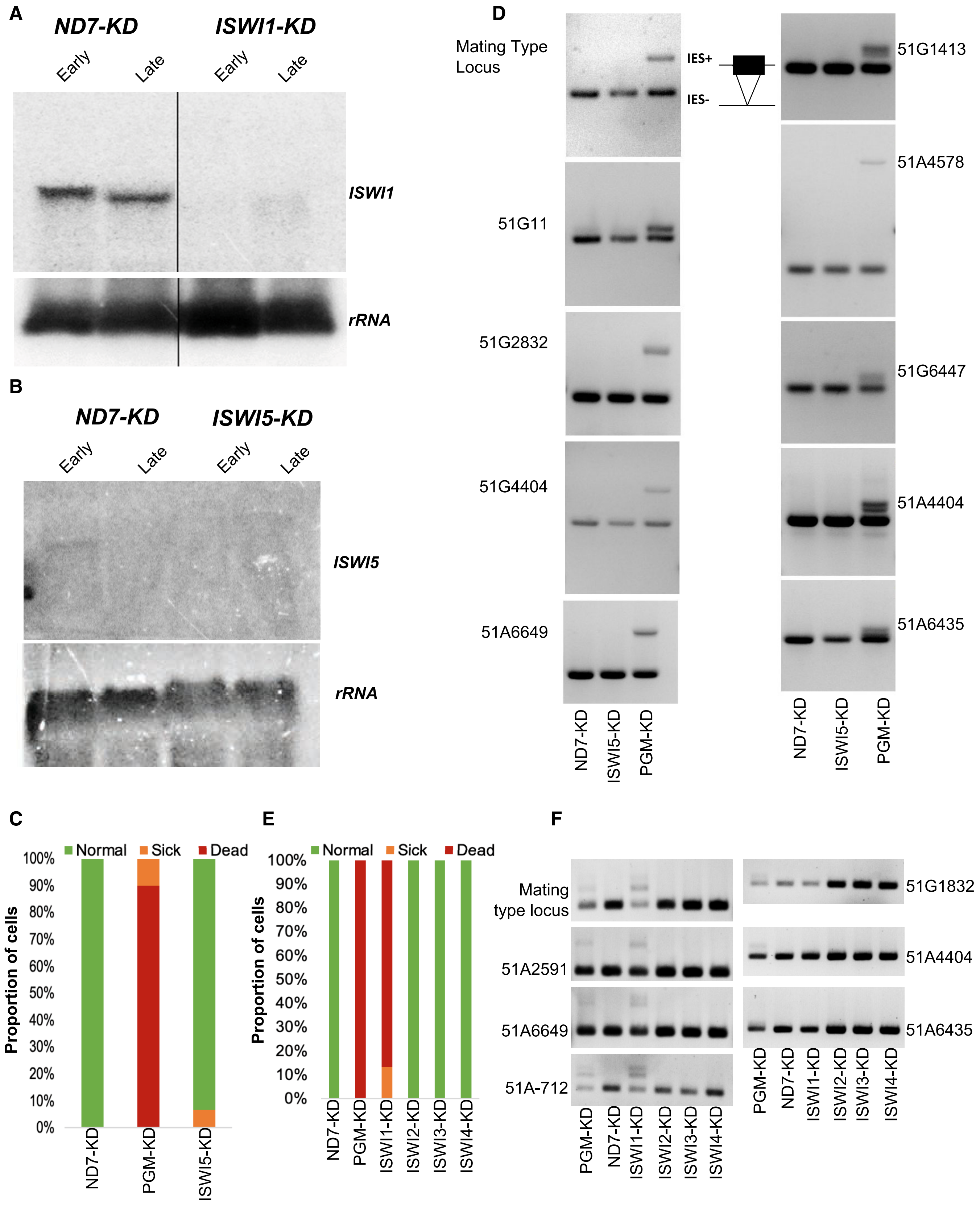

Figure EV1.

 © 2022 The Authors

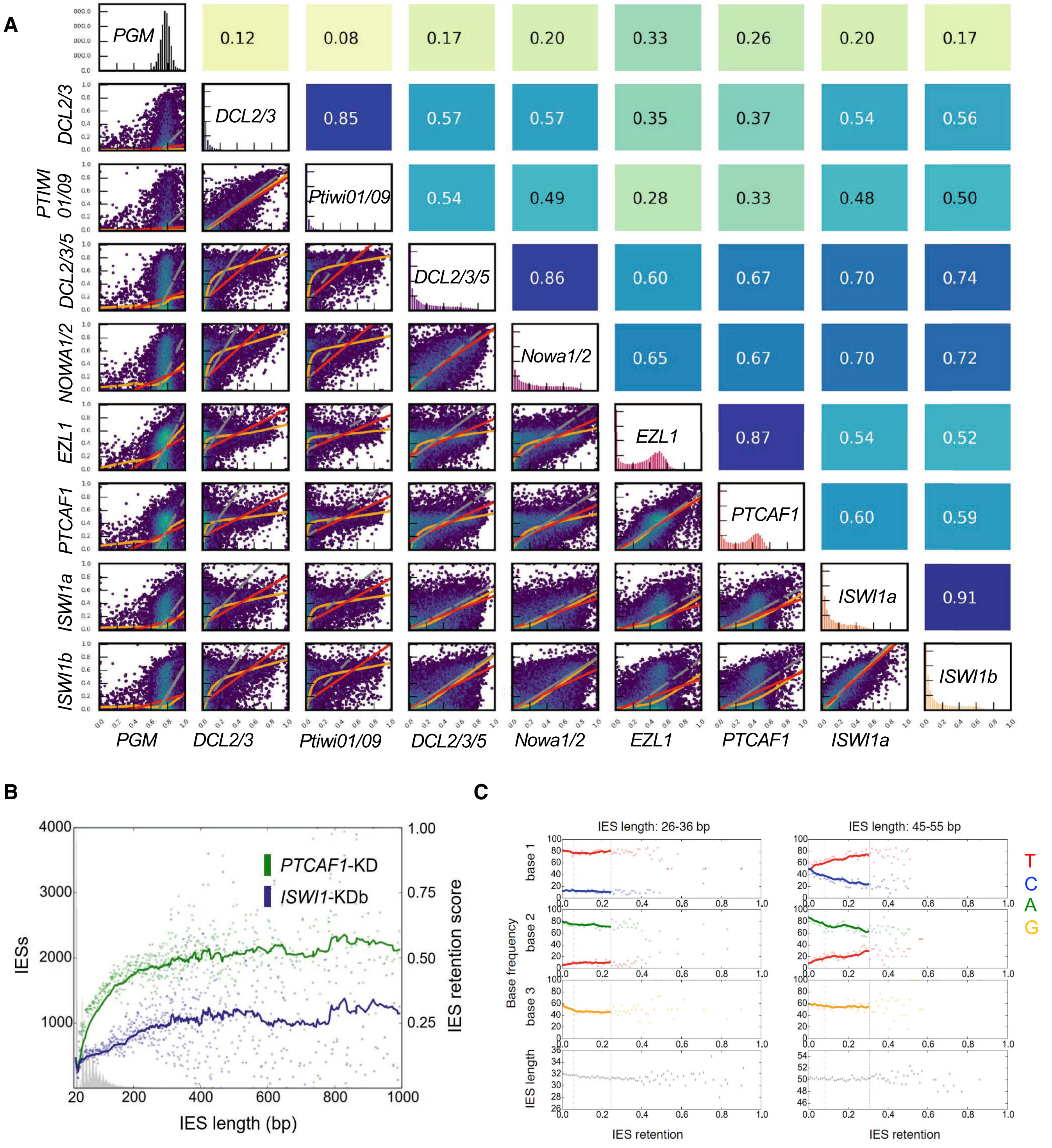

Figure EV2. Relationship between IES retention scores, IES length, and base sequences.

A Relationships in IES retention among knockdown pairs. Hexagonal binning of IES retention scores was used to generate the plots. Pearson's correlation coefficients are given above each subgraph. Red lines are for ordinary least-squares (OLS) regression, orange lines are for LOWESS, and gray lines are for orthogonal distance regression (ODR).

B IRSs versus IES length as described previously.

C Base frequencies of the first three bases after the TA repeat relative to the IRS of *ISWI1*-KDb from the first and third *Paramecium tetraurelia* IES length peak.

© 2022 The Authors

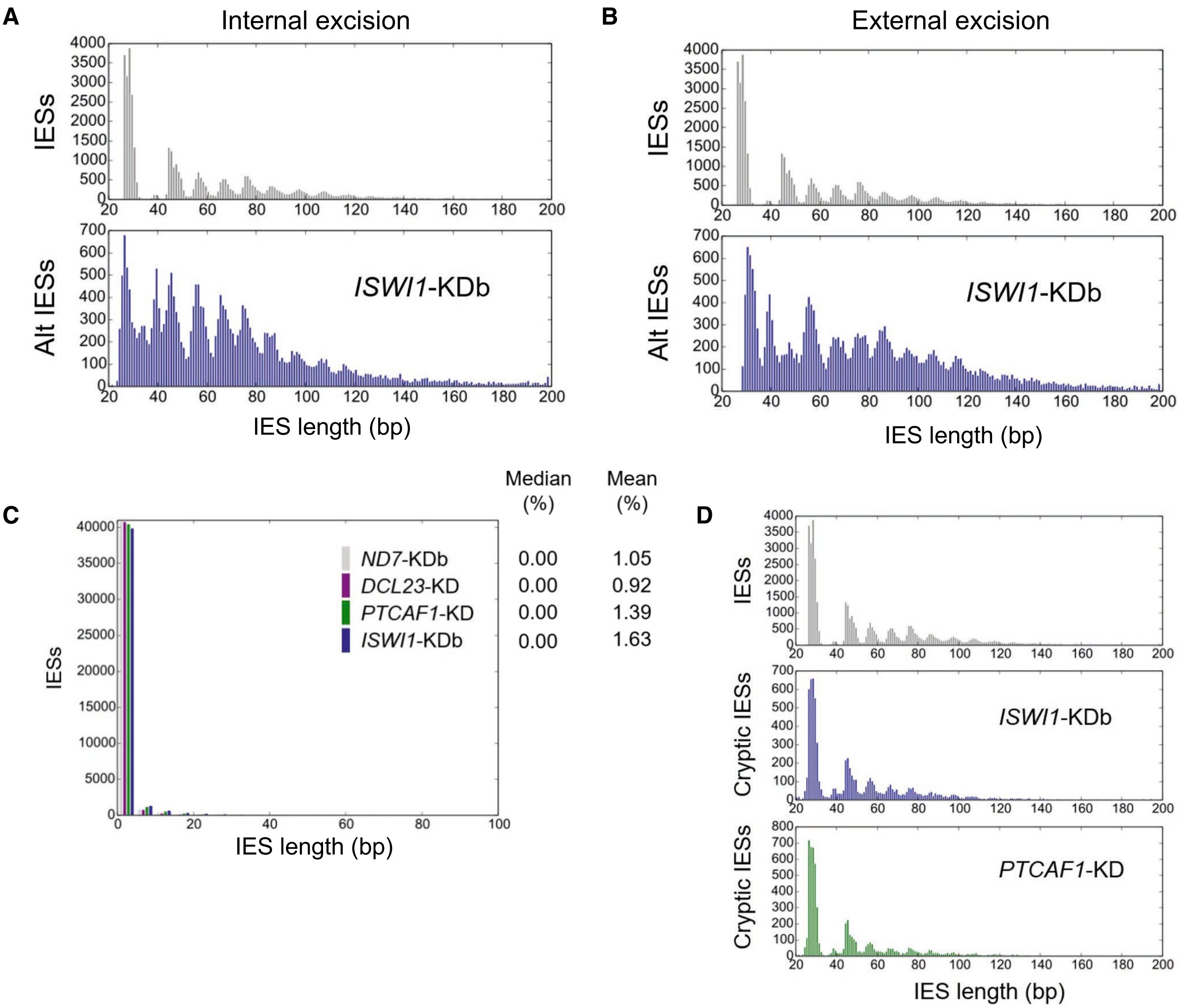

Figure EV3. Size distribution of Alternatively excised IESs and Cryptic IESs.

A Length distribution of internally excised alternative (Alt) IES boundaries.

B Length distribution of externally alternative (Alt) excised IES boundaries, respectively.

C Genome-wide analysis of cryptic IES excision. Cryptic excision (%) = 100*(cryptically excised reads)/(all reads).

D Length distribution of cryptically excised IES.

 © 2022 The Authors

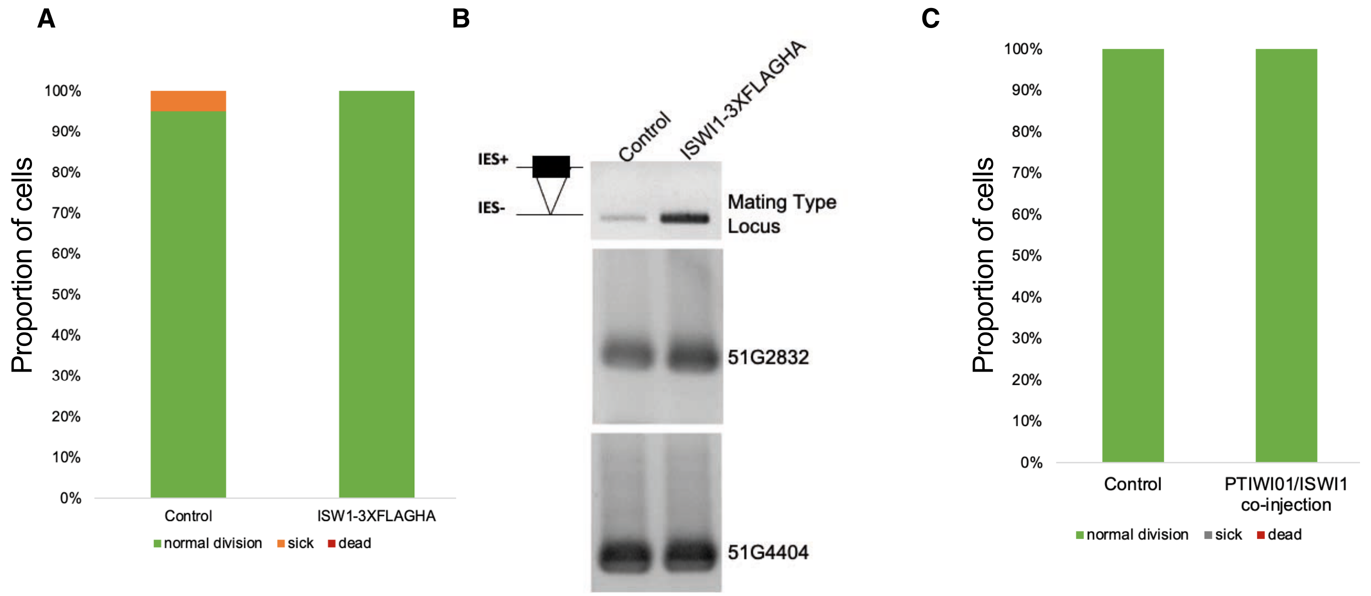

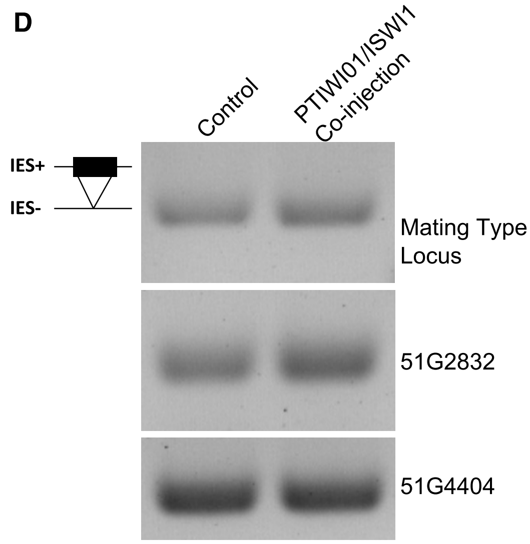

E

Majority protein IDs	Peptide Count ISWI1	Peptide count ISWI2	Peptide count WT1	Peptide count WT2	*p* BH adjusted
ISWI1	301	329	0	0	n.d.
Uncharacterized protein	90	104	0	0	n.d.
DDRP	62	61	0	0	n.d.
SPT6	49	45	0	0	n.d.
PTIWI01	26	43	2	3	0,004
NOWA2	11	9	0	0	n.d.

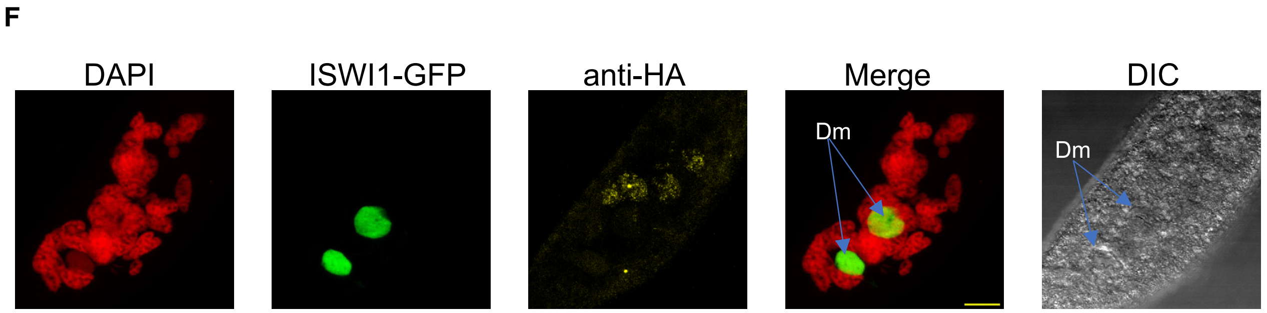

Figure EV4.

Figure EV4. Overexpression of fusion proteins do not show any adverse effects.

A–D (A and C) Survival test graph. Dead cells are represented in red, sick in orange and cells diving at a normal rate in green. (B and D) IES retention PCR (cropped inverted images). Mating type, 51G2832, and 51G4404 IESs are shown. The IES+ band represents retained IES; the IES-band represents excised IES; additional bands are likely PCR artifacts or primer dimers.

E Most abundant proteins in the ISWI1-3XFLAGHA MS analysis ISWI1 & ISWI2 are two biological replicates for ISWI1-3XFLAGHA, while WT1 and WT2 are biological replicates for control in MS analysis. Peptide count refers to the number of peptides detected in MS. Adjusted *P*-values were calculated following the Benjamini and Hochberg correction for multiple testing.

F ISWI1-GFP localization to developing macronucleus seen in green during development; merge is an overlay of DAPI (red) staining parental and developing macronucleus, ISWI1-GFP (green), and anti-HA (yellow); scale bar = 10 μm.

Source data are available online for this figure.

Figure EV5. Nucleosome density measurements after DNase-seq.

A IES Retention Score (IRS) distributions for *PGM/ND7*-KD and *PGM/ISWI1*-KD. (B, C) Histograms of outer paired-end distances of mapped DNase-seq reads.

D–I Normalized nucleosome density histograms for IESs weakly (IRS < 0.2) or more strongly retained in *ISWI1*-Kdb (IRS ≥ 0.2), either for *ND7/PGM*-KD or *NOWA1/PGM*-KD. Kolmogorov–Smirnov statistics and their *P*-values are provided. Titles for graphs give criteria for IES selection.

Source data are available online for this figure.

 © 2022 The Authors

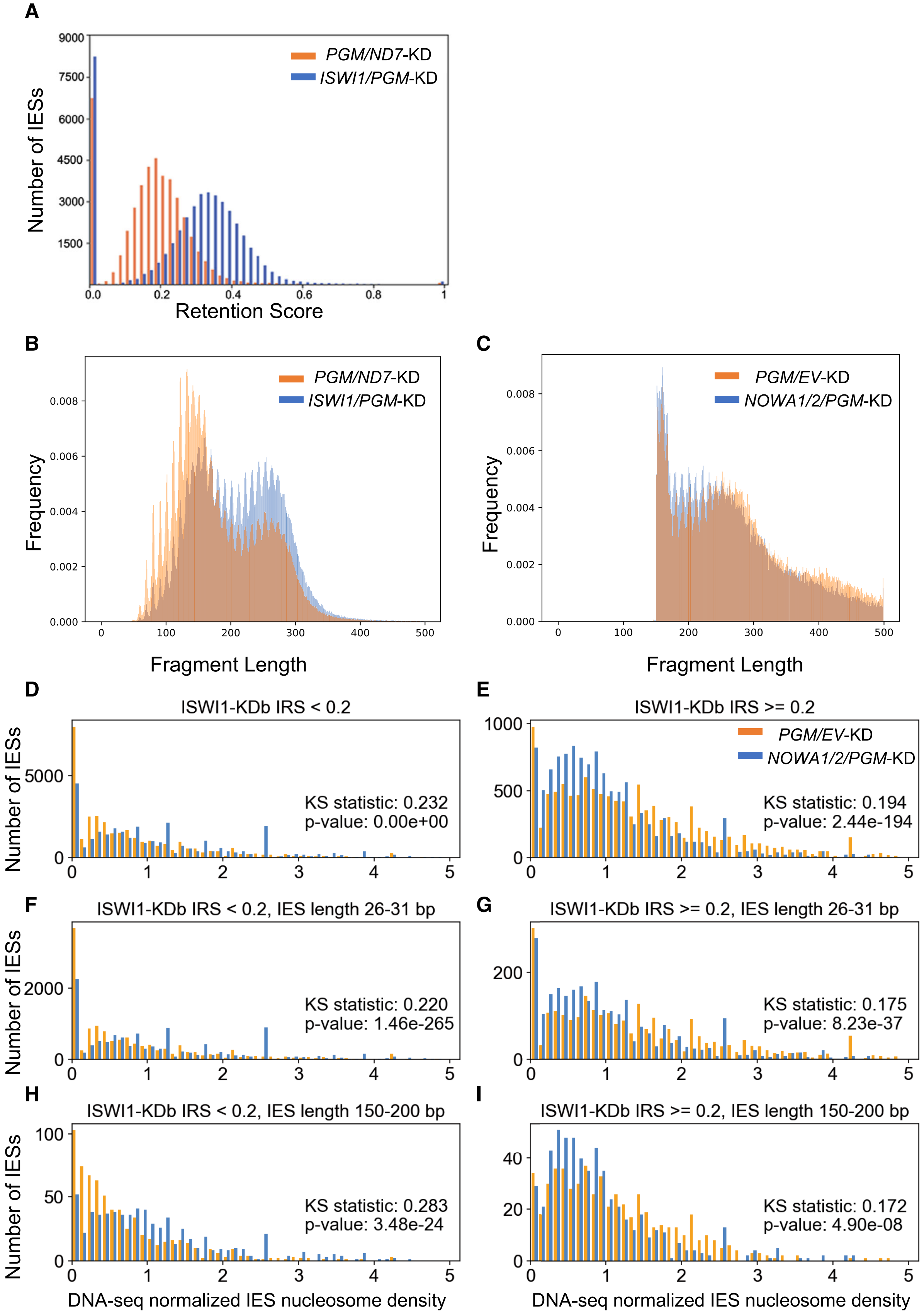

Figure EV5.

© 2022 The Authors

Appendix

Table of Contents **Page number**

Table of Contents	Page number
Appendix Table S1	2
Appendix Table S2	3
Appendix Table S3	4

Appendix Table S1

IES	Primer sequence (5' to 3' orientation)
51G--11F	ATCATAAGATTGATATCTTCTCCCTTCTCC
51G--11R	ACTTGCTACTAAAGCAAGAAACATTGAGAG
51G1413F	GAAGCTGCTTGTGTTAAGAATTCTACTGG
51G1413R	GCATCCAGCACTAGTTGAATTTACTGTAC
51G1832F	CTATAACTCTTGAAGCTGCTTGTAATATG
51G1832R	TTGTCAATGAGCCATTAACAGTTGCTGGAT
51G2832F	GAGCAGGATGTACAAATACTGGTGG
51G2832R	AGCTGATTAGATAACAATACAACCAGTACC
51G4404F	CTGTTGCTACACATTGTGCATATGTTACT
51G4404R	GCTGTAAGATTAACATTGAGCATGATCAAG
51G6447F	AATGCATCAAATGTAGTAACTACTCCTGCT
51G6447R	AATTTGTAAAGTATCCAGCGCAGGCAG
MT Locus F	GGTGTTTATATCTTAATTGTTGACCCTCAC
MT Locus R	CCATCTATACTCCATTCTTTATCTTAATTCAT
51A--712F	TTTGTCAAAAAGACATGTATCAAAATGCAG
51A--712R	TAGAATACTAAGAGATTCAATACAACAAAC
51A1835F	TAATGTATTGATAAGGCTTGCTCTACAGCC
51A1835R	ATCCTAACATCCTTGAATAGTTACTGATCC
51A2591F	ATGTGTTTGGACTGGATTGGCATGTAGAAG
51A2591R	GATGTAGCATAACATTTATCAACAATCCAT
51A4404F	TGGAATAGTGCTGCATCACCAGCTGCTTGC
51A4404R	CCAGTTATTGAACTGCAACTTACTGCAGTG
51A4578F	CACTGCAGTAAGTTGCAGTTCAATAACTGG
51A4578R	TGTAGTCTTAAAATCTTAGCATGTTGTACC
51A6435F	CAAATTGTGTCACTAGAGGTACATGTTTCC
51A6435R	GCGACATCAATAGTAACAGCTGAGCATGAG
51A6649F	ACTGCACCTCTAACTTTAACAAGCGAAGCA
51A6649R	CAGCAGTACATCCAGCTCTCTAAGTTTAGC
51-429F	GTT GGA TAT GCA TCC ACA TC
51-429R	CTG CTT CGA TAT GCA TAA GAA AG

Appendix Table S1: Primers used in IES retention PCRs. F: forward primer; R: reverse primer.

Appendix Table S2

Majority protein IDs	MS/MS count ISWI1	MS/MS count ISWI2	MS/MS count WT1	MS/MS count WT2	Entrez_Protein	Putative Description
PTET.51.1.P0140243	243	261	0	0	XP_001431605	ISWI1 (this manuscript)
PTET.51.1.P0440186	90	104	0	0	XP_001447805.1	Uncharacterized
PTET.51.1.P0140243	58	68	0	0	XP_001431606	ISWI1 (C-terminal)
PTET.51.1.P1370127	62	61	0	0	XP_001431060	DDRP
PTETP2700007001	61	50	0	0	XP_001441411	NAD () ADP-ribosyltransferase-3
PTETP300037001	49	45	0	0	XP_001442677	SPT6
PTET.51.1.P0420126	42	49	0	0	XP_001447124	ISWI2 (this manuscript
PTETP7100004001	26	43	2	3	XP_001456124	PTIWI01
PTET.51.1.P0180124	33	36	0	0	XP_001437349	Uncharacterized
PTET.51.1.P0070284	39	28	0	0	XP_001455634	Poly (ADP-ribose) Polymerase
PTET.51.1.P0480005	34	29	2	0	XP_001448777	DDRP2
PTETP10500011001	31	33	0	0	XP_001424861	SPT16
PTET.51.1.P0110326	19	26	3	8	XP_001425954	S-adenosyl methionine synthetase
PTET.51.1.P0330075	26	20	0	0	XP_001443869	SMC2
PTET.51.1.P0560063	17	22	2	2	XP_001451492	RuvB-like 2
PTET.51.1.P0720184	19	20	0	1	XP_001456414	Exportin
PTET.51.1.P0990120	17	22	0	0	XP_001462544	MCM6
PTETP13700005001	19	17	0	0	XP_001431029	PARP
PTET.51.1.P0040036	18	17	0	0	XP_001446004	sequence specific DNA binidng
PTET.51.1.P1720081	19	15	0	0	XP_001436697	Myb-like protein
PTET.51.1.P0610231	17	16	0	0	XP_001453395	structural constituent of ribososme
PTET.51.1.P0380048	17	15	0	0	XP_001445418	SPT5v
PTET.51.1.P0200322	14	16	0	0	XP_001439097	PHD-type
PTET.51.1.P1060046	11	16	0	0	XP_001425020	WD40-repeat like
PTET.51.1.P0030037	9	15	2	1	XP_001442334	RuvB-like 1
PTET.51.1.P1600114	9	16	0	0	XP_001435111	Erythrocyte membrane protein 3
PTET.51.1.P0470202	10	15	0	0	XP_001448725	Helicase W08D27
PTET.51.1.P0410183	12	13	0	0	XP_001446881	Uncharacterized protein
PTET.51.1.P1180147	7	16	0	0	XP_001427491	MCM2
PTET.51.1.P006001	9	10	3	0	XP_001452484	Uncharacterized protein
PTET.51.1.P0620215	10	11	0	0	XP_001453643	PTIWI11
PTET.51.1.P0170077	11	9	0	0	XP_001449252	NOWA2
PTET.51.1.P0400053	12	8	0	0	XP_001436180	DNA directed DNA polymerase
PTET.51.1.P0450314	6	12	0	0	XP_001448195	Uncharacterized protein
PTET.51.1.P0080258	8	10	0	0	XP_001458158	Uncharacterized protein

Appendix Table S2: Mass spectrometry analysis of ISWI1-3XFLAGHA co-immunoprecipitation (IP). Majority protein IDs correspond to the *Paramecium* Database (Arnaiz and Sperling, 2011) accession numbers of the proteins identified by MS. MS/MS count ISWI1 & MS/MS count ISWI1 represents total peptide count in ISWI1-3XFLAGHA IP replicates. MS/MS count WT1 & MS/MS WT2 represents total peptide count in negative control to IP. MS/MS count represents combined peptide counts in the replicates. Putative description is retrieved from Paramecium Database (https://paramecium.i2bc.paris-saclay.fr/)

Appendix Table S3

IES ID	IES Name	*PGM* -KD	*DCL2/3* -KD	*DCL5*KD	*DCL2/3/5* -KD	*NOWA1/2* -KD	*ISWI1* -KD
IESPGM.PTET51.1.51.451201	51G-11	0.79	0.05	0.19	0.56	0.53	0.48
IESPGM.PTET51.1.51.452624	51G1413	0.78	0.01	0.01	0.04	0.15	0.07
IESPGM.PTET51.1.51.453043	51G1832	0.73	0.00	0.00	0.02	0.02	0.04
IESPGM.PTET51.1.51.454043	51G2832	0.77	0.22	0.00	0.71	0.59	0.20
IESPGM.PTET51.1.51.455615	51G4404	0.84	0.60	0.00	0.75	0.80	0.58
IESPGM.PTET51.1.51.457658	51G6447	0.77	0.04	0.00	0.00	0.05	0.08
IESPGM.PTET51.1.106.281631	51A-712	0.78	0.07	0.05	0.71	0.75	0.62
IESPGM.PTET51.1.106.284157	51A1835	0.80	0.00	0.00	0.04	0.03	0.03
IESPGM.PTET51.1.106.284913	51A2591	0.91	0.54	0.00	0.80	0.81	0.68
IESPGM.PTET51.1.106.286750	51A4404	0.82	0.00	0.00	0.00	0.00	0.23
IESPGM.PTET51.1.106.286924	51A4578	0.77	0.04	0.00	0.07	0.20	0.10
IESPGM.PTET51.1.106.288781	51A6435	0.77	0.00	0.00	0.00	0.01	0.04
IESPGM.PTET51.1.106.288995	51A6649	0.81	0.55	0.01	0.73	0.73	0.61

Appendix Table S3: Comparison of IES retention scores. IESs are those that were used to test retention using standard primers (Table T1) against IESs described in (Duharcourt et al. 1998).
