# Supplementary material for: Chromatin remodeling is required for sRNA‐guided DNA elimination in Paramecium
Source: EMBO J. 2022 Oct 11;41(22):e111839. doi: 10.15252/embj.2022111839 (PMC9670198; doi:10.15252/embj.2022111839)
Supplement: Supplementary file 14 [file EMBJ-41-e111839-s013.pdf]

THE EMBO JOURNAL

Chromatin remodeling is required for sRNA-guided DNA elimination in Paramecium

Aditi Singh, Xyrus Maurer-Alcalá, Therese Solberg, Lilia Haeussermann, Silvan Gisler, Michael Ignarski, Estienne Swart, and Mariusz Nowacki
DOI: 10.15252/embj.2022111839

Corresponding author(s): Mariusz Nowacki (mariusz.nowacki@unibe.ch)

Review Timeline:

Submission Date:	8th Jun 22
Editorial Decision:	11th Jul 22
Revision Received:	12th Aug 22
Editorial Decision:	26th Aug 22
Revision Received:	14th Sep 22
Accepted:	19th Sep 22

Editor: Stefanie Boehm

Transaction Report:

(Note: With the exception of the correction of typographical or spelling errors that could be a source of ambiguity, letters and reports are not edited. Depending on transfer agreements, referee reports obtained elsewhere may or may not be included in this compilation. Referee reports are anonymous unless the Referee chooses to sign their reports.)

1st Editorial Decision 11th Jul 2022

Thank you for submitting your manuscript characterizing the role of ISWI1 during Paramecium somatic macronucleus development to The EMBO Journal. We have now received three referee reports on your study, which are included below for your information. In light of these comments, we would like to invite you to prepare and submit a revised manuscript.

As you will see, the reviewers are overall positive and appreciate the findings and their interest to the field. The referees nonetheless do raise a number of points, which can likely largely be addressed by textual revision and/or adding further discussion. Therefore, I would ask you to please carefully consider all referee comments and revise the manuscript and figures as needed, in addition to providing a detailed response to each comment. Please also remember that the revised manuscript should fulfill all EMBO Journal formatting requirements when it is next submitted (please see below and: https://www.embopress.org/page/journal/14602075/authorguide#submissionofrevisions)

Referee #1:

Singh et al. describe the molecular function of a new component of the DNA elimination pathways in Paramecium. Paramecium serves as a model for genetics and epigenetic phenomena for a long time, one of the most prominent mechanisms described is the scnRNA pathway which transmits parental information about the DNA content to progeny. As exciting this mechanism is, in particular the transgenerational transport of RNA in association with its function to alter the DNA content of F1 Macronuclei, this mechanism still holds many secrets.
Singh et al. solve one of those. It was long described that domesticated transposases cleave DNA of the IES elements in the zygotic nucleus to form a functional gene unit. This paper here adds novel information about the removal of nucleosomes to allow the endonuclease to access the DNA.
As this paper clearly adds important new information and the general subject of transgenerational inheritance is of overall importance, this work adds a significant contribution to the understanding of genome editing in ciliates as well as to the general understanding to the role of non-coding RNA controlling epigenetics and genetics. The authors summarize their new data into a new model for IES excision which extents previous models by the nucleosome data. Being more realistic, the new more model now can explain for the first time for IES size distribution and thus for evolutionary scenario of IES development.
I add below some points to improve the conclusions and clearness of the paper.

Major points:

1. I struggle with the normalization of reads for the nucleosome density. The authors use co-feeding and correctly argue that dilution of the dsRNA trigger causes milder silencing. They use therefore co-feeding of PGM with ND7, an unrelated gene as a control for ISWI/PGM co-silencing. (By the way, page 13 introduces PGM-KD alone without co-silencing, which confused me during the first reading).
Ok then, Suppl Fig. S4 clearly shows that IES retention is much higher in the PGM/ISWI KD compared to the PGM/ND7 control. This is first of all reasonable.
But then, I cannot follow the normalization applied: "DNAse-seq reads normalized by DNA-Seq isolated from new Macs". Does it mean that parts of the cultures were grown to vegetative cultures and undigested classical Mac genomes were sequenced? Please explain more in detail and also for a broad readership not necessarily familiar with Paramecium. The Figure legend also adds another factor to address, the contamination with parental Mac DNA. Please explain the details of how normalization works for this aspect.

2. Second, I have questions to which extent the two library preparation, more precisely the two fragmentation methods, differ in their biases. The DNAse will surely have a bias for GC content, then it's the question to which extent this is reflected by the normalization control. I totally agree with many other papers that the digestion of naked DNA instead of chromatin is not always the right control for the bias of MNase like analyses as for instance only a certain size fraction is analyzed. I miss an analysis and a more detailed description of the size distributions of digested DNA and the of the library. Maybe also a the inclusion of a nucleosome prediction by DNA sequence would shed light into the nature of IESs, Fig. 4H suggests a phased nucleosome pattern it would be interesting to see whether this is due at least to some extent to cis-factors in the DNA sequence.

3. I have another concern: what about IES-concatemers? If Pgm silencing is not 100% efficient due to co-silencing, a certain percentage of reads in digested DNA could come from concatemers, or should they absent from nucleosomes? Being transcribed they should have some nucleosomes, at least the larger ones. Could one screen for concatemers in the libraries, maybe also for some individual ones as indicators.

4. Maybe I missed, but Figs 4G and H are not included in the main text. Please enlarge the X-axis scale so that one can directly identify the size of the IESs. For the large IES, it seems that ISWI silencing inhibits removal of nucleosomes except the MDS marginal ones directly flanking the IES, let's say -1 and +1 of the MDSs in reference to the IES. As I understand, these are individual IESs? This is not explained.
Is this effect of the -1 and +1 nucleosomes true for all longer IESs? Could this be related to the Ku70/Ku80 binding? I think the deeper analysis of this could result in deeper insight in the mechanism, related to IES recognition (see later comments). Maybe it's worth to analyze all IESs like this thinking about a good visualization for that.....

5. This section of the DNAse results is difficult to follow. Page 13-15 are not straight forward, the discussion of the co-feeding interrupts the flow and as the main related Figure to that with the IES retention score of the PGM co-feeding is in the Supplementary data. I agree its necessary to include the RNAi efficiency, but a re-arrangement here would make it much easier for the reader.

6. I am not sure if I understand the meaning of the inclusion of the Nowa1/2/Pgm knock down analysis. As I understand Nowa1 acts upstream of ISWI and Pgm, this its inactivation leads to less ISWI activation and thus to similar to the ISWI knockdown. I am asking whether the entire comparison of IES retention rates in relation to genome rearrangement components on the individual aspects of co-silencing (triple with Pgm) is really meaningful for the interpretation of ISWI/Pgm RNAi in relation to ND7/Pgm. If it is just a control for double silencing then please make this more clear, these long sentences like page 15 second paragraph cannot be followed, at least by myself.
Minor points:

1. In the introduction and in the discussion the authors mention, that no evidence shows H3K927me3/9me3 at IESs. That's right. It is claimed that these marks are also present in the old Mac, their loss in Ezl1 silencing may alter gene expression and this expression of genes involved in DNA elimination. That's indeed possible, but the authors should not leave the reader alone with that but offer then a hypothesis what's the scnRNA doing instead of triggering heterochromatic marks. Their model does not involve histone modifications at all. Would the author's alternative model for IESs histone modifications be that the scnRNA in Ptiwi1 directly triggers removal of the nucleosome independent of histone methylation? This should be discussed and involved in the conclusions. In this context, previous papers reported about a classification of IESs due to their genetic requirements for excision. Do scnRNA independent IESs, I think these are the old ones, show ISWI sensitivity? Would that mean that ISWI can be attracted to IESs independent of scnRNAs?
2. In context of the latter comment: Ptiwi11 was also found the MS/MS data in Suppl. Table 2. That's amazing. Could one speculate that also the IES RNAs could target ISWI to the IES? This would be a support of the former hypothesis that the IES RNAs cause a feed forward loop for efficient IES excision.

3. Again, in context of the first comment here (I feel that some aspects of the literature should be included into the discussion), Miro-Pina et al. 2022 showed the interaction between Ptiwi09 and Ezl1 and components. I am aware that this paper mainly addresses TE elements and the methods for detection of interaction partners are not comparable and its never possible to discuss a protein NOT found in MS. But could there be a distinction between Ptiwi1 and Ptiwi09 the latter responsible for histone modification, Ptiwi01 for ISWI?
4. In order to make this paper better to follow for a broader non ciliate audience, the introduction of the forbidden peak on page 9 cannot be understood by an explanation in a single line. Please elaborate this more in detail here, to allow the reader to follow your attention to these IESs before you come to the model.
5. Please explain the properties of Atlantis DNAse in contrast to classical MNAse.
6. Page 13: PGM is not the last step in DNA elimination, this is I think the ligation of ends....

Referee #2:

The manuscript "Chromatin remodeling is required for sRNA-guided DNA elimination" by Singh et al., significantly advances current knowledge about contributions of nucleosomal positioning to ciliate DNA excision of IES. The authors state that, while small RNAs and histone PTMs are appreciated as a major factor in guiding DNA elimination in many ciliates, in Paramecium, the smaller, subnucleosomal size of many IES, lack of known PTMs physically associated with IES, and additional proteins found to be required for excision suggests additional mechanisms may be participating in IES excision. The authors identified the putative ATP-dependent chromatin remodeler ISWI1 as a novel factor participating in IES elimination. Knockouts of ISWI1 were defective in IES elimination. In particular, in ISWI1 KD cells, there was an increase of usage of alternative TA boundaries within a "forbidden zone", a size of IES absent in wild type cells, presumably due to nucleosome occupancy sterically hindering access of elimination machinery. Their model and conclusions were also supported by nucleosome density mapping experiments in ISWI1 KD and other mutants. These findings will be of broad interest to those in the ciliate community and DNA elimination fields.

Minor revisions:
When the authors state "Indeed, alteration of histone modifications, specifically H3K27me3 and H3K9me3, is associated with silencing EZL1 and PTCAF1, affecting the excision of most IESs in Paramecium", they could clarify what "silencing EZL1"

means.

For Figure 1E, it might be helpful to have a positive control like PGM-KD, but the data in 1F make clear the similarities between PGM KD and ISWI KD.

Fig 3D and associated text- Label the X-axis as to what samples are being compared. Clarify what the negative control was for the pulldown.

For Supplement S3A&B, label internal and external, respectively

Non-essential suggestions:
It should be discussed how the loss of an ATP-dependent remodeler like ISWI1 seemingly promotes nucleosome displacement and results in movement of nucleosomes, particularly when excision machinery is gaining access to alternative TA boundaries in the "forbidden peak" region. Perhaps introduction of HDAC inhibitor (like sodium butyrate) may counter or accentuate the impact of ISWI KD on IES deletion?

With respect to Fig 2. Why might ISWI1 KD not correlate with PTCAF KD and EZL1 KD?
Why do the authors think the data for the two ISWI KD are divergent around the IES retention score starting about .5?

As the authors stated, a direct protein interaction between ISWI1 and Ptiwi01 should not strongly be implied given the 1% formaldehyde crosslinking used in the pulldown experiments.

Referee #3:

This manuscript is a follow-up on an RNAi screen having identified genes up-regulated during self-fertilization of Paramecium tetraurelia. One of the hits was ISWI1, a homolog of the well-characterized mammalian SWI/SNF-family member ISWI, involved in chromatin remodeling. The authors convincingly show that out of the 5 ISWI family members encoded by P. tetraurelia, only ISWI1 expression peaked during the phase of fragmentation, when IES DNA elimination occurs in the "MACronucleus" to maturate the genome. Transgenic tagged ISWI1 was also shown to localize exclusively to the developing MAC. Inactivation of ISWI1, but not other isoforms, resulted in extensive cell death and retention of IESs. This was documented both by PCR at specific loci and by genome-wide DNA-seq. The latter also allowed to detect a lack of precision in the sites of excision, and revealed cleavage at sites previously shown to be sterically hindered under wild-type conditions. The authors also show an interaction of ISWI1 with a Piwi homolog possibly required for the accumulation of scnRNAs. Finally, the authors use DNAse-seq to illustrate that nucleosome positioning is enhanced in the absence of ISWI1 and that this positioning occurs mostly at IESs that resist excision.
This is a well-controlled series of experiments that convincingly show an implication of ISWI1 in the programmed genome rearrangements occurring during development of the somatic macronucleus, affecting both its efficiency and its precision. It also gives some mechanistic insight by documenting the impact of nucleosome positioning on the efficiency of the excision. Overall, it provides an important illustration of the impact of chromatin on the maturation of the MAC.

Minor points:
- The reading of Figure 4 is really laborious. May be bigwig files displayed in a genome browser over regions larger than those depicted in panels G and H would make the data easier to grasp.
- The English is mostly grammatically sound, but the choice of words is sometimes awkward. I would recommend editing by a native English speaker.

Referee #1:

Singh et al. describe the molecular function of a new component of the DNA elimination pathways in Paramecium. Paramecium serves as a model for genetics and epigenetic phenomena for a long time, one of the most prominent mechanisms described is the scnRNA pathway which transmits parental information about the DNA content to progeny. As exciting this mechanism is, in particular the transgenerational transport of RNA in association with its function to alter the DNA content of F1 Macronuclei, this mechanism still holds many secrets.
Singh et al. solve one of those. It was long described that domesticated transposases cleave DNA of the IES elements in the zygotic nucleus to form a functional gene unit. This paper here adds novel information about the removal of nucleosomes to allow the endonuclease to access the DNA.
As this paper clearly adds important new information and the general subject of transgenerational inheritance is of overall importance, this work adds a significant contribution to the understanding of genome editing in ciliates as well as to the general understanding to the role of non-coding RNA controlling epigenetics and genetics. The authors summarize their new data into a new model for IES excision which extents previous models by the nucleosome data. Being more realistic,

the new more model now can explain for the first time for IES size distribution and thus for evolutionary scenario of IES development.

I add below some points to improve the conclusions and clearness of the paper.

Major points:

1. I struggle with the normalization of reads for the nucleosome density. The authors use co-feeding and correctly argue that dilution of the dsRNA trigger causes milder silencing. They use therefore co-feeding of PGM with ND7, an unrelated gene as a control for ISWI/PGM co-silencing. (By the way, page 13 introduces PGM-KD alone without co-silencing, which confused me during the first reading).

Thanks for catching this. We corrected it to *ND7/PGM*-KD.

Ok then, Suppl Fig. S4 clearly shows that IES retention is much higher in the PGM/ISWI KD compared to the PGM/ND7 control. This is first of all reasonable.

But then, I cannot follow the normalization applied: "DNAse-seq reads normalized by DNA-Seq isolated from new Macs." Does it mean that parts of the cultures were grown to vegetative cultures and undigested classical Mac genomes were sequenced? Please explain more in detail

and also for a broad readership not necessarily familiar with Paramecium.

Since there is variability in DNA retention of IESs, between IESs, and between knockdowns, this needs to be accounted for. The way we did this was to normalize the DNase-seq coverage by the DNA-seq coverage. MAC isolations from *Paramecium* from the same cultures were split into two, and then either the DNase-seq library preparation protocol was applied or the DNA-seq one.
We changed the sentences in Materials and Methods to make this clearer: “Half of the nuclear pellet was then recovered in 100 µl of resuspension buffer (10 mM Tris-HCl, pH 7.4, 10 mM MgCl2) for MAC DNA isolation and sequencing (DNA-seq). The other half of the pellet was used for nucleosomal DNA isolation (DNase-seq). “

The Figure legend also adds another factor to address, the contamination with parental Mac DNA. Please explain the details of how normalization works for this aspect.

Thanks for this consideration. There may be some parental MAC DNA in the sequencing. This is why we did not analyze reads mapping to the MAC genome (since it is impossible to distinguish their source), but

rather those that map to IESs. The amount of IES DNA from the parental MAC is negligible compared to that from the knockdowns (compare Figure 2A vs Figure EV4A), both of which use the same nuclear isolation procedure. Thus we did not explicitly normalize this.

To the relevant methods section, we added:
"Note that the amount of IES DNA from the parental MAC is negligible compared to that from the knockdowns (compare Figure 2A vs Figure EV4A), both of which use the same nuclear isolation procedure. Thus, no explicit normalizations were applied to account for parental MAC DNA."

We updated a sentence in the results to make it clearer why and how we chose to examine reads that map to IESs only:

"Given the constraint that a minimum of 9 bp of a read needs to match to an IES, and that some reads mapping to the flanking MDS regions may be derived from the parental MAC, it does not currently seem prudent to obtain accurate nucleosomal positioning for short IESs."

2. Second, I have questions to which extent the two library preparation, more precisely the two fragmentation methods, differ in their biases. The DNAse will surely have a bias for GC content, then it's the question to which extent this is reflected by the normalization control. I totally agree with many other papers that the digestion of naked DNA instead of chromatin is not always the right control for the bias of MNase like analyses as for instance only a certain size fraction is analyzed. I miss an analysis and a more detailed description of the size distributions of digested DNA and the of the library. Maybe also a the inclusion of a nucleosome prediction by DNA sequence would shed light into the nature of IESs, Fig. 4H suggests a phased nucleosome pattern it would be interesting to see whether this is due at least to some extent to cis-factors in the DNA sequence.

We are not 100% sure about all the queries here but have attempted to address them as best we understand. We do not think possible DNase base composition biases impact our conclusions since we were not concerned so much about what the normalized nucleosome densities were for individual IESs, but rather the relative differences were between different IESs or classes of IESs. Furthermore, we have not observed any association between overall IES base composition and the effects on IES retention due to knockdowns of genes involved in genome

reorganization, just with the few bases at their ends. These bases comprise a small fraction of the total IES length. Hence, any biases related to them would not have close to the effect size observed for nucleosome densities between IES classes (e.g. Fig 4A and 4B).

We included histograms for the outer distances of paired-end reads (i.e. DNA fragment sizes before adapter ligation) as figures EV4B and EV4C. Two different sequencing library preparation methods were used since two different genome centres were responsible for this. For the *NOWA1/PGM*-KD experiment, a sharp lower cut-off in sizes was produced due to size selection (Fig EV4C). We amended the description in Materials and Methods accordingly. In both cases, we selected a size range (100 and 175 bp) that should primarily cover mononucleosomes for subsequent analyses. Between *ISWI1/PGM*-KD and *ND7/PGM*-KD, there appear to be differences in the relative mono- to di-nucleosomal distributions. In the future, we want to conduct more replicates and related experiments for other knockdowns to determine whether there is a biological reason for this or merely a technical one (e.g. slight variation in DNA-to-DNase ratio).

We decided to remove Figure 4G and Figure 4H. See the response to reviewer 3 relating to this. We think there are good reasons to avoid

analyzing nucleosome densities in detail for individual IESs. See our response to your 4th query regarding phasing.

3. I have another concern: what about IES-concatemers? If Pgm silencing is not 100% efficient due to co-silencing, a certain percentage of reads in digested DNA could come from concatemers, or should they absent from nucleosomes? Being transcribed they should have some nucleosomes, at least the larger ones. Could one screen for concatemers in the libraries, maybe also for some individual ones as indicators.

IES concatemers are eliminated from our analyses by the strict read mapping procedure, which will not permit extended mismatches as would result from concatemers. Since concatemers are relatively random combinations of IESs, coverage of each combination is low. Consequently, we would expect nucleosomal coverage of such combinations to be very low - too low to perform any sort of reliable analysis in our opinion. On the other hand, in the future, if suitable methods and targeted sequencing of IES circles (long enough to contain a nucleosome) were to be used, this might yield sufficient data.

4. Maybe I missed, but Figs 4G and H are not included in the main text. Please enlarge the X-axis scale so that one can directly identify the size of the IESs. For the large IES, it seems that ISWI silencing inhibits removal of nucleosomes except the MDS marginal ones directly flanking the IES, let's say -1 and +1 of the MDSs in reference to the IES. As I understand, these are individual IESs? This is not explained.
Is this effect of the -1 and +1 nucleosomes true for all longer IESs? Could this be related to the Ku70/Ku80 binding? I think the deeper analysis of this could result in deeper insight in the mechanism, related to IES recognition (see later comments). Maybe it's worth to analyze all IESs like this thinking about a good visualization for that.....

Though we lack gene predictions for IESs, from a BLASTX search of the longer IES shown in Figure 4H, we observed some similarities to predicted proteins in *P. pentaurelia* (top match, e-value 1e-20). So, it’s possible there are some genes, or pseudogenes that are being transcribed in this region, and hence what appears to be nicely phased nucleosomes. Shorter IESs are less likely to encode genes or pseudogenes and to be transcribed.

We actually did want to examine distributions of nucleosomes at IES boundaries. Unfortunately, for the reasons described above in response

to the question about contamination from the paternal nucleus, it simply isn’t possible to get fair estimates of this distribution because our procedure for general nucleosomal analysis avoids problems caused by paternal MAC DNA by excluding reads that map to regions flanking the IESs without bases that map within them. Thus one would systematically underestimate nucleosomal densities in the vicinity of IES boundaries on the IES side (+1) with our procedure, and it isn’t possible to know what they really are on the MDS side (-1). Further complicating matters, relatively few IESs are long enough (like the example in Figure 4H) to examine trends like the presence of multiple nucleosomes and phasing. So, we are hesitant to investigate finer details of IES nucleosomal positioning at this stage. Consequently, we wrote, “it does not currently seem prudent to obtain accurate nucleosomal positioning for short IESs” when we commented on the nucleosome density distributions. We think it will be more appropriate to investigate such details in the future with a greater representation of longer IESs in *Paramecium*, enabled by long-read sequencing.

Furthermore, to obtain nucleosomal phasing information for longer IESs, it will also be essential to obtain information about potential gene transcription, particularly transcription start sites (TSSs). TSS data exists, but it would take some time for us to investigate this thoroughly.

Though it may be challenging to inspect IES boundaries, in future we do think it will be informative to examine nucleosomal distributions within longer IESs in conjunction with RNA-seq.

To avoid confusion about boundary densities and because there are more general nucleosomal density distribution graphs (see comment to reviewer 3), we decided it would be best to remove Figures 4G and H.

5. This section of the DNAse results is difficult to follow. Page 13-15 are not straight forward, the discussion of the co-feeding interrupts the flow and as the main related Figure to that with the IES retention score of the PGM co-feeding is in the Supplementary data. I agree its necessary to include the RNAi efficiency, but a re-arrangement here would make it much easier for the reader.

Thanks for this suggestion. We shifted text on why dilution effects on RNAi efficiency are expected in Methods. This should aid the flow of the arguments.

6. I am not sure if I understand the meaning of the inclusion of the Nowa1/2/Pgm knock down analysis. As I understand Nowa1 acts upstream of ISWI and Pgm, this its inactivation leads to less ISWI

activation and thus to similar to the ISWI knockdown. I am asking whether the entire comparison of IES retention rates in relation to genome rearrangement components on the individual aspects of co-silencing (triple with Pgm) is really meaningful for the interpretation of ISWI/Pgm RNAi in relation to ND7/Pgm. If it is just a control for double silencing then please make this more clear, these long sentences like page 15 second paragraph cannot be followed, at least by myself.

We moved the text justifying co-silencing to Materials and methods, which should allow the main arguments to be followed more easily. We split the long sentence into two. *NOWA1/2/PGM*-KD does serve as an additional control for silencing efficiency, but it also serves more than that. It shows (i) there is consistently a difference in nucleosomal density distributions associated with knockdowns of proteins involved in genome reorganization; (ii) it's not just a homolog (ISWI1) that's likely involved in nucleosome positioning as it is in other organisms, but also factors implicated in interactions with sRNAs (Nowa1/2); (iii) the effects between the controls and these knockdowns are much less pronounced than the differences we observe with respect to IES retention scores.

Minor points:

1. In the introduction and in the discussion the authors mention, that no evidence shows H3K927me3/9me3 at IESs. That's right. It is claimed that these marks are also present in the old Mac, their loss in Ezl1 silencing may alter gene expression and this expression of genes involved in DNA elimination. That's indeed possible, but the authors should not leave the reader alone with that but offer then a hypothesis what's the scnRNA doing instead of triggering heterochromatic marks. Their model does not involve histone modifications at all. Would the author's alternative model for IESs histone modifications be that the scnRNA in Ptiwi1 directly triggers removal of the nucleosome independent of histone methylation? This should be discussed and involved in the conclusions. In this context, previous papers reported about a classification of IESs due to their genetic requirements for excision. Do scnRNA independent IESs, I think these are the old ones, show ISWI sensitivity? Would that mean that ISWI can be attracted to IESs independent of scnRNAs?

The reviewer is correct in observing that we do not implicate the classical histone marks in *Paramecium* IES excision, particularly in the context of short IESs. At least not directly. We simply do not have the data to analyze this aspect. Thus, we wish to remain relatively neutral about their possible role and maybe other modifications that might be

more relevant in the context of *Paramecium*. As we indicated, we are wary of possible knock-on effects related to unintended gene expression changes associated with the silencing of genes responsible for these marks. In fact, current literature seems to be pointing to more of a role for these histone marks in suppressing IESs containing identifiable transposon remnants rather than in IES excision in general. We feel the dust needs to settle a bit before we can clarify the role of these modifications.

We note that the original simple model illustrating *Paramecium* IES excision (Arnaiz et al. 2012) also did not show histone modifications (nor any other proteins, for that matter). Nevertheless, it seemed a sufficient foundation to explain salient features, particularly the IES length distribution. As we dig deeper, this model will need to be refined, and the molecules involved eventually placed.

As judged by correlations of gene knockdowns, ISWI1 dependence of IESs (ISWI1-KD IRS) is positively correlated with scnRNAs and iesRNAs (DCL2/3-KD and DCL5-KD), but has the highest correlation coefficient with the triple knockdown of these genes (DCL2/3/5-KD). So, yes, we think some IESs whose excision does not depend on scnRNAs are dependent on iesRNAs instead.

We added a paragraph to the Discussion describing our reluctance to invoke histone modifications in the excision of *Paramecium*'s (mostly short) IESs.

2. In context of the latter comment: Ptiwi11 was also found the MS/MS data in Suppl. Table 2. That's amazing. Could one speculate that also the IES RNAs could target ISWI to the IES? This would be a support of the former hypothesis that the IES RNAs cause a feed forward loop for efficient IES excision.

Yes, as argued in our previous response, it makes sense that iesRNAs are also involved together with ISWI1. It is encouraging that we observe Ptiwi11 in our mass spectrometry data. We have added a comment regarding this in the Discussion.

3. Again, in context of the first comment here (I feel that some aspects of the literature should be included into the discussion), Miro-Pina et al. 2022 showed the interaction between Ptiwi09 and Ezl1 and components. I am aware that this paper mainly addresses TE elements and the methods for detection of interaction partners are not comparable and its never possible to discuss a protein NOT found in MS. But could there be

a distinction between Ptiwi1 and Ptiwi09 the latter responsible for histone modification, Ptiwi01 for ISWI?

Ptiwi01 and Ptiwi09 are 99% identical, with the 8 amino acids differing between them all biochemically similar. We double-checked, and the majority of peptides from mass spectrometry are shared between these proteins in the ISWI1 pulldown. We think Ptiwi01 and Ptiwi09 are probably functionally identical. To the manuscript, we added in parentheses: “or Ptiwi09, since most peptides from the mass-spectrometry analysis are shared between these almost identical proteins”. We also replaced other instances with Ptiwi01/Ptiwi09 for the same reason. At this stage, we do not know why these proteins are found as potential interacting partners of the PRC2 complex as well as of ISWI1, but this is worth considering in the future.

4. In order to make this paper better to follow for a broader non ciliate audience, the introduction of the forbidden peak on page 9 cannot be understood by an explanation in a single line. Please elaborate this more in detail here, to allow the reader to follow your attention to these IESs before you come to the model.

Thank you for the suggestion; we expanded the argument/model relating to the forbidden peaks, which better highlights their importance in our investigations.

5. Please explain the properties of Atlantis DNAse in contrast to classical MNAse.

Atlantis dsDNase is an endonuclease specific to double-stranded DNA that cleaves phosphodiester bonds yielding oligonucleotides with 5'-phosphate and 3'-hydroxyl termini. On the other hand, MNase is an endonuclease that can cleave single-stranded and double-stranded nucleic acids (RNA and DNA), and is more specific for single-stranded nucleic acids. Furthermore, MNase cleavage is biased towards AT-rich and AU-rich sequences (https://www.zymoresearch.de/products/atlantis-ds-dnase). We added a description to this effect to Materials and Methods.

6. Page 13: PGM is not the last step in DNA elimination; this is, I think the ligation of ends....

Thank you for catching this error. We removed the inaccurate description.

Referee #2:

The manuscript "Chromatin remodeling is required for sRNA-guided DNA elimination" by Singh et al., significantly advances current knowledge about contributions of nucleosomal positioning to ciliate DNA excision of IES. The authors state that, while small RNAs and histone PTMs are appreciated as a major factor in guiding DNA elimination in many ciliates, in Paramecium, the smaller, subnucleosomal size of many IES, lack of known PTMs physically associated with IES, and additional proteins found to be required for excision suggests additional mechanisms may be participating in IES excision. The authors identified the putative ATP-dependent chromatin remodeler ISWI1 as a novel factor participating in IES elimination. Knockouts of ISWI1 were defective in IES elimination. In particular, in ISWI1 KD cells, there was an increase of usage of alternative TA boundaries within a "forbidden zone", a size of IES absent in wild type cells, presumably due to nucleosome occupancy sterically hindering access of elimination machinery. Their model and conclusions were also supported by nucleosome density mapping experiments in ISWI1 KD and other mutants. These findings will be of

broad interest to those in the ciliate community and DNA elimination fields.

Minor revisions:

When the authors state "Indeed, alteration of histone modifications, specifically H3K27me3 and H3K9me3, is associated with silencing EZL1 and PTCAF1, affecting the excision of most IESs in Paramecium", they could clarify what "silencing EZL1" means.

We use “silencing” and “gene knockdown” synonymously. At the beginning of the Materials and Methods section “Silencing experiments”, we added “gene knockdown” in brackets after the first mention of silencing to indicate this.

For Figure 1E, it might be helpful to have a positive control like PGM-KD, but the data in 1F make clear the similarities between PGM KD and ISWI KD.

Thank you for the comment. As the reviewer acknowledges, data in 1F shows the similarities and differences between PGM KD and ISWI KD. Also, the whole genome sequencing after the knockdowns confirms the observations of high retention of mobile element sequences for *ISWI1*-

KD. Therefore, we decided that having a negative control alone should suffice.

Fig 3D and associated text- Label the X-axis as to what samples are being compared. Clarify what the negative control was for the pulldown.

Thank you for pointing out this oversight; we have added labels in Fig 3D. On page 11, we did mention the controls: " As a control, we performed the same experiment on wild-type cells where we did not expect to see any pulldown of proteins with the HA affinity matrix. " For ISWI1-GFP and Ptiwi01 co-transformed cells, we mentioned: "Cells were transformed with ISWI1-GFP as a negative control to check whether the GFP tag and HA affinity matrix could interact non-specifically."

For Supplement S3A&B, label internal and external, respectively

We have added the labels as suggested.

Non-essential suggestions:

It should be discussed how the loss of an ATP-dependent remodeler like ISWI1 seemingly promotes nucleosome displacement and results in

movement of nucleosomes, particularly when excision machinery is gaining access to alternative TA boundaries in the "forbidden peak" region. Perhaps introduction of HDAC inhibitor (like sodium butyrate) may counter or accentuate the impact of ISWI KD on IES deletion?

Thank you for your careful attention to the arguments. We think you caught a logical flaw, and so we thought again about the model and decided to revise it. We hypothesize that *ISWI1*-KD is not promoting nucleosome displacement but rather that the removal of ISWI1 proteins leads to stalling of the nucleosomes blocking the excision of IESs at conventional boundaries and permitting excision at alternative, sub-optimal TA boundaries. Notably, alternative excision occurs in addition to the precise excision of the same IESs and occurs less frequently. So, one could consider that ISWI1 is necessary to properly position nucleosomes and prevent alternative excision errors. We adjusted the model figure and text in the Discussion accordingly.

We also think there are additional chromatin players as well as chromatin conformation itself that might play significant roles in ensuring the accessibility of correct IES boundaries. This requires future detailed investigations, perhaps using chromatin capture studies to analyze the physical form of chromatin during DNA elimination.

The suggested study using HDAC inhibitors is indeed an interesting suggestion. Still, to our knowledge, there are no studied HDACs that are specific to the time when DNA elimination occurs. Therefore, this study line would first require identifying such an HDAC and its potential role in DNA elimination. Furthermore, since HDACs would presumably also be involved in MAC chromatin state modification, it would be crucial to distinguish a direct function of HDACs from potential misregulation of MAC-encoded genes in the presence of such inhibitors.

With respect to Fig 2. Why might ISWI1 KD not correlate with PTCAF KD and EZL1 KD?

Why do the authors think the data for the two ISWI KD are divergent around the IES retention score starting about .5?

We think the reviewer is referring to Fig EV2A. This correlation matrix shows that there is some correlation between *ISWI1*-KD and *PTCAF1*-KD or *EZL1*-KD (correlation coefficients between 0.52 and 0.60). Certainly much less than between *PTCAF1*-KD and *EZL1*-KD (0.87), or between *ISWI1*-KD and *DCL2/3/5*-KD or *NOWA1/2*-KD (0.74 and 0.72, respectively). This is an interesting point to consider, though we do not

necessarily have the answer to this: even though PTCAF1, EZL1 and ISWI1 all encode genes whose homologs are involved in chromatin biology, their effects of IES excision in *Paramecium* are not exactly the same.

EZL1 and PTCAF1 were recently shown to be part of a PRC complex required for repressing transposable elements in *Paramecium* (10.1016/j.devcel.2022.03.014). One possibility would be that EZL1 and PTCAF1 affect chromatin associated with IESs in the developing MAC in additional ways to ISWI1. Alternatively, the effect of both these knockdowns on IES retention can possibly be attributed to the additional transcriptional misregulation of other essential genes during development, as both these proteins are present in the maternal and the as well as the developing macronucleus.

As data are from the replicates of ISWI1 knockdowns done at different times, we expect to see some degree of variation in IES retention simply because the effects can vary depending on the effectiveness of each knockdown. So, one of the two knockdowns had a slightly stronger effect than the other. Nevertheless, the correlation between the two knockdowns is very high (r= 0.91, Figure S2A). Hence, the same overall conclusions can be drawn from either.

As the authors stated, a direct protein interaction between ISWI1 and Ptiwi01 should not strongly be implied given the 1% formaldehyde crosslinking used in the pulldown experiments.

We certainly concur. At the end of the results section concerning the pulldown, we wrote: "Since all our samples were crosslinked before the IP assays, we cannot exclude the possibility that this interaction might be indirect, via chromatin." We added "though this may be an indirect action with chromatin intervening" to the statement about possible ISWI1-Ptiwi01 interaction in the discussion too.

Referee #3:

This manuscript is a follow-up on an RNAi screen having identified genes up-regulated during self-fertilization of Paramecium tetraurelia. One of the hits was ISWI1, a homolog of the well-characterized mammalian SWI/SNF-family member ISWI, involved in chromatin remodeling. The authors convincingly show that out of the 5 ISWI family members encoded by P. tetraurelia, only ISWI1 expression peaked

during the phase of fragmentation, when IES DNA elimination occurs in the "MACronucleus" to maturate the genome. Transgenic tagged ISWI1 was also shown to localize exclusively to the developing MAC. Inactivation of ISWI1, but not other isoforms, resulted in extensive cell death and retention of IESs. This was documented both by PCR at specific loci and by genome-wide DNA-seq. The latter also allowed to detect a lack of precision in the sites of excision, and revealed cleavage at sites previously shown to be sterically hindered under wild-type conditions. The authors also show an interaction of ISWI1 with a Piwi homolog possibly required for the accumulation of scnRNAs. Finally, the authors use DNAse-seq to illustrate that nucleosome positioning is enhanced in the absence of ISWI1 and that this positioning occurs mostly at IESs that resist excision.

This is a well-controlled series of experiments that convincingly show an implication of ISWI1 in the programmed genome rearrangements occurring during development of the somatic macronucleus, affecting both its efficiency and its precision. It also gives some mechanistic insight by documenting the impact of nucleosome positioning on the efficiency of the excision. Overall, it provides an important illustration of the impact of chromatin on the maturation of the MAC.

Minor points:

- The reading of Figure 4 is really laborious. May be bigwig files displayed in a genome browser over regions larger than those depicted in panels G and H would make the data easier to grasp.

We decided to remove Figure 4G and Figure 4H. The smoothing used in this figure creates problems in interpreting nucleosome densities at MDS/IES boundaries. Some of the nucleosomal reads on the MDS side can be from the old MAC, while most of the nucleosomal reads in the IESs are from the new MAC. More short IESs tend to be displayed at larger scales, amplifying the boundary problem. The preceding graphs are more general and show the key differences in nucleosome densities. We, therefore, decided to eliminate these two examples.

- The English is mostly grammatically sound, but the choice of words is sometimes awkward. I would recommend editing by a native English speaker.

Two native English speakers were involved in writing and editing; the first author is close to a native speaker. We rephrased some sections in response to comments from the reviewers, which should make some of the more challenging arguments easier to follow. After our changes in

response to the feedback from you and other reviewers, we made an additional pass to improve the clarity of the language.

Changes made in the manuscript

The changes made to the manuscript are documented as they appear in our response to the referee.

- Page 13, we corrected the control *PGM*-KD to *ND7/PGM*-KD.
- In Materials and Methods, subsection, “Nucleosomal DNA Isolation and Illumina DNA-sequencing”; we modified the description of DNA isolation with or without DNase treatment to as following:
  “Half of the nuclear pellet was then recovered in 100 µl of resuspension buffer (10 mM Tris-HCl, pH 7.4, 10 mM MgCl2) for MAC DNA isolation and sequencing (DNA-seq). The other half of the pellet was used for nucleosomal DNA isolation (DNase-seq). “
- In Materials and Methods, we added the description for library preparation for *NOWA1/2/PGM*-KD and its control since the sequencing and library preparation of these samples were done at a different sequencing facility. We also added figures in the Fig EV4 (B and C) corresponding to the library size distribution for all the DNase-seq libraries used in this study.
- In DNase seq analyses (Materials and methods), we added “Note that the amount of IES DNA from the parental MAC is negligible compared to that from the knockdowns (compare Figure 2A vs Figure S4A), both of which use the same nuclear isolation procedure. Thus, no explicit normalizations were applied to account for parental MAC DNA.”
- In Results (Page 13, Nucleosomal densities increase with IES dependence on ISWI1 and other genes involved in Paramecium IES excision), we added “Given the constraint that a minimum of 9 bp of a read needs to match to an IES, and that numerous reads mapping to the flanking MDS regions are derived from the parental MAC, it does not currently seem prudent to obtain accurate nucleosomal positioning for short IESs”
- We have removed Fig 4G-H to avoid confusion about boundary densities as explained in our response to reviewer one and three.

- We shifted the text about the dilution effects on RNAi from Results to Materials and Methods to aid the flow of arguments discussing nucleosomal density changed upon different knockdowns.
- While describing our observations related to nucleosomal density changes upon *NOWA1/2/PGM*-KD, we split sentences into smaller sentences so that it is easier to follow.
- We added more description on IES excision in the “forbidden peak”. In the Result section we added:
    - As can be seen in Fig 2D, the so-called “forbidden” second IES length peak (at ~40 bp; (Arnaiz *et al*, 2012)) is barely noticeable compared to the flanking IES length peaks. This was hypothesized as not being permitted by the biophysical constraints of DNA of this length, which prevents the two components of a conventional, domesticated PiggyBac transposase dimer from coming into the correct orientation needed for coordinated cleavage at both boundaries (Arnaiz *et al*, 2012). Since ISWI homologs are involved in nucleosome positioning in other organisms, we sought to determine if and how *ISWI1*-KD might impact IES excision precision.
- We added a pargraph in the Discussion section to explain the reson behind not including histone modifications in our proposed model.
    a. In Fig 5, we do not indicate the involvement of any histone modifications in *Paramecium* IES excision. Until more detailed analyses can be performed, showing the exact positioning of specific histone modifications in relation to IESs, we would prefer to avoid speculating about their role. On the other hand, it may also be possible for an alternative mechanism for IES targeting that does not invoke such modifications. Instead, it might also be possible that longer RNA transcripts across IESs promote binding of scnRNAs/iesRNAs, and more direct recruitment of the IES excisases. In any event, more detailed experiments will be necessary to examine nucleosomal properties, including positioning and modifications, and how they might influence IES excision.

- We modified our model system (Fig 5) slightly to improve our explanation of how we visualize the role of ISWI1 in regulating DNA elimination and modified the Fig legend accordingly. We also added a description in the Discussion to explain these changes. We added:
    - In the absence of the usual required nucleosomal shift, IESs can be excised at alternative TA boundaries, though they are still most frequently cut at the conventional boundaries (Figs 5B & 5C). In other words, ISWI1-KD assists in properly positioning nucleosomes, preventing alternative excision errors.
- We added a description about Atlantis DNase used in DNase-seq experiments in the Materials and methods section.
- We improved the overall language of the manuscript to make it easier to understand the data presented.

1st Revision - Editorial Decision 26th Aug 2022

Thank you for submitting your revised manuscript. We have received comments from the referees (please see below) and I am happy to say they now support publication. Therefore I would ask you to please resolve a number of editorial issues that are listed in detail below. Please use the document that the data editors have added their comments to for any changes (see below). If you have any further questions regarding the specific points listed or the final manuscript version, please feel free to contact me. Once these issues are resolved, we will be happy to formally accept the manuscript for publication.

Referee #1:

The authors addressed all of my concerns and nicely explained those which were caused by a lack of understanding the applied procedures. The manuscript is as such improved in the methodological details and easier to follow, I cannot find any arguments against this new version.

Referee #2:

This reviewer is satisfied with how the authors addressed the concerns.

Referee #3:

The authors have satisfactorily edited the manuscript.

2nd Revision - Editorial Decision 19th Sep 2022

Thank you for submitting the final revised version of your manuscript and addressing the remaining points. I am happy to inform you that we have now formally accepted your study for publication in The EMBO Journal.

EMBO Press Author Checklist

Corresponding Author Name: Prof. Mariusz Nowacki
Journal Submitted to: EMBOJ
Manuscript Number: Manuscript ID-2022-111839

USEFUL LINKS FOR COMPLETING THIS FORM
The EMBO Journal - Author Guidelines
EMBO Reports - Author Guidelines
Molecular Systems Biology - Author Guidelines
EMBO Molecular Medicine - Author Guidelines

Reporting Checklist for Life Science Articles (updated January
This checklist is adapted from Materials Design Analysis Reporting (MDAR) Checklist for Authors. MDAR establishes a minimum set of requirements in transparent reporting in the life sciences (see Statement of Task: 10.31222/osf.io/9sm4x). Please follow the journal's guidelines in preparing your
Please note that a copy of this checklist will be published alongside your article.

Abridged guidelines for figures
1. Data
The data shown in figures should satisfy the following conditions:
- the data were obtained and processed according to the field's best practice and are presented to reflect the results of the experiments in an accurate and unbiased manner.
- ideally, figure panels should include only measurements that are directly comparable to each other and obtained with the same assay.
- plots include clearly labeled error bars for independent experiments and sample sizes. Unless justified, error bars should not be shown for technical
- if n<5, the individual data points from each experiment should be plotted. Any statistical test employed should be justified.
- Source Data should be included to report the data underlying figures according to the guidelines set out in the authorship guidelines on Data

2. Captions
Each figure caption should contain the following information, for each panel where they are relevant:
- a specification of the experimental system investigated (eg cell line, species name).
- the assay(s) and method(s) used to carry out the reported observations and measurements.
- an explicit mention of the biological and chemical entity(ies) that are being measured.
- an explicit mention of the biological and chemical entity(ies) that are altered/varied/perturbed in a controlled manner.
- the exact sample size (n) for each experimental group/condition, given as a number, not a range;
- a description of the sample collection allowing the reader to understand whether the samples represent technical or biological replicates (including how many animals, litters, cultures, etc.).
- a statement of how many times the experiment shown was independently replicated in the laboratory.
- definitions of statistical methods and measures:
  - common tests, such as t-test (please specify whether paired vs. unpaired), simple χ2 tests, Wilcoxon and Mann-Whitney tests, can be unambiguously identified by name only, but more complex techniques should be described in the methods section;
  - are tests one-sided or two-sided?
  - are there adjustments for multiple comparisons?
  - exact statistical test results, e.g., P values = x but not P values < x;
  - definition of 'center values' as median or average;
  - definition of error bars as s.d. or s.e.m.

Please complete ALL of the questions below.
Select "Not Applicable" only when the requested information is not relevant for your study.

Materials

Newly Created Materials	Information included in the manuscript?	In which section is the information available? (Reagents and Tools Table, Materials and Methods, Figures, Data Availability Section)
New materials and reagents need to be available; do any restrictions apply?	Yes	Materials and Methods

Antibodies	Information included in the manuscript?	In which section is the information available? (Reagents and Tools Table, Materials and Methods, Figures, Data Availability Section)
For **antibodies** provide the following information:<br>- Commercial antibodies: RRID (if possible) or supplier name, catalogue number and or/clone number<br>- Non-commercial: RRID or citation	Yes	Materials and Methods

DNA and RNA sequences	Information included in the manuscript?	In which section is the information available? (Reagents and Tools Table, Materials and Methods, Figures, Data Availability Section)
**Short novel DNA or RNA including primers, probes**: provide the sequences.	Yes	Materials and Methods, Expanded View

Cell materials	Information included in the manuscript?	In which section is the information available? (Reagents and Tools Table, Materials and Methods, Figures, Data Availability Section)
**Cell lines:** Provide species information, strain. Provide accession number in repository **OR** supplier name, catalog number, clone number, and/**OR** RRID.	Not Applicable	
**Primary cultures:** Provide species, strain, sex of origin, genetic modification status.	Not Applicable	
Report if the cell lines were recently **authenticated** (e.g., by STR profiling) and tested for mycoplasma contamination.	Not Applicable	

Experimental animals	Information included in the manuscript?	In which section is the information available? (Reagents and Tools Table, Materials and Methods, Figures, Data Availability Section)
**Laboratory animals or Model organisms:** Provide species, strain, sex, age, genetic modification status. Provide accession number in repository **OR** supplier name, catalog number, clone number, **OR** RRID.	Not Applicable	
**Animal observed in or captured from the field:** Provide species, sex, and age where possible.	Not Applicable	
Please detail **housing and husbandry conditions**.	Not Applicable	

Plants and microbes	Information included in the manuscript?	In which section is the information available? (Reagents and Tools Table, Materials and Methods, Figures, Data Availability Section)
**Plants:** provide species and strain, ecotype and cultivar where relevant, unique accession number if available, and source (including location for collected wild specimens).	Not Applicable	
**Microbes:** provide species and strain, unique accession number if available, and source.	Yes	Materials and Methods

Human research participants	Information included in the manuscript?	In which section is the information available? (Reagents and Tools Table, Materials and Methods, Figures, Data Availability Section)
If collected and within the bounds of privacy constraints report on age, sex and gender or ethnicity for all study participants.	Not Applicable	

Core facilities	Information included in the manuscript?	In which section is the information available? (Reagents and Tools Table, Materials and Methods, Figures, Data Availability Section)
If your work benefited from core facilities, was their service mentioned in the acknowledgments section?	Yes	Yes

Design

Study protocol	Information included in the manuscript?	In which section is the information available? (Reagents and Tools Table, Materials and Methods, Figures, Data Availability Section)
If study protocol has been **pre-registered, provide DOI in the manuscript**. For clinical trials, provide the trial registration number **OR** cite DOI.	Not Applicable	
Report the **clinical trial registration number** (at ClinicalTrials.gov or equivalent), where applicable.	Not Applicable	

Laboratory protocol	Information included in the manuscript?	In which section is the information available? (Reagents and Tools Table, Materials and Methods, Figures, Data Availability Section)
Provide DOI OR other citation details if **external detailed step-by-step protocols** are available.	Yes	Materials and Methods, Figures

Experimental study design and statistics	Information included in the manuscript?	In which section is the information available? (Reagents and Tools Table, Materials and Methods, Figures, Data Availability Section)
Include a statement about **sample size** estimate even if no statistical methods were used.	Yes	Materials and Methods, Results
Were any steps taken to minimize the effects of subjective bias when allocating animals/samples to treatment (e.g. **randomization procedure**)? If yes, have they been described?	Not Applicable	
Include a statement about **blinding** even if no blinding was done.	Not Applicable	
Describe **inclusion/exclusion criteria** if samples or animals were excluded from the analysis. Were the criteria pre-established?<br><br>If sample or data points were omitted from analysis, report if this was due to attrition or intentional exclusion and provide justification.	Not Applicable	
For every figure, are **statistical tests** justified as appropriate? Do the data meet the assumptions of the tests (e.g., normal distribution)? Describe any methods used to assess it. Is there an estimate of variation within each group of data? Is the variance similar between the groups that are being statistically compared?	Not Applicable	

Sample definition and in-laboratory replication	Information included in the manuscript?	In which section is the information available? (Reagents and Tools Table, Materials and Methods, Figures, Data Availability Section)
In the figure legends: state number of times the experiment was **replicated** in laboratory.	Not Applicable	
In the figure legends: define whether data describe **technical or biological replicates**.	Not Applicable	

Ethics

Ethics	Information included in the manuscript?	In which section is the information available? (Reagents and Tools Table, Materials and Methods, Figures, Data Availability Section)
Studies involving **human participants**: State details of **authority granting ethics approval** (IRB or equivalent committee(s), provide reference number for approval.	Not Applicable	
Studies involving **human participants**: Include a statement confirming that **informed consent** was obtained from all subjects and that the experiments conformed to the principles set out in the WMA Declaration of Helsinki and the Department of Health and Human Services Belmont Report.	Not Applicable	
Studies involving **human participants:** For publication of **patient photos**, include a statement confirming that consent to publish was obtained.	Not Applicable	
Studies involving experimental **animals**: State details of **authority granting ethics approva**l (IRB or equivalent committee(s), provide reference number for approval. Include a statement of compliance with ethical regulations.	Not Applicable	
Studies involving **specimen and field samples:** State if relevant **permits** obtained, provide details of authority approving study; if none were required, explain why.	Not Applicable	

Dual Use Research of Concern (DURC)	Information included in the manuscript?	In which section is the information available? (Reagents and Tools Table, Materials and Methods, Figures, Data Availability Section)
Could your study fall under dual use research restrictions? Please check biosecurity documents and list of **select agents and toxins** (CDC): https://www.selectagents.gov/sat/list.htm	Not Applicable	
If you used a select agent, is the security level of the lab appropriate and reported in the manuscript?	Not Applicable	
If a study is subject to dual use research of concern regulations, is the name of the **authority granting approval and reference number** for the regulatory approval provided in the manuscript?	Not Applicable	

Reporting

The MDAR framework recommends adoption of discipline-specific guidelines, established and endorsed through community initiatives. Journals have their own policy about requiring specific guidelines and recommendations to complement MDAR.

Adherence to community standards	Information included in the manuscript?	In which section is the information available? (Reagents and Tools Table, Materials and Methods, Figures, Data Availability Section)
State if relevant guidelines or checklists (e.g., **ICMJE, MIBBI, ARRIVE, PRISMA**) have been followed or provided.	Not Applicable	
For **tumor marker prognostic studies,** we recommend that you follow the **REMARK** reporting guidelines (see link list at top right). See author guidelines, under 'Reporting Guidelines'. Please confirm you have followed these guidelines.	Not Applicable	
For **phase II and III randomized controlled trials**, please refer to the **CONSORT** flow diagram (see link list at top right) and submit the CONSORT checklist (see link list at top right) with your submission. See author guidelines, under 'Reporting Guidelines'. Please confirm you have submitted this list.	Not Applicable	

Data Availability

Data availability	Information included in the manuscript?	In which section is the information available? (Reagents and Tools Table, Materials and Methods, Figures, Data Availability Section)
Have **primary datasets** been deposited according to the journal's guidelines (see 'Data Deposition' section) and the respective accession numbers provided in the Data Availability Section?	Yes	Data Availability Section
Were **human clinical and genomic datasets** deposited in a public access-controlled repository in accordance to ethical obligations to the patients and to the applicable consent agreement?	Not Applicable	
Are **computational models** that are central and integral to a study available without restrictions in a machine-readable form? Were the relevant accession numbers or links provided?	Not Applicable	
If publicly available data were reused, provide the respective **data citations in the reference list**.	Yes	Data Availability Section
