# Supplementary material for: Chromatin remodeling is required for sRNA‐guided DNA elimination in Paramecium
Source: EMBO J. 2022 Oct 11;41(22):e111839. doi: 10.15252/embj.2022111839 (PMC9670198; doi:10.15252/embj.2022111839)
Supplement: Supplementary file 9 — Source Data for Expanded View [file EMBJ-41-e111839-s014.zip › Source_Data_EVfigures/SourceData_EV5.zip!/SourceData_EV5/SourceData_EV5.pdf]

Figure EV5 Support Data(1-3)

(1)

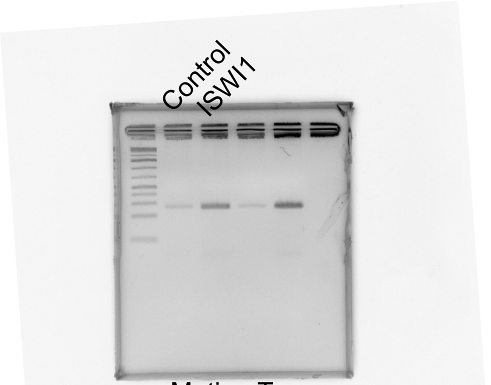

Mating Type

(2)

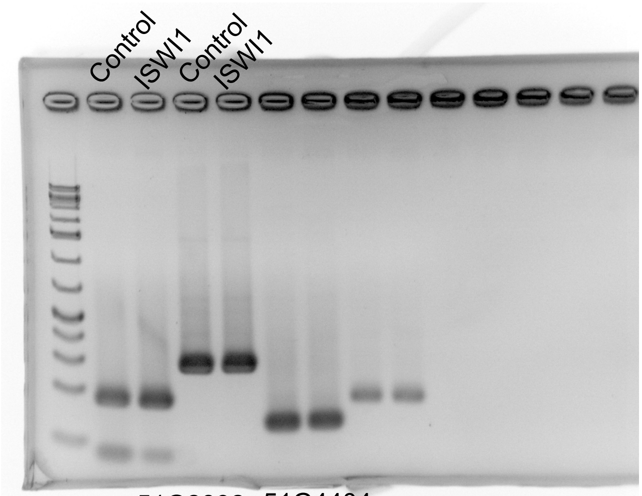

51G2832 51G4404

(3)

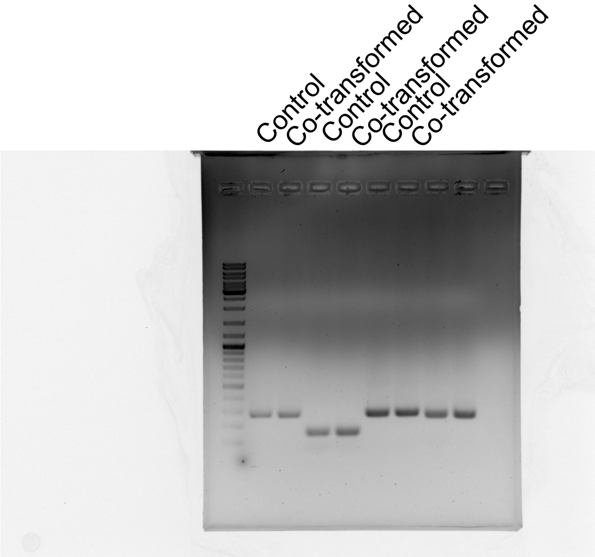

Mating Type, 51G2832, 51G4404
