# Supplementary material for: Chromatin remodeling is required for sRNA‐guided DNA elimination in Paramecium
Source: EMBO J. 2022 Oct 11;41(22):e111839. doi: 10.15252/embj.2022111839 (PMC9670198; doi:10.15252/embj.2022111839)

FigureEV1 Source Data

(1)

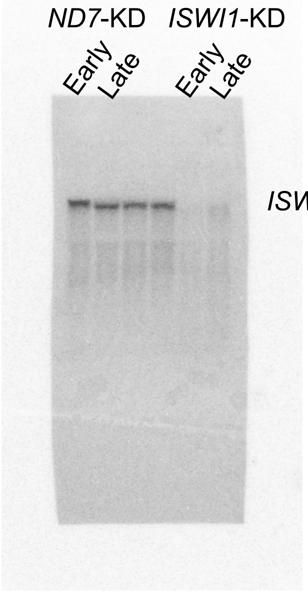

(2)

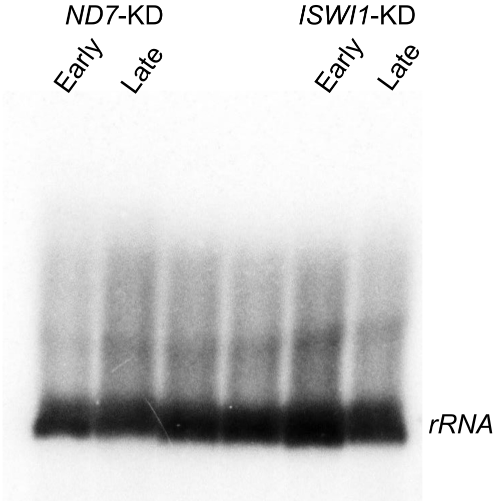

(3)

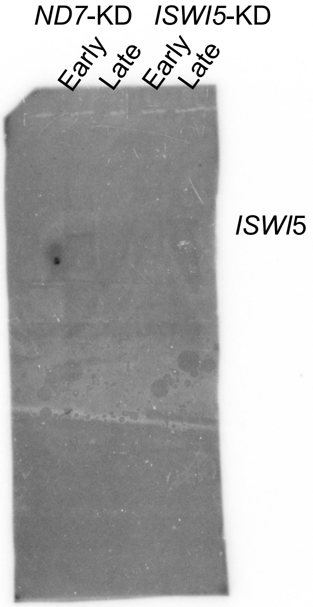

(4)

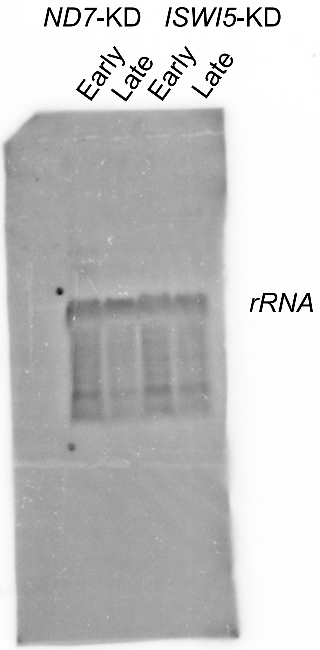

(5)

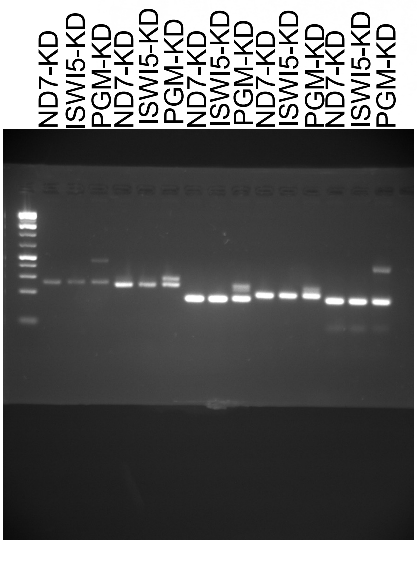

Mating Type, 51G11, 51G1413,
51G1832, 51G2832

(6)

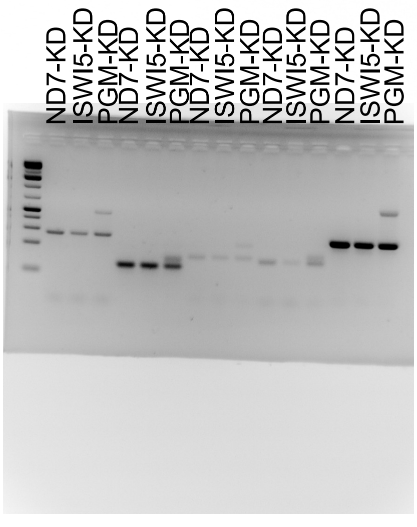

51G4404, 51G6447, 51A-712,
51A1835, 51A2591

(7)

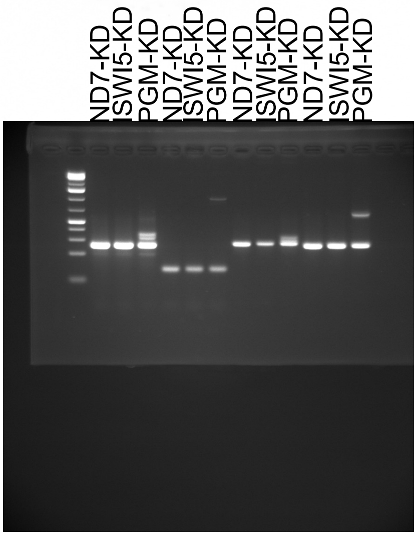

51A4404, 51A4578, 51A6435,
51A6649

FigureEV1 Source Data

(8)

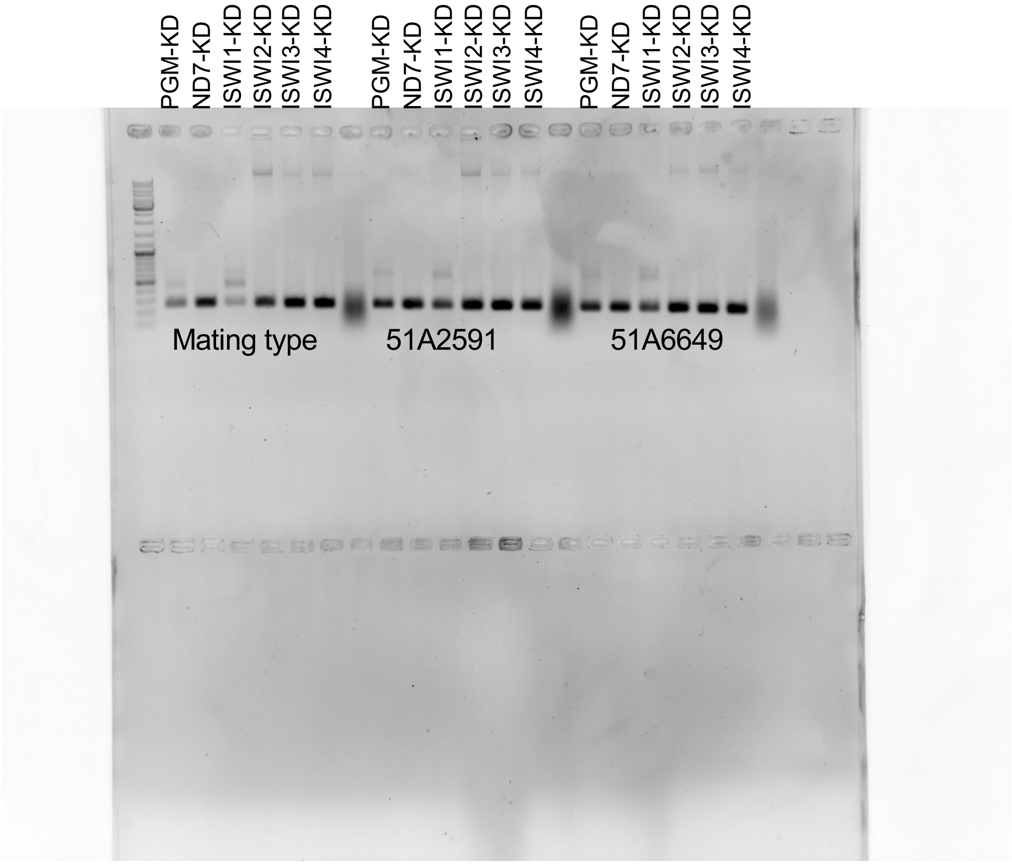

(9)

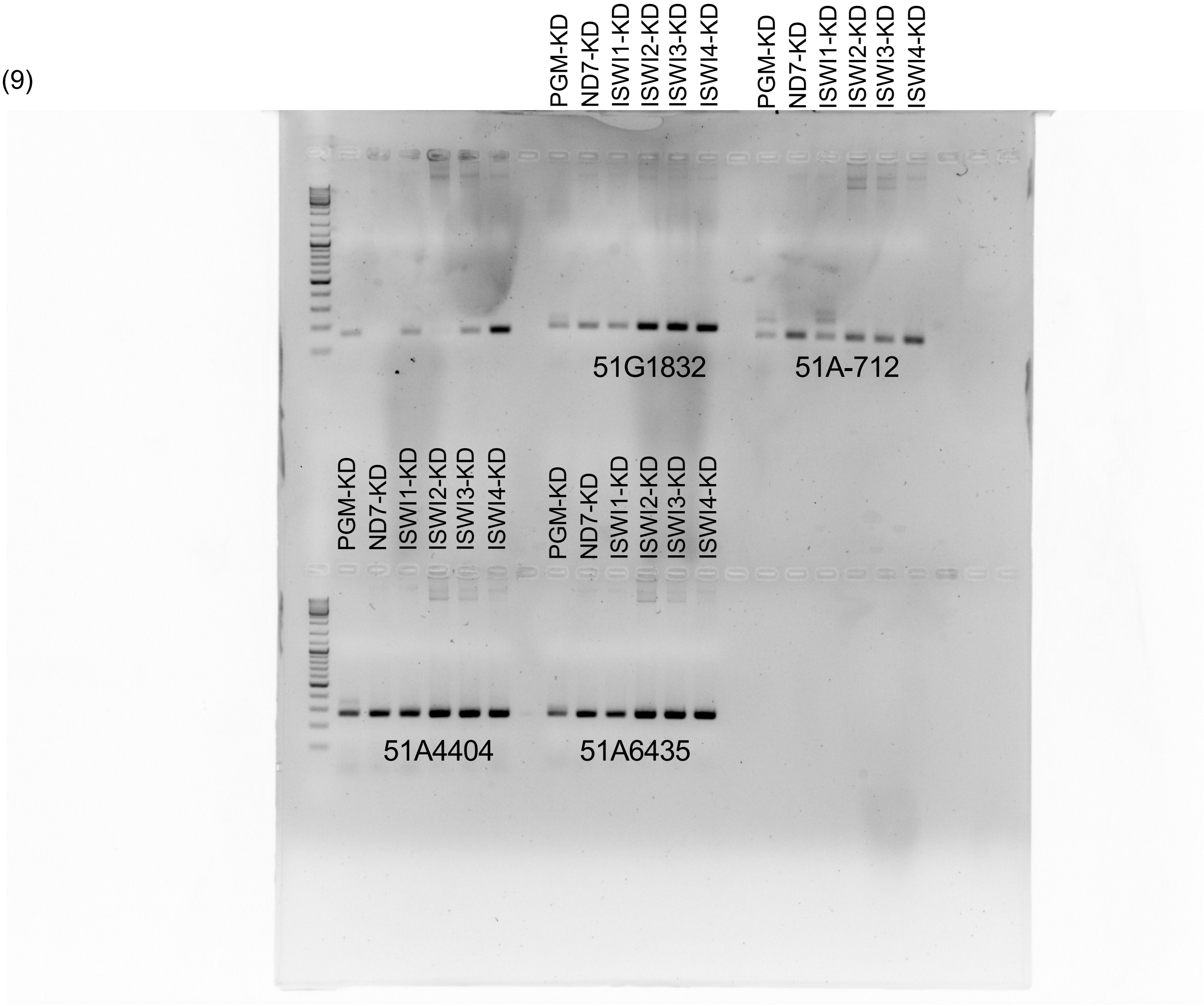

Supplement: Supplementary file 9 — Source Data for Expanded View [file EMBJ-41-e111839-s014.zip › Source_Data_EVfigures/SourceData_EV1.zip!/SourceData_EV1/SourceData_EV1.pdf]
